# Design, synthesis, and bioevaluation of diarylpyrimidine derivatives as novel microtubule destabilizers

**DOI:** 10.3389/fchem.2024.1447831

**Published:** 2024-07-25

**Authors:** Yutao Xiu, Yujing Zhang, Shanbo Yang, Lingyu Shi, Dongming Xing, Chao Wang

**Affiliations:** ^1^ Cancer Institute, The Affiliated Hospital of Qingdao University, Qingdao University, Qingdao, Shandong, China; ^2^ Qingdao Cancer Institute, Qingdao University, Qingdao, Shandong, China; ^3^ The Affiliated Cardiovascular Hospital of Qingdao University, Qingdao University, Qingdao, Shandong, China; ^4^ School of Life Sciences, Tsinghua University, Beijing, China

**Keywords:** microtubule destabilizer, combretastatin A-4, antiproliferative activity, pyrimidine, molecular docking

## Abstract

In this work, a series of new diarylpyrimidine derivatives as microtubule destabilizers were designed, synthesized, and evaluated for anticancer activities. Based on restriction configuration strategy, we introduced the pyrimidine moiety containing the hydrogen-bond acceptors as *cis*-olefin bond of CA-4 analogs to improve structural stability. Compounds **11a-t** exerted antiproliferative activities against three human cancer cell lines (SGC-7901, HeLa, and MCF-7), due to tubulin polymerization inhibition, showing high selectivity toward cancer cells in comparison with non-tumoral HSF cells, as evidenced by MTT assays. In mechanistic investigations, compound **11s** remarkably inhibited tubulin polymerization and disorganized microtubule in SGC-7901 cells by binding to tubulin. Moreover, **11s** caused G2/M phase cell cycle arrest in SGC-7901 cells in a concentration-dependent manner. Furthermore, molecular modeling analysis revealed that **11s** interacts with tubulin through binding to the colchicine site. In addition, the prediction of physicochemical properties disclosed that **11s** conformed well to the Lipinski’s rule of five. This work offered a fresh viewpoint for the discovery of new tubulin-targeting anticancer drugs.

## 1 Introduction

**FIGURE 1:**
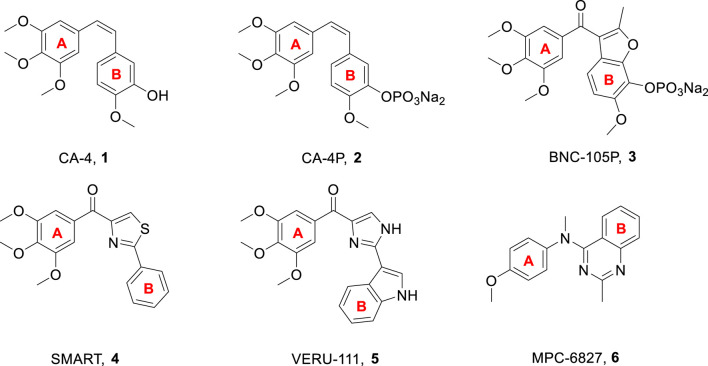
Chemical structures of some microtubule destabilizers.

Microtubules have been identified as an essential target for anticancer drug development. They play an important role in a variety of fundamental cell functions, including shape maintenance, intracellular transport, and cell division (Dumontet and Jordan, 2010; Steinmetz and Prota, 2018; Čermák et al., 2020). Microtubule targeting agents have been classified as microtubule stabilizers (taxanes and epothilones) and microtubule destabilizers (alkaloids and colchicine) according to the mechanism of interference with microtubule dynamics (Chen et al., 2010; Cao et al., 2018; Wang et al., 2021). The microtubule destabilizers have attracted considerable interest from medicinal chemists due to the largely successful clinical use of vinca alkaloids (Lu et al., 2012; Wang et al., 2018). In the past decades, many excellent microtubule destabilizers have been reported, such as combretastatin A-4 (CA-4, **1**, Figure 1), CA-4P (**2**, Figure 1), BNC-105P (**3**, Figure 1), SMART (**4**, Figure 1), VERU-111 (**5**, Figure 1), and MPC-6827 (**6**, Figure 1) (Pettit et al., 1989; Kasibhatla et al., 2007; Grossmann et al., 2012; Pal et al., 2015; Pérez-Pérez et al., 2016; Wu et al., 2018; Mahmud et al., 2020).

CA-4, a natural product that inhibits tubulin polymerization by interacting with the colchicine binding site on tubulin, was first isolated in 1989 from the bark of the South African willow tree, *Combretum caffrum* (Pettit et al., 1989). This *cis*-stilbene has been shown to exhibit excellent cytotoxicity against a variety of human cancer cell lines, including multi-drug resistant cancer cell lines (Mustafa et al., 2019; Paidakula et al., 2022). CA-4P (2, Figure 1), which is the soluble prodrug of CA-4, is now undergoing clinical trials as a combination therapy for several multi-drug resistant solid cancers (Pérez-Pérez et al., 2016). Owing to the structural simplicity of CA-4, many academic and industrial groups have carried out numerous structure-activity relationship (SAR) studies on this compound and its analogs. SAR studies have indicated that the presence of a *cis*-olefin bond and 3,4,5-trimethoxyphenyl as A-ring are crucial for producing potent potency (Hamze et al., 2020). CA-4 and other olefinic analogs tend to isomerize into inactive *trans*-forms during administration and storage. Thus, stabilizing the *cis*-olefin in the CA-4 structure is a key trend (Jian et al., 2020). In recent years, to improve structural stability, leading researchers have sought to replace the *cis*-olefin in the CA-4 structure with a heterocyclic ring to be used as a lead compound, while simultaneously creating a *cis* conformation for both the A-and B-ring. To date, different cyclic structures, such as three-, four-, five-, and six-membered heterocyclic rings, have been utilized to replace the *cis*-olefin structure of CA-4, producing favorable outcomes.

**FIGURE 2:**
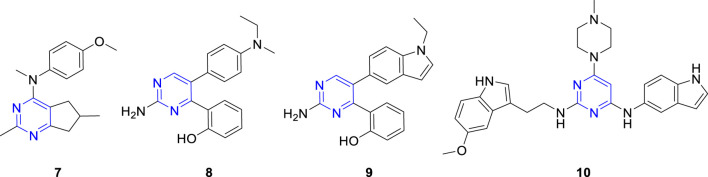
Chemical structures of some pyrimidine-based microtubule destabilizers.

Pyrimidine is an aromatic heterocycle with six members that comprises of two nitrogen atoms. Pyrimidine derivatives show great advantages due to the presence of nitrogen atoms, such as the ability to increase the basicity of the molecule, due to their basic properties and the possibility of the nitrogen atom to form strong hydrogen bonds with the target. In addition, pyrimidine derivatives are a topic of continuing research due to their ease of preparation and a wide range of potential pharmacological properties, which include bactericidal effect (Tan et al., 2022), anti-inflammatory activities (Shukla et al., 2016), and antitumor activities (Sana et al., 2021; Kang et al., 2023). Several pyrimidine-based microtubule destabilizers, including arylpyrimidine derivative (**7**, Figure 2), diarylpyrimidine derivative (**8**, Figure 2), arylpyrimidine-indole derivative (**9**, Figure 2), and indole-pyrimidine hybrid (**10**, Figure 2), had been produced and tested (Xie et al., 2009; Xie et al., 2011; Zhang et al., 2014; Hu et al., 2015).

**FIGURE 3:**
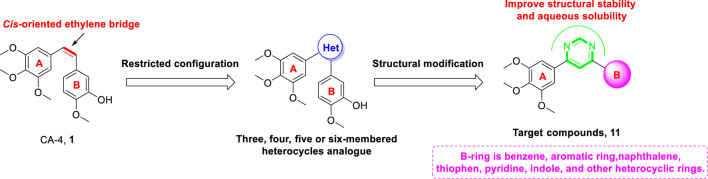
The rational design of target compounds.

The primary aim of this work was to design, synthesize, and biologically evaluate new diarylpyrimidine derivatives (**11**, Figure 3) with high binding affinity to tubulin, which can trigger antimitotic effect on cancer cells. To this end, we successfully replaced the *cis*-olefin linker of CA-4 with a pyrimidine moiety. We further explored the incorporation of the following different aromatic B-ring: aryl, thienyl, pyridine, indole, and naphthalene. Some of the newly synthesized compounds are with more structural stability than the reference CA-4. Their antiproliferative activities were studied in three human tumor cell lines. Normal skin fibroblast (HSF) cells were used to study toxicity in healthy tissues. Moreover, a specific compound, **11s**, was evaluated for tubulin polymerization, immunofluorescence staining, cell cycle analysis, induction of cell apoptosis, and molecular docking analysis.

## 2 Result and discussion

### 2.1 Chemistry

**SCHEME 1:**
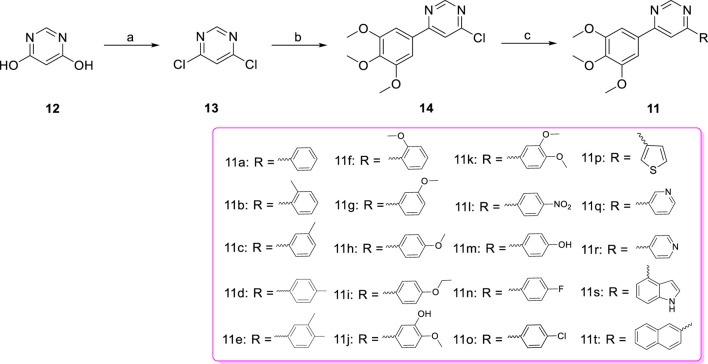
Reagents and conditions (a) POCl_3_, NEt_3_, reflux; (b) 3,4,5-trimethoxyphenylboric acid, Pd(PPh_3_)_4_, K_2_CO_3_, 1,4-dioxane/H_2_O, N_2_ atmosphere,110°C, M.W.; (c) Substituted phenylboronic acid, Pd(PPh_3_)_4_, K_2_CO_3_, 1,4-dioxane/H_2_O, N_2_ atmosphere,126°C, M.W.

The target compounds diarylpyrimidine derivatives **11a-t** were synthesized according to the procedure described in Scheme 1. Previous literature outlines that the 4,6-dichloropyrimidine (**13**) was synthesized using 4,6-dihydroxypyrimidine (**12**) as the starting material (Yang et al., 2022; Wang et al., 2023). The synthesis of 4-chloro-6-(3,4,5-trimethoxyphenyl) pyrimidine (**14**) was achieved through the Suzuki cross-coupling reaction between **13** and 3,4,5-trimethoxyphenylboric acid, in the presence of potassium carbonate and tetrakis (triphenylphosphine) palladium (Shi et al., 2022). Target compounds **11a-t** were obtained by Suzuki cross-coupling reaction between **14** and the corresponding arylboronic acids (Dufresne et al., 2007).

### 2.2 Biological evaluation

#### 2.2.1 *In vitro* antiproliferative activity

The MTT assay with CA-4 as a positive control was used to evaluate the antiproliferative activities of the newly synthesized compounds **11a-t** against three representative cancer cell lines (SGC-7901, HeLa, and MCF-7). Some target compounds with IC_50_ values in the micromolar range showed moderate potency against three cancer cell lines. Out of the tested compounds, **11s**, which contained an indole moiety as the B-ring, displayed the most potent antiproliferative activities against SGC-7901, HeLa, and MCF-7 cell lines with IC_50_ values of 12.0, 15.3, and 16.7 µM, respectively.

The SAR of the 20 target compounds have been summarized (Table 1). With phenyl or aryl moieties as the B-ring, **11a-11o** showed moderate to weak activities, and the introduction of electron-donating groups (EDGs), such as -CH_3_ (**11d**), -3-CH_3_-4-CH_3_ (**11e**) -OCH_3_ (**11g** and **11h**), -OCH_2_CH_3_ (**11i**), -3-OH-4-OCH_3_ (**11j**), on the para-substitution of B-ring, led to maintaining or increasing in antiproliferative activities. However, when electron-withdrawing groups (EWGs), such as -F (**11n**), -Cl (**11o**), and -NO_2_ (**11l**), were introduced on the para-substitution of B-ring, the antiproliferative activities were decreased, and the results indicated that EDGs located on the para-substitution of B-ring had better activities. We next introduced different rigid aromatic groups such as thienyl (**11p**), pyridyl (**11q** and **11r**), indolyl (**11s**), naphthyl (**11t**) into the B-ring to explore the effect of different skeletons on antiproliferative activities against three different cell lines. Amongst these, **11s** showed the best antiproliferative activity against SGC-7901 cells (IC_50_ value of 12.0 µM), suggesting that the volume and electronegativity of B-ring may be the important factors affecting activity.

**TABLE 1:** Antiproliferative activities of all compounds.

Compounds	(IC_50_, μM)^a^		
	SGC-7901	HeLa	MCF-7
**11a**	70.2	81.1	>100
**11b**	96.6	>100	>100
**11c**	>100	79.3	>100
**11d**	39.9	42.3	48.4
**11e**	61.2	66.8	73.5
**11f**	>100	>100	>100
**11g**	46.5	62.3	76.0
**11h**	24.8	31.3	35.7
**11i**	44.3	50.3	64.6
**11j**	19.4	21.5	27.9
**11k**	>100	76.9	>100
**11l**	>100	>100	>100
**11m**	84.9	>100	87.3
**11n**	>100	>100	>100
**11o**	>100	>100	>100
**11p**	40.2	31.9	60.5
**11q**	>100	>100	>100
**11r**	>100	>100	>100
**11s**	**12.0**	**15.3**	**16.7**
**11t**	>100	>100	>100
**CA-4** ^b^	0.098	0.87	0.12

^a^ IC_50_: the half maximal inhibitory concentration.

^b^ Used as positive controls.

Next, cytotoxicity test was performed with HSF to assess the cytotoxicity of **11s**, and CA-4 was used as the positive control. As can be seen in Table 2, the cytotoxicity of **11s** (IC_50_ value >100 μM) on HSF was significantly weaker than that of CA-4 (IC_50_ value 0.77 μM). The result showed that the cytotoxicity of **11s** was lower than that of CA-4.

**TABLE 2:** Cytotoxicity test of **11s** and CA-4 against HSF.

Compounds	(IC_50_, μM)^a^
**11s**	>100
CA-4	0.77

^a^ IC_50_: the half maximal inhibitory concentration.

^b^ Used as positive controls.

#### 2.2.2 Effect on tubulin polymerization

**FIGURE 4:**
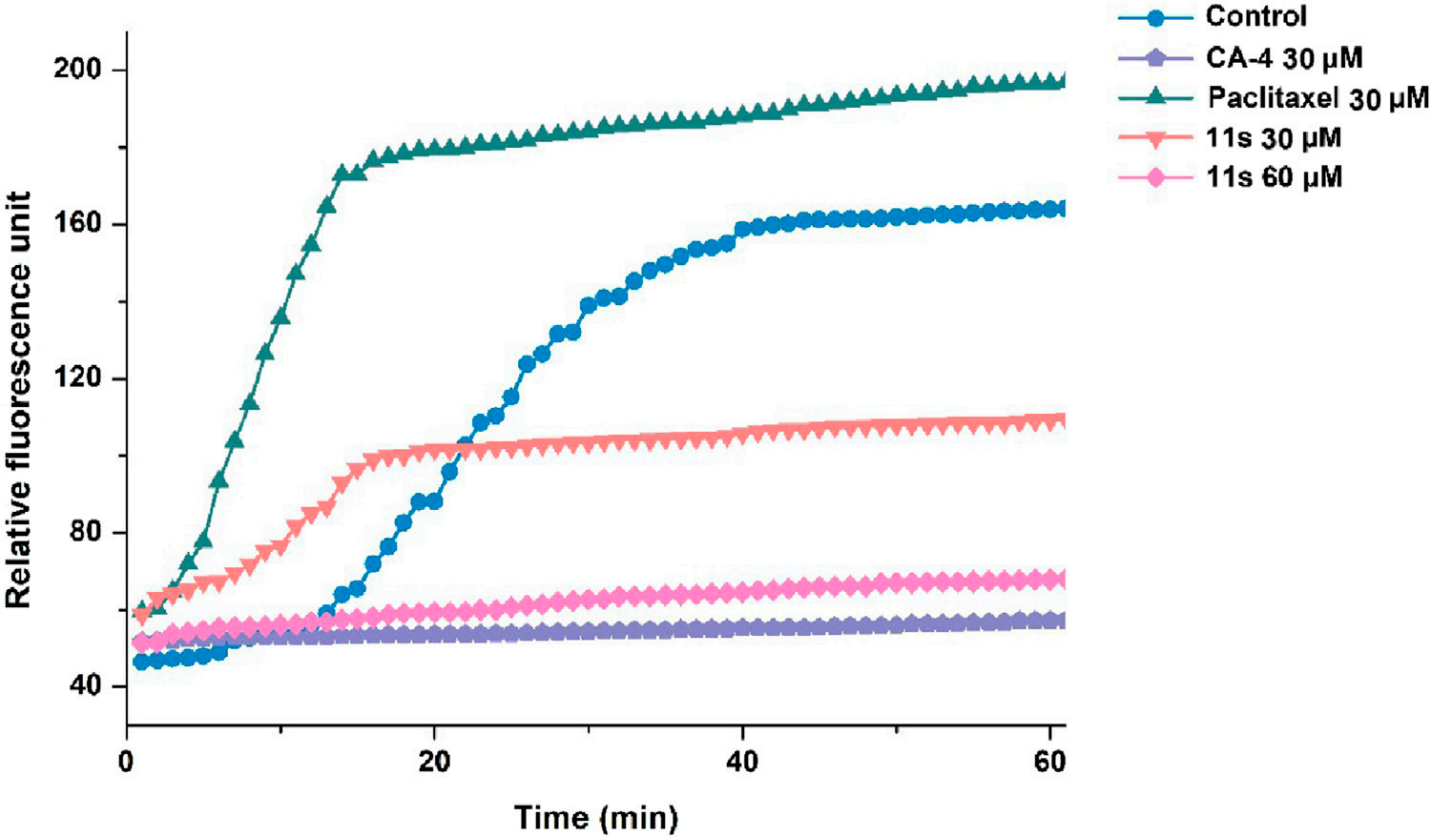
Effects of compound **11s** on tubulin polymerization.

For clarification of whether these 3-aryl-4-(3,4,5-trimethoxyphenyl) pyridines target the tubulin-microtubule system, we assessed the inhibition of tubulin polymerization using the most active compound, **11s**, alongside the negative control paclitaxel and the positive control CA-4. Figure 4 indicates that both **11s** and CA-4 were effective inhibitors of tubulin polymerization in comparison to the negative control, paclitaxel. Furthermore, **11s** was found to inhibit tubulin polymerization in a concentration-dependent manner. These results illustrated that **11s** inhibited tubulin polymerization in a manner similar to CA-4.

#### 2.2.3 Analysis of immunofluorescence staining

**FIGURE 5:**
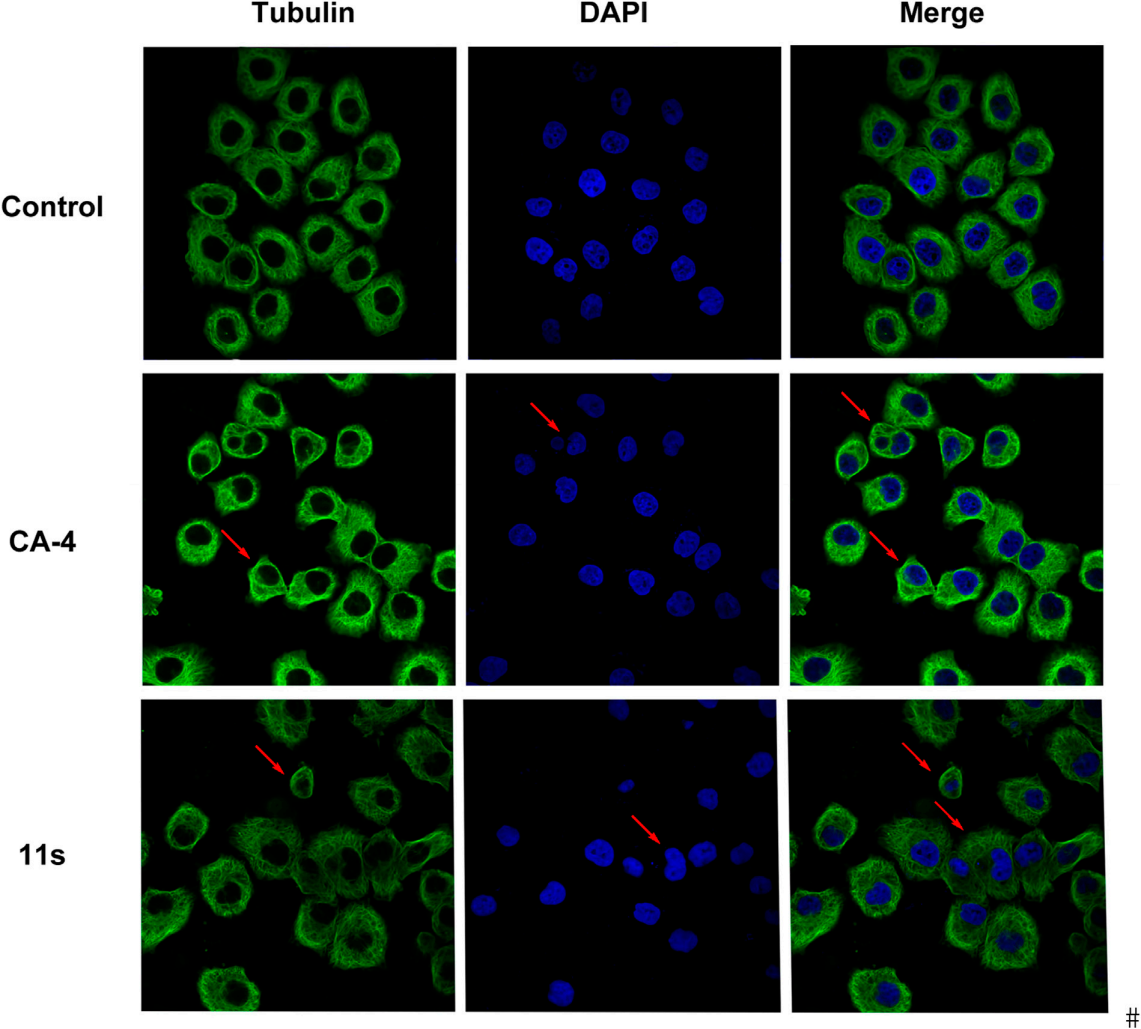
Effects of compound **11s** and CA-4, on the cellular microtubule network and microtubule reassemble by immunofluorescence.

Using CA-4 (0.10 µM) as a reference, we tested whether compound **11s** could destabilize microtubule dynamics in SGC-7901 cells using an immunofluorescence assay to verify the direct effects of **11s** on tubulin. Indirect immunofluorescence was also used to observe cellular microtubule structures. As shown in Figure 5, the cells treated with **11s** (12.0 µM) showed changes in the shape of the nucleus (blue) and the microtubule network (green) was constricted and disorganized in comparison with the control group. The results suggested that **11s** disrupted the cytoskeleton and inhibited microtubule assembly in a similar way to CA-4.

#### 2.2.4 Cell cycle analysis

**FIGURE 6:**
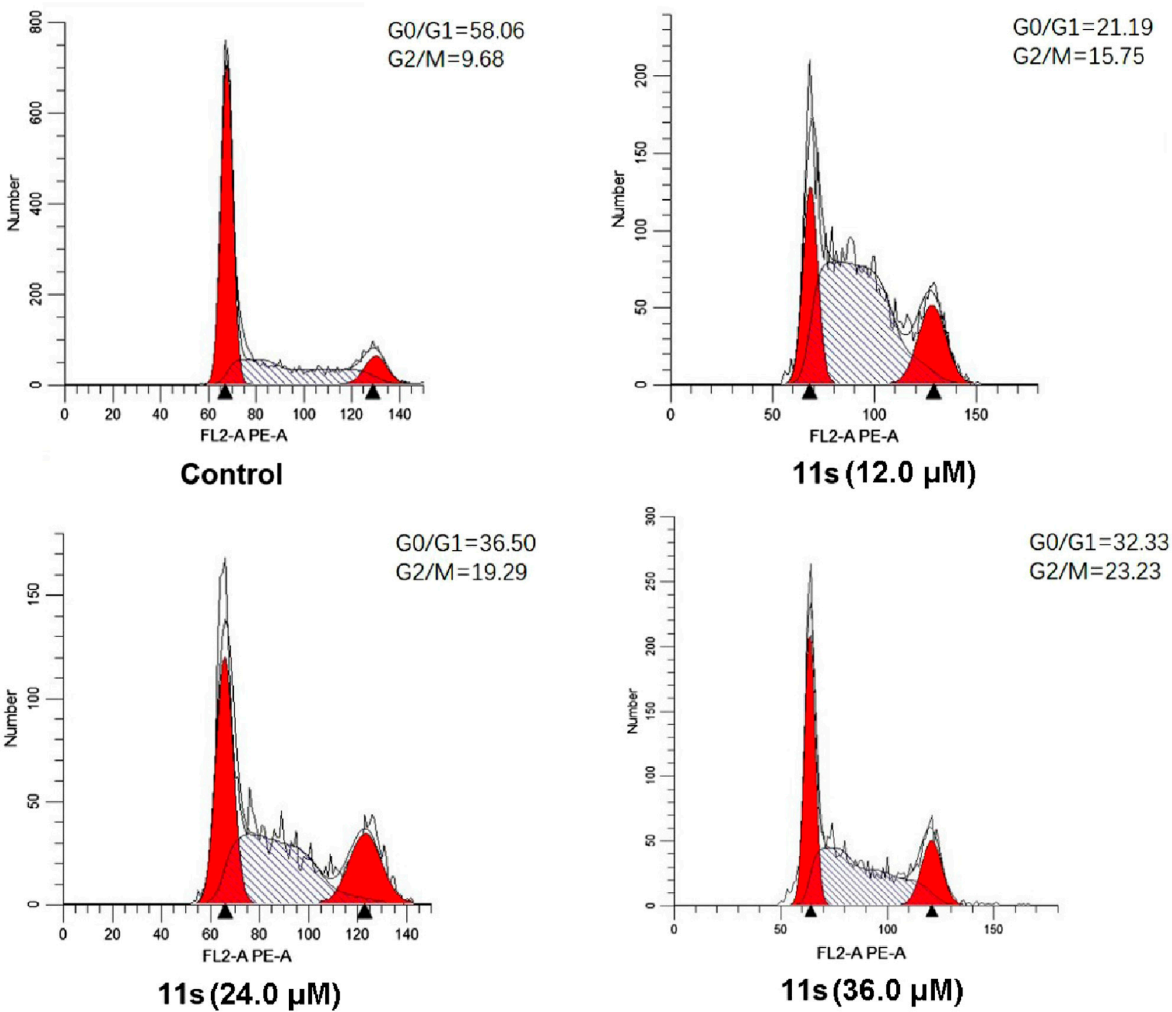
Effects of compound **11s** on cell cycle. SGC-7901 cell lines were treated with compound **11s** for 24 h.

The effects of the most promising compound **11s** on the cell cycle were investigated to further explore the biological target. SGC-7901 cells were treated with 12.0 µM, 24.0 µM, and 36.0 µM of **11s**, and the percentage of SGC-7901 cells in different cell cycle phases after treatment was analyzed by flow cytometry. As revealed in Figure 6, the percentage of SGC-7901 cells arrested in G2/M phase increased from 15.75% to 23.23% after treatment with the three concentrations of **11s** compared to the control (9.68%). Thus, **11s** could induce G2/M phase arrest in SGC-7901 cells in a concentration-dependent manner. The cell cycle distribution demonstrated that **11s** was able to induce a G2/M phase arrest and subsequent apoptosis in SGC-7901 cells.

#### 2.2.5 Induction of cell apoptosis

**FIGURE 7:**
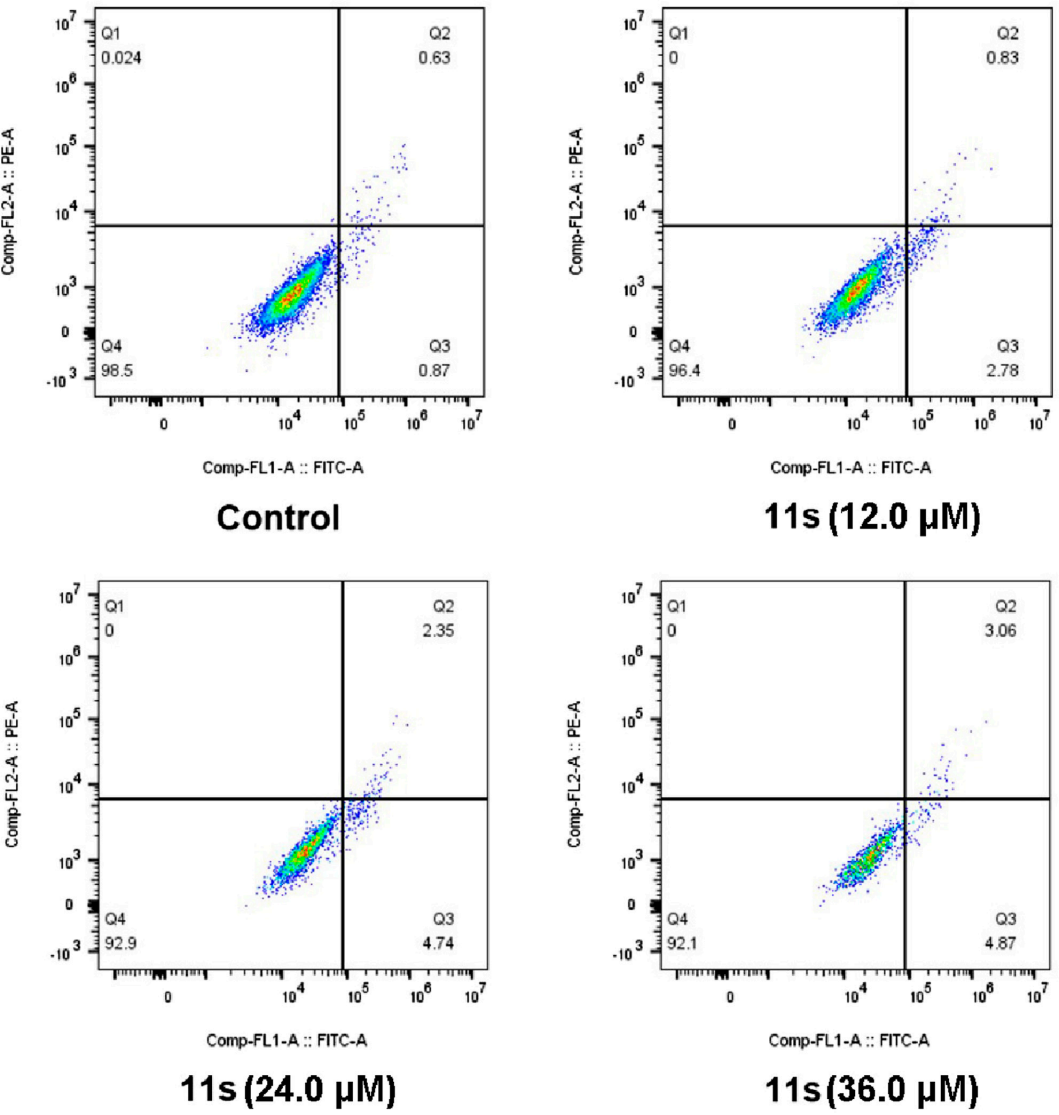
Analyses of apoptosis induction in SGC-7901 cells. Cells were harvested and stained with Annexin-V/PI for analysis after treatment with different concentrations of compound **11s** and control for 48 h. The diverse cell stages were given as live (Q4), early apoptotic (Q3), late apoptotic (Q2), and necrotic cells (Q1).

Annexin VFITC/PI assay was performed to investigate whether compound **11s** induced apoptosis. The total percentage of early (Annexin-V+/PI-) and late (Annexin-V+/PI-) apoptotic cells was only 1.50% in the control group after 48 h of treatment, as shown in Figure 7. However, after 48 h of treatment with 12.0 µM of **11s**, 3.61% of the total number of apoptotic cells was obtained. In addition, the percentage of apoptotic cells increased to 7.09% and 7.93% when SGC-7901 cells were incubated with higher concentrations of **11s** at 24.0 µM and 36.0 µM. These results revealed that **11s** was an effective, concentration-dependent inducer of apoptosis in SGC-7901 cells.

#### 2.2.6 Molecular docking analysis

**FIGURE 8:**
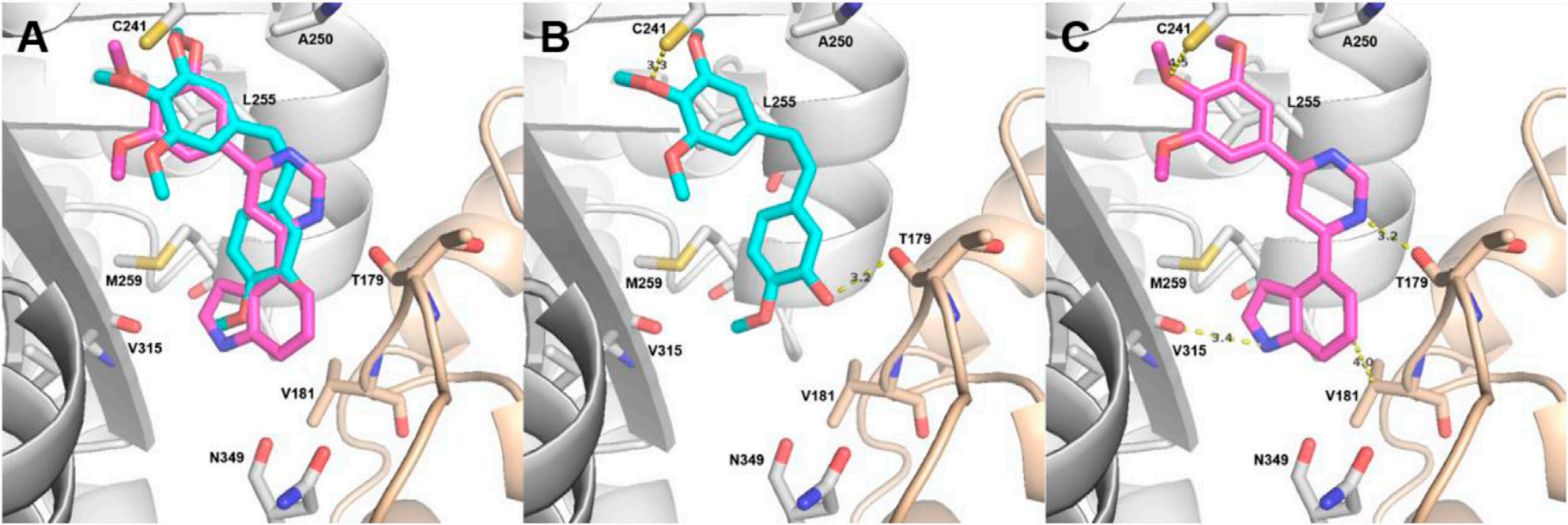
Proposed binding modes for 11s **(A, C)** in comparison with CA-4 **(A, B)** at the colchicine site. Carbon atoms are shown in cyan for CA-4 and in magenta for **11s**. The residues from the α-tubulin chain are shown in pale yellow, whereas residues from β-tubulin are colored in gray.

Molecular docking studies of CA-4 and the most potent compound, **11s**, with the tubulin crystal structure (PDB: 5LYJ) were also performed to further investigate the binding interactions. Both compounds were docked to tubulin in order to compare the binding properties of **11s** with those of CA-4. The binding orientations of **11s** (magenta) and CA-4 (cyan) overlapped well in the binding models shown in Figure 8A. As depicted in Figures 8A–C hydrogen bond is present between Cysβ241 and the oxygen of the methoxy group (A-ring) of **11s** and CA-4. Another hydrogen bond is also observed between Valβ315 with the nitrogen of the indole group (**11s**, B-ring). In addition, the nitrogen of the pyrimidine group (**11s**) forms an extra hydrogen bond with the residue Thrα179. The docking score of CA-4 (Docking score: −9.2 kcal/mol) in the 5LYJ was lower than that of **11s** (Docking score: −8.8 kcal/mol), which might be the reason why CA-4 has stronger antiproliferative activities than **11s**. These molecular docking results were in support of the biological assay data above and suggested that **11s** may be a potential microtubule destabilizer.

#### 2.2.7 Physicochemical properties

To examine the drug-like properties of diarylpyrimidine derivatives, conventional physicochemical properties of CA-4, **11j**, and **11s** were predicted using a free online website (http://www.swissadme.ch/index.php) or ChemBioDraw Ultra 14.0 software for their fit with Lipinski’s five rule. As summarized in Table 3, CA-4, **11j**, and **11s** fit well with the Lipinski’s five rule.

**TABLE 3:** Prediction of physicochemical properties of **CA-4**, **11j**, and **11s** ^a^.

Compounds	cLogP	TPSA	Natoms	MW	HBA	HBD
Standard	<5	<140		<500	<10	<5
**CA-4**	3.32	57.15	43	316.35	4	1
**11j**	2.73	81.87	47	368.39	6	1
**11s**	3.32	64.44	46	361.40	5	1

^a^ cLogP: calculated logarithm of the octanol-water partition coefficient; TPSA: topological polar surface area; Natoms: No. of atoms; MW: molecular weight; HBA: hydrogen-bond acceptor atoms. HBD: hydrogen-bond donor atoms.

## 3 Conclusion

Overall, based on the analysis of the X-ray crystal structure of tubulin in complex with DMAM-CA-4, we reported the design and discovery of a new set of diarylpyrimidine derivatives on the cis-orientation of the olefin bond of CA-4 as the novel microtubule destabilizers with improved structural stability.

The MTT assay results proved that target compounds 11a-t exhibited moderate antiproliferative potencies against three human tumor cells (SGC-7901, HeLa, and MCF-7) *in vitro*. Studies on the mechanism of action of the most promising compound showed that **11s** inhibits tubulin polymerization, disrupts the microtubule network of the SGC-7901 cells and arrests the cell cycle in G2/M phase, subsequently inducing apoptosis. **11s** was highly selective for cancer cells compared to non-tumour HSF cells. Molecular docking analysis revealed that the most biologically active compound **11s** binds very favourably to the colchicine site and provided a structural explanation for the SAR. Lastly, the prediction of physicochemical properties indicated that **11s** fits well with five Lipinski’s rule. This work provides a new perspective for the discovery of new microtubule destabilizers.

## 4 Experimental

### 4.1 Chemistry

#### 4.1.1 Materials and methods

All solvents and reagents were purchased from reputable chemical companies, ensuring the highest quality for our experiments. The ^1^H NMR and ^13^C NMR spectra were conducted in CDCl_3_ using TMS as an internal reference on a Bruker AVANCE spectrometer, operating at frequencies of 500 MHz for proton nuclei and 126 MHz for carbon nuclei. This advanced instrumentation allowed us to obtain precise structural information about the compounds under investigation. For accurate determination of molecular weights and elemental compositions, high resolution mass spectra (HRMS) were recorded using an Agilent Accurate-Mass Q-TOF 6530 instrument in electrospray ionization (ESI) mode. This technology provided us with highly accurate mass measurements, enabling confident identification of the analyzed compounds. To efficiently carry out our reactions, microwave synthesis was employed utilizing a single mode cavity microwave synthesizer manufactured by CEM Corporation based in North Carolina, United States. This innovative equipment allowed us to rapidly heat reaction mixtures under controlled conditions, resulting in improved yields and reduced reaction times compared to conventional heating methods. In order to monitor the progress of our reactions visually, thin-layer chromatography (TLC) analysis was performed under both UV light wavelengths: 365 nm and 254 nm. By comparing the migration distances of reactants and products on TLC plates coated with appropriate stationary phases, we could assess the extent of conversion during each step of our synthetic procedures.

#### 4.1.2 General synthetic procedure for 4,6-dichloropyrimidine (13)

Triethylamine (4 mmol) and phosphorus oxychloride (15.8 mmol) were mixed dropwise in a 50 mL round-bottomed flask. Then 4,6-dihydroxypyrimidine (2.3 mmol) was added dropwise. The reaction was refluxed for 1 h and then poured onto crushed ice. The precipitate was filtered and then purified by flash chromatography eluted with hexanes/ethyl acetate (1:1) to give the title compound as a light yellow solid.

#### 4.1.3 General synthetic procedure for 4-chloro-6-(3,4,5-trimethoxyphenyl)pyrimidine (14)

To the mixture of THF and water, 3,4,5-trimethoxyphenyl boric acid (1 mmol) was added under argon protection. 4,6-dichloropyrimidine (1 mmol), Pd(PPh_3_)_4_ (0.05 mmol), and K_2_CO_3_ (2 mmol) were added and reacted for 20 min at 110°C in a microwave reactor. As soon as the TLC monitoring reaction was finished, the system cooled, the solvent was removed, and water was added. The ethyl acetate extraction was then combined with the organic phase, washed the organic phase with saturated brine, then dried anhydrous Na_2_SO_4_, filtered, vacuum evaporated, column chromatography, and obtained the target compound.

#### 4.1.4 General synthetic procedure for diarylpyrimidine derivatives (**11a-t**)

A mixture of Pd(PPh_3_)_4_ (0.010 mmol), 4-chloro-6-(3,4,5-trimethoxyphenyl) pyrimidine (**14**, 0.15 mmol), K_2_CO_3_ (0.60 mmol), and the corresponding substituted phenylboronic acid (0.16 mmol) in a solution of 1,4-dioxane/H_2_O (8 mL, 3:1) was subjected to degassing and purging with N_2_ for three cycles. Subsequently, the reaction mixture was stirred under an N_2_ atmosphere at 126°C for 25 min in a microwave reactor until completion as indicated by TLC analysis. Upon completion, H_2_O (50 mL) was added to the reaction mixture followed by extraction with ethyl acetate. The combined organic layers were then washed with brine solution and dried over anhydrous Na_2_SO_4_ before filtration and concentration under vacuum to yield a residue that underwent purification via column chromatography using a *n*-hexane/ethyl acetate eluent mixture in a ratio of 4:1 to afford the desired diarylpyrimidine derivatives (**11a-t**).

##### 4.1.4.1 4-Phenyl-6-(3,4,5-trimethoxyphenyl)pyrimidine (**11a**)

White solid; yield: 65%; Mp: 88°C–89°C; ^1^H NMR (500 MHz, CDCl_3_) δ 9.29 (d, *J* = 1.3 Hz, 1H), 8.14 (dd, *J* = 6.5, 3.3 Hz, 2H), 8.02 (d, *J* = 1.3 Hz, 1H), 7.55 (dd, *J* = 5.1, 1.9 Hz, 3H), 7.40 (s, 2H), 4.00 (s, 6H), 3.94 (s, 3H); ^13^C NMR (126 MHz, CDCl_3_) δ 164.73, 164.24, 159.04, 153.70 (2C), 140.78, 137.05, 132.39, 130.94, 129.02 (2C), 127.19 (2C), 112.52, 104.51 (2C), 61.00, 56.39 (2C); HRMS calcd for C_19_H_19_N_2_O_3_ [M + H]^+^ 323.1396, found 323.1390.

##### 4.1.4.2 4-(o-tolyl)-6-(3,4,5-trimethoxyphenyl)pyrimidine (**11b**)

White solid; yield: 82%; Mp: 93°C–95°C; ^1^H NMR (500 MHz, CDCl_3_) δ 9.30 (d, *J* = 1.3 Hz, 1H), 7.75 (d, *J* = 1.3 Hz, 1H), 7.51 - 7.47 (m, 1H), 7.44 - 7.36 (m, 3H), 7.34 (dd, *J* = 9.0, 4.7 Hz, 2H), 3.97 (s, 6H), 3.93 (s, 3H), 2.46 (s, 3H); ^13^C NMR (126 MHz, CDCl_3_) δ 167.95, 163.57, 158.52, 153.70 (2C), 140.78, 138.21, 136.07, 132.12, 131.15, 129.52, 129.38, 126.17, 116.53, 104.41 (2C), 60.99, 56.33 (2C), 20.29; HRMS calcd for C_20_H_21_N_2_O_3_ [M + H]^+^ 337.1552, found 337.1541.

##### 4.1.4.3 4-(m-tolyl)-6-(3,4,5-trimethoxyphenyl)pyrimidine (**11c**)

White solid; yield: 56%; Mp: 80°C–82°C; ^1^H NMR (500 MHz, CDCl_3_) δ 9.28 (d, *J* = 0.8 Hz, 1H), 8.00 (d, *J* = 1.0 Hz, 1H), 7.97 (s, 1H), 7.91 (d, *J* = 7.7 Hz, 1H), 7.43 (t, *J* = 7.6 Hz, 1H), 7.40 (s, 2H), 7.35 (d, *J* = 7.5 Hz, 1H), 4.00 (s, 6H), 3.94 (s, 3H), 2.48 (s, 3H); ^13^C NMR (126 MHz, CDCl_3_) δ 164.90, 164.19, 158.98, 153.69 (2C), 140.77, 138.84, 137.01, 132.43, 131.72, 128.90, 127.82, 124.30, 112.59, 104.55 (2C), 60.99, 56.41 (2C), 21.50; HRMS calcd for C_20_H_21_N_2_O_3_ [M + H]^+^ 337.1552, found 337.1540.

##### 4.1.4.4 4-(p-tolyl)-6-(3,4,5-trimethoxyphenyl)pyrimidine (**11d**)

White solid; yield: 91%; Mp: 133°C–134°C; ^1^H NMR (500 MHz, CDCl_3_) δ 9.26 (d, *J* = 1.3 Hz, 1H), 8.05 (d, *J* = 8.2 Hz, 2H), 7.99 (d, *J* = 1.3 Hz, 1H), 7.39 (s, 2H), 7.35 (d, *J* = 7.9 Hz, 2H), 4.00 (s, 6H), 3.94 (s, 3H), 2.45 (s, 3H); ^13^C NMR (126 MHz, CDCl_3_) δ 164.64, 164.10, 158.99, 153.67 (2C), 141.39, 140.71, 134.20, 132.52, 129.75 (2C), 127.08 (2C), 112.14, 104.50 (2C), 60.99, 56.39 (2C), 21.46; HRMS calcd for C_20_H_21_N_2_O_3_ [M + H]^+^ 337.1552, found 337.1576.

##### 4.1.4.5 4-(3,4-dimethylphenyl)-6-(3,4,5-trimethoxyphenyl)pyrimidine (**11e**)

White solid; yield: 58%; Mp: 93°C–95°C; ^1^H NMR (500 MHz, CDCl_3_) δ 9.26 (d, *J* = 1.2 Hz, 1H), 7.98 (d, *J* = 1.2 Hz, 1H), 7.94 (s, 1H), 7.85 (dd, *J* = 7.8, 1.6 Hz, 1H), 7.39 (s, 2H), 7.30 (d, *J* = 7.9 Hz, 1H), 4.00 (s, 6H), 3.94 (s, 3H), 2.39 (s, 3H), 2.35 (s, 3H); ^13^C NMR (126 MHz, CDCl_3_) δ 164.80, 164.06, 158.94, 153.67 (2C), 140.70, 140.11, 137.42, 134.55, 132.57, 130.28, 128.24, 124.58, 112.21, 104.54 (2C), 60.99, 56.41 (2C), 19.90, 19.82; HRMS calcd for C_21_H_23_N_2_O_3_ [M + H]^+^ 351.1709, found 351.1708.

##### 4.1.4.6 4-(2-methoxyphenyl)-6-(3,4,5-trimethoxyphenyl)pyrimidine (**11f**)

White solid; yield: 70%; Mp: 104°C–106°C; ^1^H NMR (500 MHz, CDCl_3_) δ 9.29 (d, *J* = 1.3 Hz, 1H), 8.26 (d, *J* = 1.3 Hz, 1H), 7.99 (dd, *J* = 7.7, 1.8 Hz, 1H), 7.47 (ddd, *J* = 8.3, 7.4, 1.8 Hz, 1H), 7.37 (s, 2H), 7.13 (td, *J* = 7.6, 1.0 Hz, 1H), 7.06 (d, *J* = 8.9 Hz, 1H), 3.98 (s, 6H), 3.93 (s, 3H), 3.92 (s, 3H); ^13^C NMR (126 MHz, CDCl_3_) δ 163.48, 163.15, 158.70, 157.72, 153.62 (2C), 140.52, 132.78, 131.69, 131.00, 126.49, 121.26, 117.45, 111.61, 104.55 (2C), 60.98, 56.30 (2C), 55.73; HRMS calcd for C_20_H_21_N_2_O_4_ [M + H]^+^ 353.1501, found 353.1499.

##### 4.1.4.7 4-(3-methoxyphenyl)-6-(3,4,5-trimethoxyphenyl)pyrimidine (**11g**)

Light yellow solid; yield: 53%; Mp: 121°C–122°C; ^1^H NMR (500 MHz, CDCl_3_) δ 9.28 (d, *J* = 1.2 Hz, 1H), 8.00 (d, *J* = 1.2 Hz, 1H), 7.73 (dd, *J* = 2.3, 1.8 Hz, 1H), 7.71 - 7.66 (m, 1H), 7.45 (t, *J* = 8.0 Hz, 1H), 7.39 (s, 2H), 7.08 (dd, *J* = 8.2, 2.6 Hz, 1H), 4.00 (s, 6H), 3.94 (s, 3H), 3.92 (s, 3H); ^13^C NMR (126 MHz, CDCl_3_) δ 164.50, 164.25, 160.22, 158.97, 153.70 (2C), 140.79, 138.50, 132.34, 130.02, 119.50, 116.76, 112.64, 112.49, 104.51 (2C), 60.99, 56.40 (2C), 55.49; HRMS calcd for C_20_H_21_N_2_O_4_ [M + H]^+^ 353.1501, found 353.1502.

##### 4.1.4.8 4-(4-methoxyphenyl)-6-(3,4,5-trimethoxyphenyl)pyrimidine (**11h**)

Light yellow solid; yield: 78%; Mp: 99°C–101°C; ^1^H NMR (500 MHz, CDCl_3_) δ 9.23 (s, 1H), 8.13 (d, *J* = 8.9 Hz, 2H), 7.95 (d, *J* = 1.1 Hz, 1H), 7.38 (s, 2H), 7.05 (d, *J* = 8.9 Hz, 2H), 4.00 (s, 6H), 3.94 (s, 3H), 3.90 (s, 3H); ^13^C NMR (126 MHz, CDCl_3_) δ 164.15, 163.96, 162.08, 158.90, 153.67 (2C), 140.68, 132.56, 129.34, 128.73 (2C), 114.38 (2C), 111.59, 104.49 (2C), 60.99, 56.39 (2C), 55.45; HRMS calcd for C_20_H_21_N_2_O_4_ [M + H]^+^ 353.1501, found 353.1525.

##### 4.1.4.9 4-(4-ethoxyphenyl)-6-(3,4,5-trimethoxyphenyl)pyrimidine (**11i**)

Light yellow solid; yield: 84%; Mp: 149°C–151°C; ^1^H NMR (500 MHz, CDCl_3_) δ 9.22 (d, *J* = 1.2 Hz, 1H), 8.11 (d, *J* = 8.9 Hz, 2H), 7.94 (d, *J* = 1.1 Hz, 1H), 7.37 (s, 2H), 7.03 (d, *J* = 8.9 Hz, 2H), 4.12 (q, *J* = 7.0 Hz, 2H), 3.99 (s, 6H), 3.93 (s, 3H), 1.46 (t, J = 7.0 Hz, 3H); ^13^C NMR (126 MHz, CDCl_3_) δ 164.18, 163.93, 161.47, 158.92, 153.65 (2C), 140.64, 132.60, 129.14, 128.70 (2C), 114.85 (2C), 111.54, 104.47 (2C), 63.68, 60.98, 56.38 (2C), 14.74; HRMS calcd or C_21_H_23_N_2_O_4_ [M + H]^+^ 367.1658, found 367.1650.

##### 4.1.4.10 2-Methoxy-5-(6-(3,4,5-trimethoxyphenyl)pyrimidin-4-yl)phenol (**11j**)

Yellow solid; yield: 79%; Mp: 117°C–118°C; ^1^H NMR (500 MHz, CDCl_3_) δ 9.22 (d, *J* = 1.1 Hz, 1H), 7.92 (d, *J* = 1.1 Hz, 1H), 7.75 (dt, *J* = 5.8, 2.9 Hz, 1H), 7.72 (dd, *J* = 10.4, 1.7 Hz, 1H), 7.37 (s, 2H), 6.98 (d, *J* = 8.4 Hz, 1H), 6.28 (s, 1H), 3.98 (s, 6H), 3.95 (s, 3H), 3.93 (s, 3H); ^13^C NMR (126 MHz, CDCl_3_) δ 164.11, 163.94, 158.86, 153.65 (2C), 149.24, 146.13, 140.61, 132.53, 130.16, 119.69, 113.29, 111.76, 110.83, 104.40 (2C), 60.98, 56.36 (2C), 56.02; HRMS calcd for C_20_H_21_N_2_O_5_ [M + H]^+^ 369.1450, found 369.1459.

##### 4.1.4.11 4-(3,4-dimethoxyphenyl)-6-(3,4,5-trimethoxyphenyl)pyrimidine (**11k**)

Light yellow solid; yield: 54%; Mp: 127°C–128°C; ^1^H NMR (500 MHz, CDCl_3_) δ 9.24 (d, *J* = 1.2 Hz, 1H), 7.96 (d, *J* = 1.2 Hz, 1H), 7.82 (d, *J* = 2.0 Hz, 1H), 7.69 (dd, *J* = 8.4, 2.1 Hz, 1H), 7.38 (s, 2H), 7.00 (d, *J* = 8.4 Hz, 1H), 4.02 (s, 3H), 3.99 (s, 6H), 3.97 (s, 3H), 3.93 (s, 3H); ^13^C NMR (126 MHz, CDCl_3_) δ 164.13, 164.01, 158.89, 153.67 (2C), 151.67, 149.51, 140.72, 132.57, 129.72, 120.17, 111.77, 111.03, 110.05, 104.56 (2C), 60.99, 56.42 (2C), 56.12, 56.04; HRMS calcd for C_21_H_23_N_2_O_5_ [M + H]^+^ 383.1607, found 383.1603.

##### 4.1.4.12 4-(4-nitrophenyl)-6-(3,4,5-trimethoxyphenyl)pyrimidine (**11l**)

Yellow solid; yield: 50%; Mp: 156°C–158°C; ^1^H NMR (500 MHz, CDCl_3_) δ 9.35 (d, *J* = 1.3 Hz, 1H), 8.41 (d, *J* = 9.0 Hz, 2H), 8.33 (d, *J* = 9.0 Hz, 2H), 8.07 (d, *J* = 1.3 Hz, 1H), 7.42 (s, 2H), 4.01 (s, 6H), 3.95 (s, 3H); ^13^C NMR (126 MHz, CDCl_3_) δ 164.96, 162.25, 159.25, 153.80 (2C), 149.31, 142.91, 131.74, 128.43, 128.21 (2C), 124.17 (2C), 113.02, 104.67 (2C), 61.03, 56.46 (2C); HRMS calcd for C_19_H_18_N_3_O_5_ [M + H]^+^ 368.1246, found 368.1253.

##### 4.1.4.13 4-(6-(3,4,5-trimethoxyphenyl)pyrimidin-4-yl)phenol (**11m**)

Light yellow solid; yield: 51%; Mp: 224°C–226°C; ^1^H NMR (500 MHz, CDCl_3_) δ 9.23 (d, *J* = 1.3 Hz, 1H), 8.09 (d, *J* = 8.8 Hz, 2H), 7.95 (d, *J* = 1.3 Hz, 1H), 7.38 (s, 2H), 6.99 (d, *J* = 8.8 Hz, 2H), 5.52 (s, 1H), 4.00 (s, 6H), 3.94 (s, 3H); ^13^C NMR (126 MHz, CDCl_3_) δ 164.12, 164.03, 158.91, 158.37, 153.67 (2C), 140.69, 132.54, 130.00, 129.01 (2C), 115.94 (2C), 111.62, 104.50 (2C), 61.00, 56.40 (2C); HRMS calcd for C_19_H_19_N_2_O_4_ [M + H]^+^ 339.1345, found 339.1368.

##### 4.1.4.14 4-(4-fluorophenyl)-6-(3,4,5-trimethoxyphenyl)pyrimidine (**11n**)

White solid; yield: 91%; Mp: 133°C–135°C; ^1^H NMR (500 MHz, CDCl_3_) δ 9.25 (s, 1H), 8.15 (dd, *J* = 8.6, 5.4 Hz, 2H), 7.96 (s, 1H), 7.38 (s, 2H), 7.21 (t, *J* = 8.6 Hz, 2H), 3.99 (s, 6H), 3.93 (s, 3H); ^13^C NMR (126 MHz, CDCl_3_) δ 164.63, 164.30, 163.54, 159.00, 153.69 (2C), 140.85, 133.15, 132.25, 129.25 (2C), 116.06 (2C), 112.05, 104.51 (2C), 60.99, 56.38 (2C); HRMS calcd for C_19_H_18_FN_2_O_3_ [M + H]^+^ 341.1301, found 341.1302.

##### 4.1.4.15 4-(4-chlorophenyl)-6-(3,4,5-trimethoxyphenyl)pyrimidine (**11o**)

Yellow solid; yield: 55%; Mp: 136°C–137°C; ^1^H NMR (500 MHz, CDCl_3_) δ 9.28 (d, *J* = 1.3 Hz, 1H), 8.10 (d, *J* = 8.6 Hz, 2H), 7.98 (d, *J* = 1.3 Hz, 1H), 7.52 (d, *J* = 8.7 Hz, 2H), 7.39 (s, 2H), 4.00 (s, 6H), 3.94 (s, 3H); ^13^C NMR (126 MHz, CDCl_3_) δ 164.45, 163.46, 159.07, 153.72 (2C), 140.92, 137.23, 135.45, 132.19, 129.26 (2C), 128.48 (2C), 112.18, 104.56 (2C), 61.00, 56.42 (2C); HRMS calcd for C_19_H_18_ClN_2_O_3_ [M + H]^+^ 357.1006, found 357.1010.

##### 4.1.4.16 4-(thiophen-3-yl)-6-(3,4,5-trimethoxyphenyl)pyrimidine (**11p**)

Light yellow solid; yield: 87%; Mp: 106°C–108°C; ^1^H NMR (500 MHz, CDCl_3_) δ 9.20 (d, *J* = 1.3 Hz, 1H), 8.19 (dd, *J* = 3.0, 1.3 Hz, 1H), 7.85 (d, *J* = 1.3 Hz, 1H), 7.76 (dd, *J* = 5.1, 1.2 Hz, 1H), 7.45 (dd, *J* = 5.1, 3.0 Hz, 1H), 7.36 (s, 2H), 3.98 (s, 6H), 3.93 (s, 3H); ^13^C NMR (126 MHz, CDCl_3_) δ 164.20, 160.24, 159.06, 153.66 (2C), 140.77, 140.10, 132.33, 127.02, 126.85, 125.93, 112.15, 104.48 (2C), 60.98, 56.38 (2C); HRMS calcd for C_17_H_17_N_2_O_3_S [M + H]^+^ 329.0960, found 329.0955.

##### 4.1.4.17 4-(pyridin-3-yl)-6-(3,4,5-trimethoxyphenyl)pyrimidine (**11q**)

Light yellow solid; yield: 97%; Mp: 120°C–121°C; ^1^H NMR (500 MHz, CDCl_3_) δ 9.33 (s, 1H), 9.30 (d, *J* = 1.2 Hz, 1H), 8.76 (d, *J* = 4.0 Hz, 1H), 8.46 (d, *J* = 8.0 Hz, 1H), 8.04 (d, *J* = 1.2 Hz, 1H), 7.48 (dd, *J* = 7.9, 4.7 Hz, 1H), 7.40 (s, 2H), 3.99 (s, 6H), 3.93 (s, 3H); ^13^C NMR (126 MHz, CDCl_3_) δ 164.56, 162.27, 159.24, 153.73 (2C), 151.65, 148.44, 141.03, 134.75, 132.79, 131.88, 123.83, 112.44, 104.52 (2C), 61.00, 56.39 (2C); HRMS calcd for C_18_H_18_N_3_O_3_ [M + H]^+^ 324.1348, found 324.1340.

##### 4.1.4.18 4-(pyridin-4-yl)-6-(3,4,5-trimethoxyphenyl)pyrimidine (**11r**)

Light yellow solid; yield: 67%; Mp: 124°C–126°C; ^1^H NMR (500 MHz, CDCl_3_) δ 9.34 (d, *J* = 1.3 Hz, 1H), 8.83 (d, *J* = 5.7 Hz, 2H), 8.06 (d, *J* = 1.3 Hz, 1H), 8.01 (dd, *J* = 4.5, 1.6 Hz, 2H), 7.41 (s, 2H), 4.00 (s, 6H), 3.94 (s, 3H); ^13^C NMR (126 MHz, CDCl_3_) δ 164.96, 162.27, 159.32, 153.78 (2C), 150.77 (2C), 144.33, 141.19, 131.76, 121.09 (2C), 112.78, 104.62 (2C), 61.02, 56.43 (2C); HRMS calcd for C_18_H_18_N_3_O_3_ [M + H]^+^ 324.1348, found 324.1346.

##### 4.1.4.19 4-(6-(3,4,5-trimethoxyphenyl)pyrimidin-4-yl)-1H-indole (**11s**)

Light yellow solid; yield: 84%; Mp: 84°C–86°C; ^1^H NMR (500 MHz, CDCl_3_) δ 9.38 (d, *J* = 1.3 Hz, 1H), 8.97 (s, 1H), 8.15 (d, *J* = 1.3 Hz, 1H), 7.73 (dd, *J* = 7.4, 0.8 Hz, 1H), 7.53 (d, *J* = 8.1 Hz, 1H), 7.42 (s, 2H), 7.36 - 7.31 (m, 2H), 7.15 (s, 1H), 3.98 (s, 6H), 3.94 (s, 3H); ^13^C NMR (126 MHz, CDCl_3_) δ 166.59, 163.71, 158.89, 153.67 (2C), 140.56, 136.85, 132.69, 129.69, 126.05, 126.02, 121.93, 120.51, 114.93, 113.73, 104.50 (2C), 102.39, 61.00, 56.35 (2C); HRMS calcd for C_21_H_20_N_3_O_3_ [M + H]^+^ 362.1505, found 362.1495.

##### 4.1.4.20 4-(naphthalen-2-yl)-6-(3,4,5-trimethoxyphenyl)pyrimidine (**11t**)

Light yellow solid; yield: 86%; Mp: 135°C–137°C; ^1^H NMR (500 MHz, CDCl_3_) δ 9.33 (d, *J* = 1.2 Hz, 1H), 8.67 (s, 1H), 8.22 (dd, *J* = 8.6, 1.8 Hz, 1H), 8.14 (d, *J* = 1.2 Hz, 1H), 8.00 (dd, *J* = 8.8, 4.2 Hz, 2H), 7.90 (dd, *J* = 6.1, 3.0 Hz, 1H), 7.61 - 7.51 (m, 2H), 7.43 (s, 2H), 4.01 (s, 6H), 3.95 (s, 3H); ^13^C NMR (126 MHz, CDCl_3_) δ 164.56, 164.29, 159.06, 153.71 (2C), 140.82, 134.58, 134.23, 133.26, 132.42, 128.99, 128.83, 127.77, 127.50, 127.47, 126.72, 123.90, 112.68, 104.60 (2C), 61.01, 56.43 (2C); HRMS calcd for C_23_H_21_N_2_O_3_ [M + H]^+^ 373.1552, found 373.1557.

### 4.2 Biological evaluation

#### 4.2.1 Cell culture

All cell lines were derived from the American Type Culture Collection (ATCC, Manassas, VA) or our own laboratory. In order to maintain the viability and growth of HeLa, SGC-7901, MCF-7, and HSF cells, they were cultured in RPMI-1640 medium supplemented with 100 U/mL streptomycin, 100 U/mL penicillin, and 10% FBS. The use of antibiotics was necessary to prevent bacterial contamination while the addition of FBS provided essential nutrients for cell growth. The culture conditions were maintained at a temperature of 37°C in a humidified atmosphere containing 5% CO_2_ which mimicked physiological conditions within the body. Careful attention was paid to maintaining optimal culture conditions for each cell line throughout the duration of our experiments to ensure reliable results. This standardized culturing protocol ensures consistent conditions for growing HeLa, SGC-7901, MCF-7, and HSF cells under controlled laboratory settings. By following these guidelines and using reliable sources for obtaining cell lines like ATCC’s collection guarantees reproducibility across different experiments conducted by researchers globally.

#### 4.2.2 *In vitro* antiproliferative activity

The MTT assay was employed to determine the *in vitro* antiproliferative activity of all target compounds and CA-4 (Shi et al., 2022). Cells were seeded at a density of 2 × 10^4^/well in 96-well plates, taking into account the growth rate of the cell line. After 24 h, triplicate wells were treated with the compounds and media under investigation. Following 72 h of incubation at 37°C in a CO_2_-enriched environment (5%), the medium containing drugs was replaced with fresh medium containing a solution of MTT (5 mg/mL) for subsequent analysis. After an additional incubation period of 4 h, dimethyl sulfoxide (100 μL) was added to each well and gently vortexed until complete dissolution of purple formazan crystals occurred. The optical density values at OD_490_ were determined using a microplate reader. The resulting data were calculated and plotted as percentage viability relative to control samples. IC_50_ values represented drug concentrations that caused absorption by untreated wells in MTT assays to reach 50%.

#### 4.2.3 Effect on tubulin polymerization

A fluorescence-based tubulin polymerization assay kit (Cytoskeleton-Cat.#BK011P) was employed in this study to investigate the effects of CA-4 and compound **11s** on tubulin polymerization. The experimental procedures were conducted following the manufacturer’s protocol (Dufresne et al., 2007). To initiate the assay, tubulin was resuspended in ice-cold G-PEM buffer and then added to 96-well plates containing different concentrations of the indicated drugs or vehicle control. Thorough mixing of samples ensured proper distribution of the compounds within each well. To monitor tubulin assembly, a plate reader was utilized to measure fluorescence at regular intervals of 1 min for a total duration of 90 min at a temperature maintained at 37°C. This allowed us to observe any changes in tubulin polymerization over time induced by CA-4 and compound **11s**. After obtaining the data from these measurements, IC_50_ values were calculated using SPSS software specifically after a period of 20 min. These IC_50_ values provide valuable information about the concentration required for CA-4 and compound **11s** to inhibit half of the tubulin polymerization process.

#### 4.2.4 Analysis of immunofluorescence staining

Immunostaining was conducted to identify the presence of tubulin protein associated with microtubules after exposure to compound **11s** and CA-4 (Shi et al., 2022). SGC-7901 cells were seeded at a density of 1 × 10^4^ in a 24-well plate and incubated for 24 h. Subsequently, the cells were treated with either **11s**, CA-4, or the vehicle for a duration of 24 h. The control and treated cells were fixed using PBS containing 4% formaldehyde at −20°C for half an hour, followed by two washes with PBS. To enable permeabilization, the cells were exposed to PBS containing 0.1% (v/v) Triton X-100 for 5 min. Following this step, blocking was performed using PBS supplemented with 5% bovine serum albumin for 10 min. A solution consisting of primary α-tubulin antibody diluted at a ratio of 1/100 in PBS containing 2% bovine serum albumin was prepared. The plates were incubated overnight at a temperature of 4°C. After removing any unbound primary antibody using PBS, the cells were exposed to FITC-conjugated anti-mouse secondary antibody. A dilution of 1/1,000 was prepared for the anti-mouse secondary antibody using a solution of 2% BSA in PBS, and the cells were then incubated at a temperature of 37°C for a duration of 3 h. To eliminate any remaining unbound secondary antibody, the cells were rinsed with PBS solution. Following this step, DAPI dye was used to stain the nuclei. Finally, immunofluorescence was observed through a fluorescence microscope.

#### 4.2.5 Cell cycle analysis

The SGC-7901 cell line (8 × 10^4^ cells) was subjected to culture with specified concentrations of compound **11s** or a solution containing 0.05% DMSO for the indicated durations (Khazir et al., 2020). The cells were harvested through centrifugation, followed by PBS washing and fixation in ice-cold 70% ethanol. After another round of centrifugation, the fixed cells were resuspended in 500 mL of PBS supplemented with RNase at a concentration of 1 mg/mL. Subsequently, incubation at 37°C for 30 min was performed before staining the cells with propidium iodide at a concentration of 50 mg/mL under dark conditions at 4°C for half an hour. Flow cytometry analysis using FACS was then conducted on the samples. This experimental procedure was repeated no less than three times.

#### 4.2.6 Induction of cell apoptosis

An Annexin-Van-FITC/PI assay was conducted to assess the potential of the target compound in inducing apoptosis (Wang et al., 2024). SGC-7901 cells were cultured in 6-well plates (3 × 10^5^ cells/well) and treated with varying concentrations of compound **11s** or a control solution for 48 h. Subsequently, the cells were collected through centrifugation, rinsed with PBS, and suspended in binding buffer. Following this, a cell suspension was prepared by adding 10 μL of PI staining solution and 5 μL of Annexin V-FITC, which were then incubated at room temperature in darkness for 15 min. Finally, the samples were analyzed using a CytoFLEX flow cytometer and Flowjo 10.8 software was utilized to calculate the percentage of apoptotic cells.

### 4.3 Molecular docking analysis

The ligands used for molecular docking were generated in.sdf format using ChemBioDrawUltra 13.0 and processed with LigPrep in the Schrödinger package (Wang et al., 2024). The Tubulin crystal structure in complex with **11s** and CA-4 was obtained from the RCSB PDB Bank and processed using the Protein Preparation Wizard within the Schrödinger package. The ligands were also prepared using LigPrep Wizard in the Schrödinger package, including adding hydrogen atoms to residues and assigning bond orders. Subsequently, the OPLS3 force field was applied to minimize protein energy and remove steric hindrance. During docking, a grid box measuring 15 Å × 15 Å × 15 Å was generated around the protein’s active site. Docking procedures were carried out using Ligand Docking in the Schrödinger package, and the results were analyzed utilizing PyMOL.

## Data Availability

The original contributions presented in the study are included in the article/Supplementary Material, further inquiries can be directed to the corresponding authors.
