# Supplementary material for: Design, synthesis, and bioevaluation of diarylpyrimidine derivatives as novel microtubule destabilizers
Source: Front Chem. 2024 Jul 25;12:1447831. doi: 10.3389/fchem.2024.1447831 (PMC11306069; doi:10.3389/fchem.2024.1447831)
Supplement: Supplementary file 1 [file DataSheet1.docx]

Supplementary data

**Design, synthesis, and bioevaluation of diarylpyrimidine derivatives as novel microtubule destabilizers**

Yutao Xiu ^1,2^, Yujing Zhang ^3^, Shanbo Yang ^1,2^, Lingyu Shi ^1,2^,

Dongming Xing ^1,2,4*^, Chao Wang ^1,2*^

^1^ Cancer Institute, The Affiliated Hospital of Qingdao University, Qingdao University, School of Basic Medicine of Qingdao University, Qingdao, 266071, Shandong, China

^2^ Qingdao Cancer Institute, Qingdao University, Qingdao, 266071, Shandong, China

^3^ The Affiliated Cardiovascular Hospital of Qingdao University, Qingdao University, Qingdao, 266071, Shandong, China

^4^ School of Life Sciences, Tsinghua University, Beijing, 100084, China

^*^ Correspondence: wangchao20086925@126.com (C. Wang) and xdm_tsinghua@163.com (D. Xing).

**HRMS, ^1^H-NMR, and ^13^C-NMR** **spectra of all target compounds**

*4-phenyl-6-(3,4,5-trimethoxyphenyl)pyrimidine (****11a****)*

*
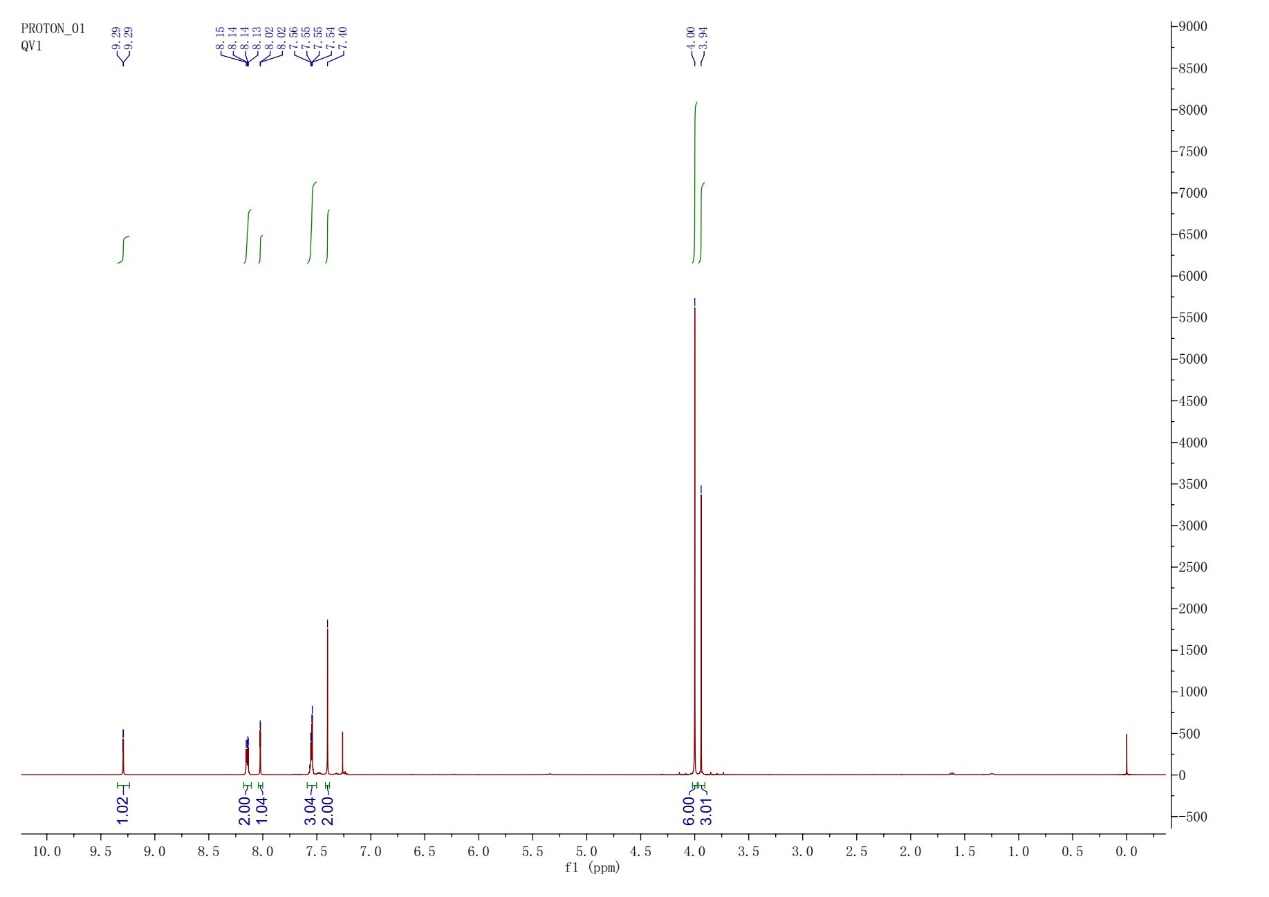
*

*
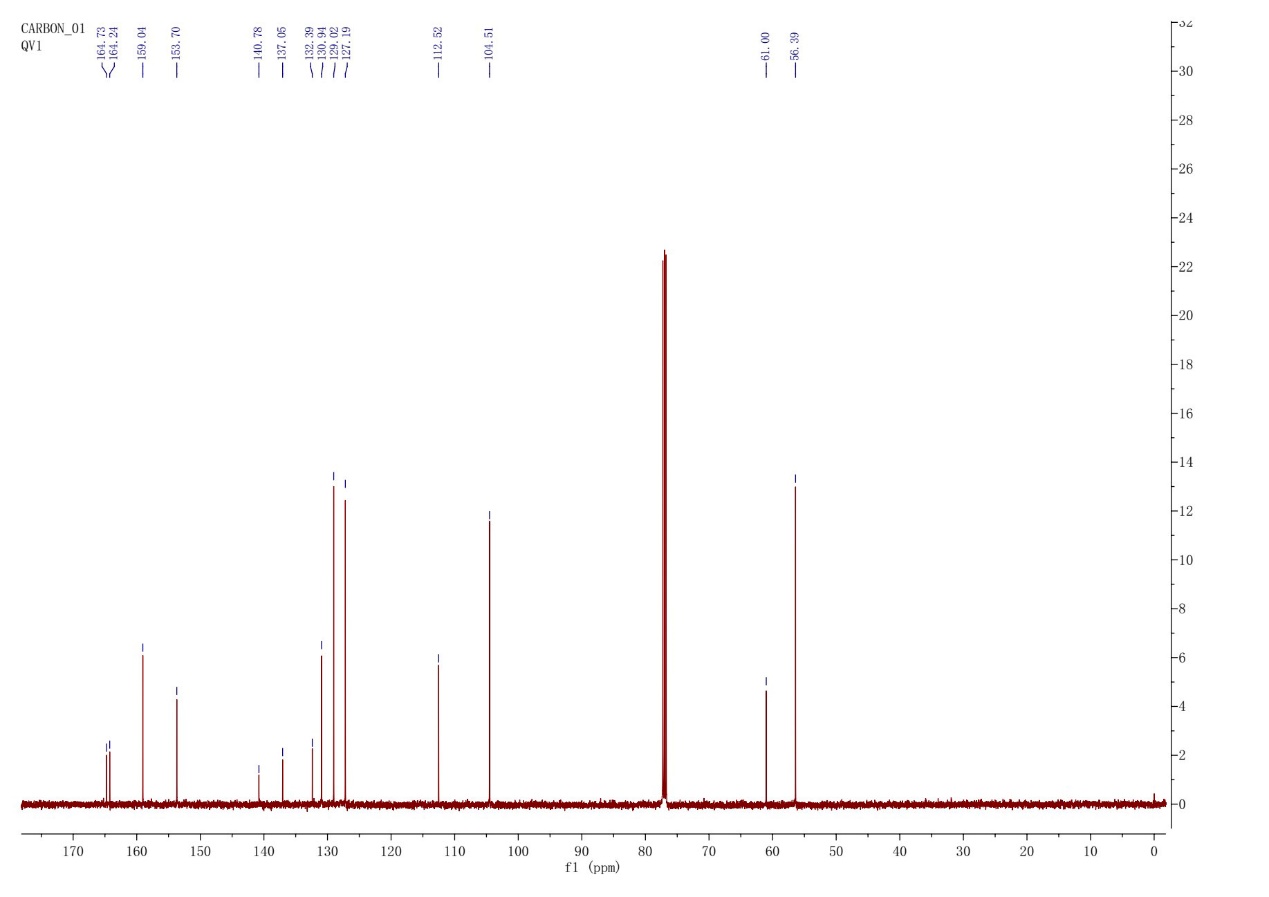
*

*4-(o-tolyl)-6-(3,4,5-trimethoxyphenyl)pyrimidine (****11b****)*

*
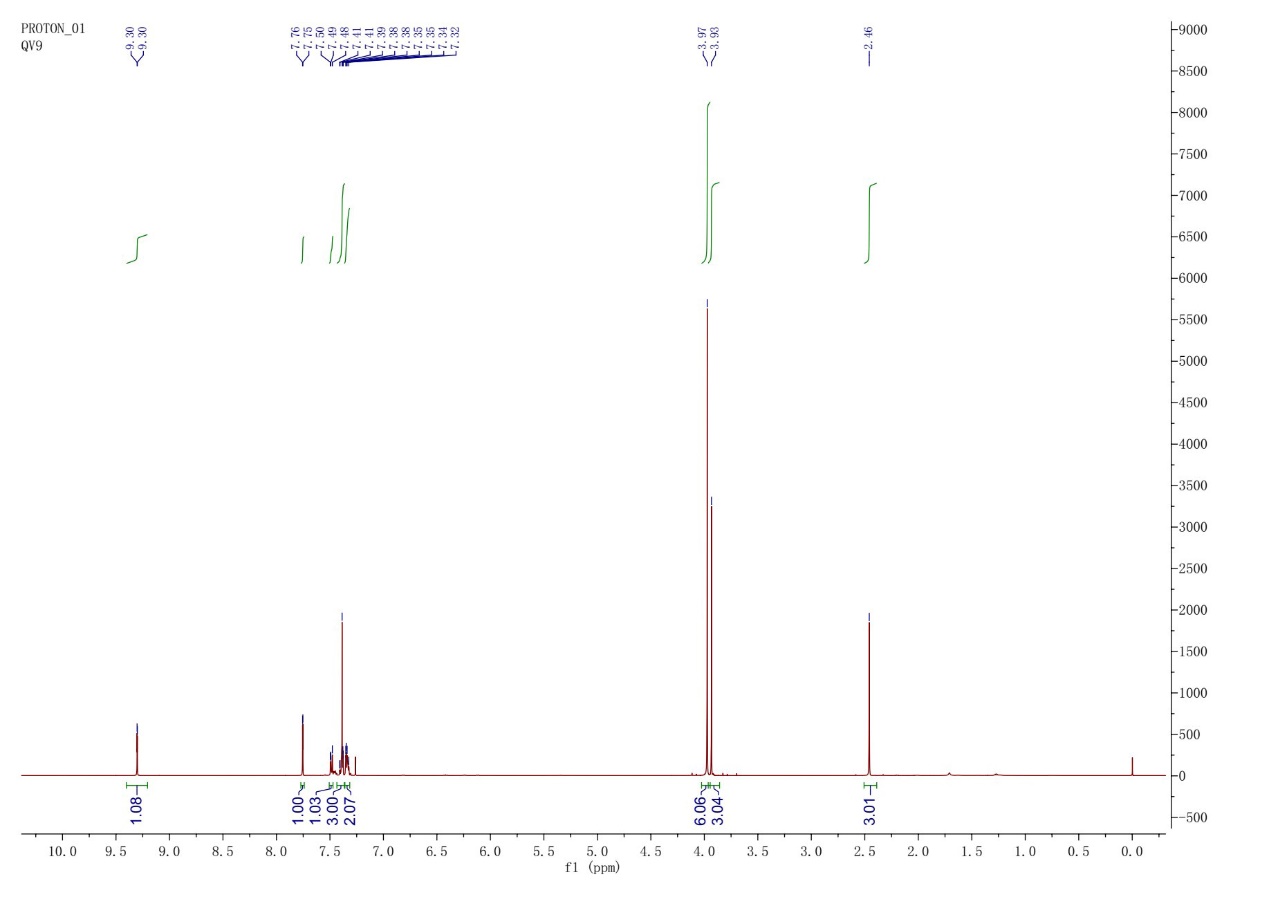
*

*
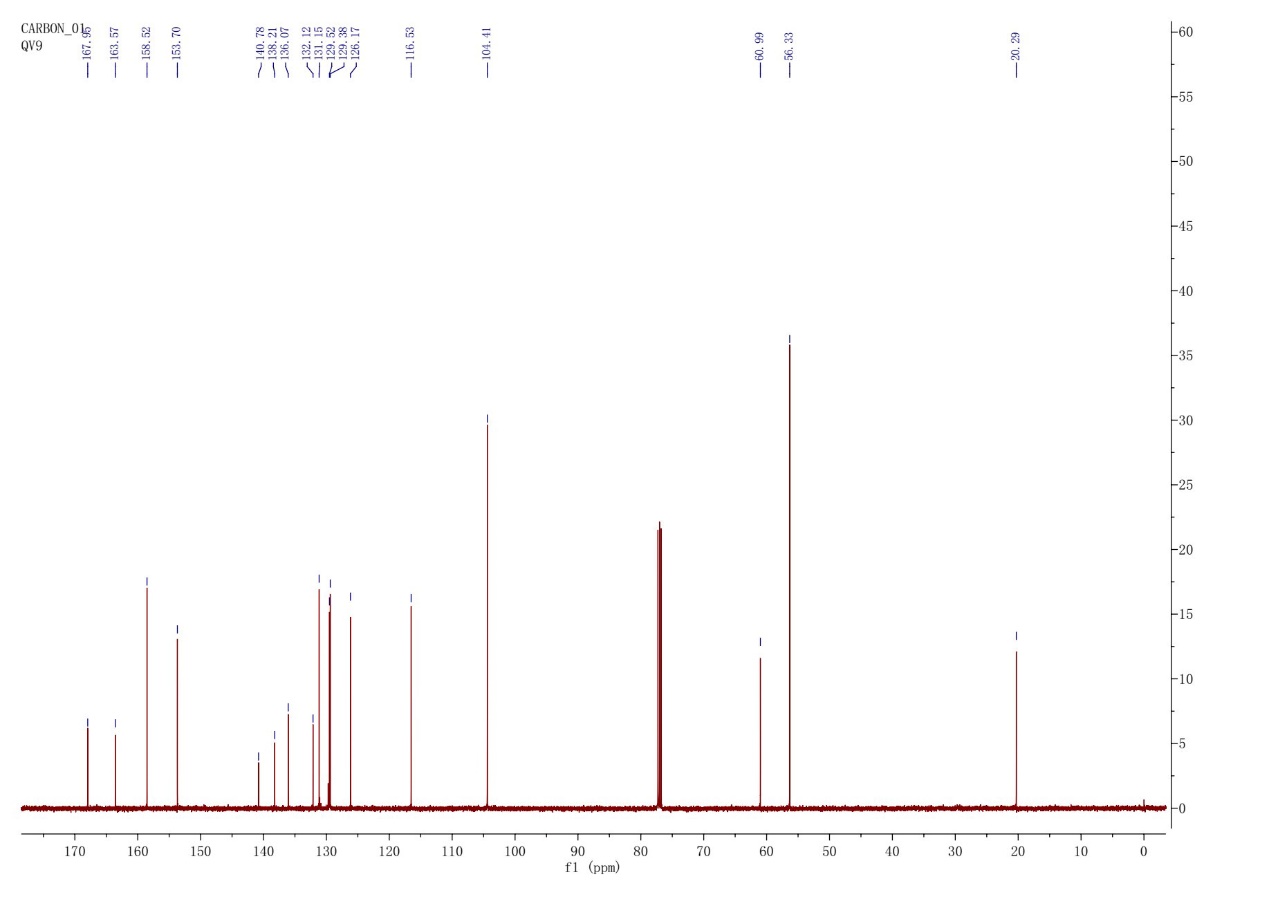
*

*4-(m-tolyl)-6-(3,4,5-trimethoxyphenyl)pyrimidine (****11c****)*

*
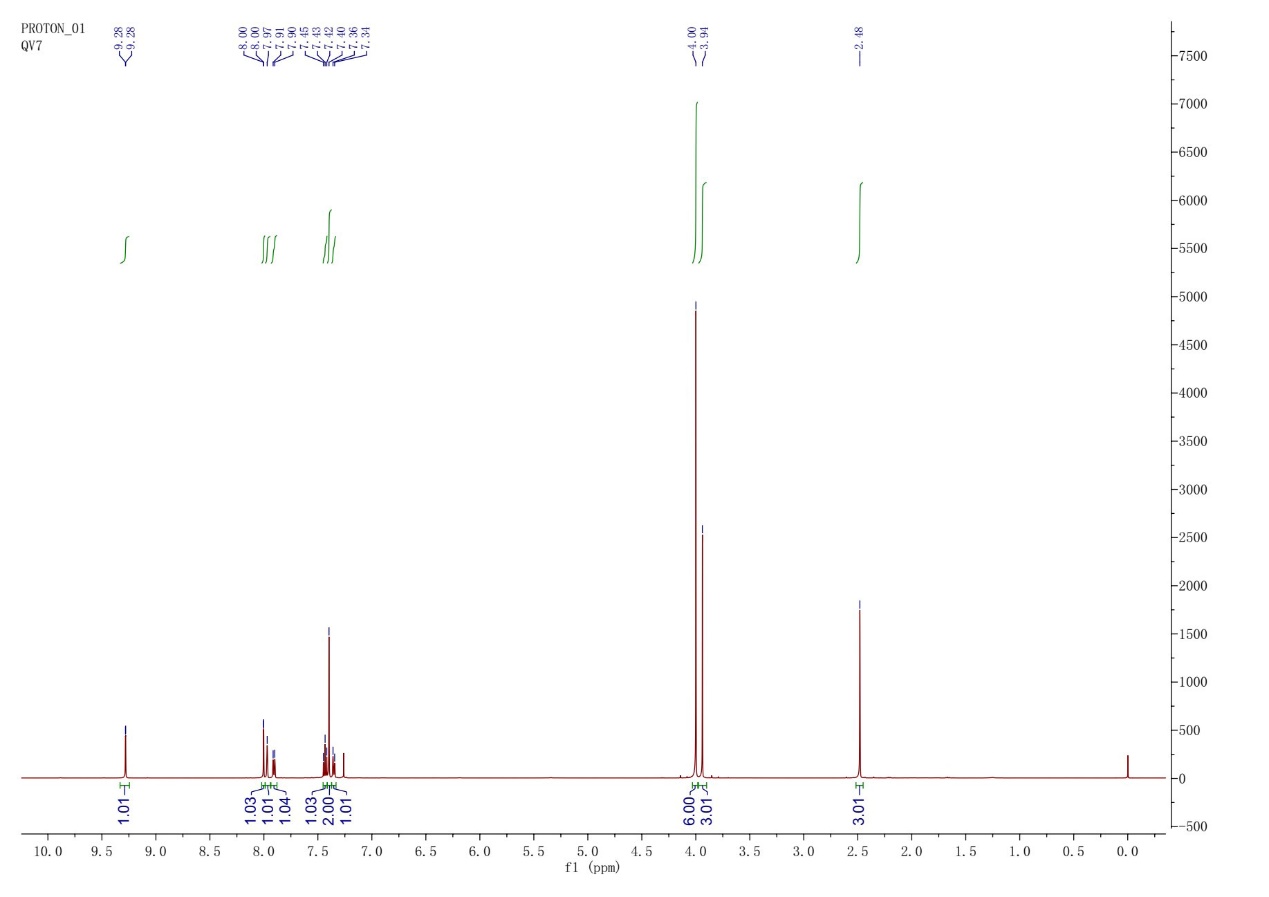
*

*
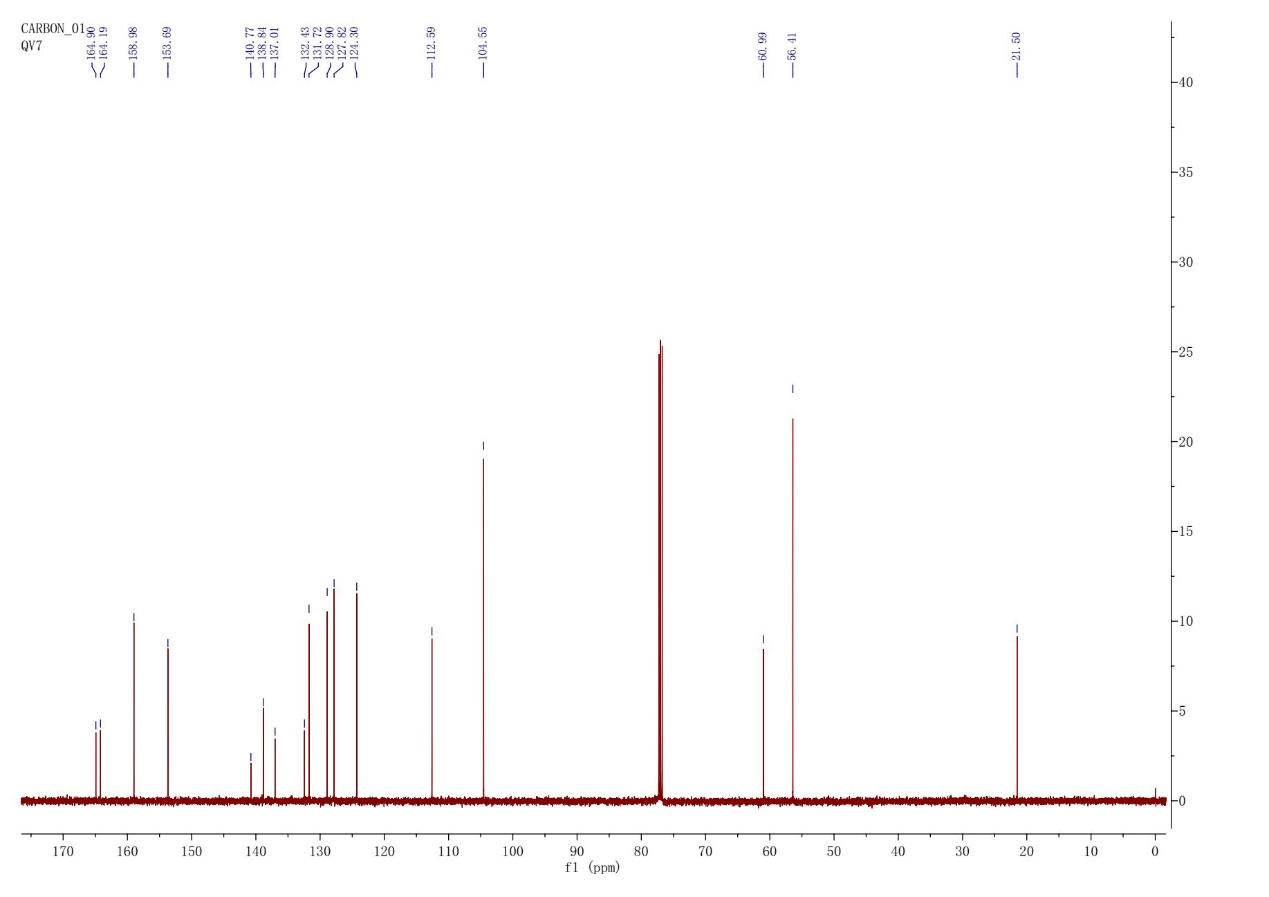
*

*4-(p-tolyl)-6-(3,4,5-trimethoxyphenyl)pyrimidine (****11d****)*

*
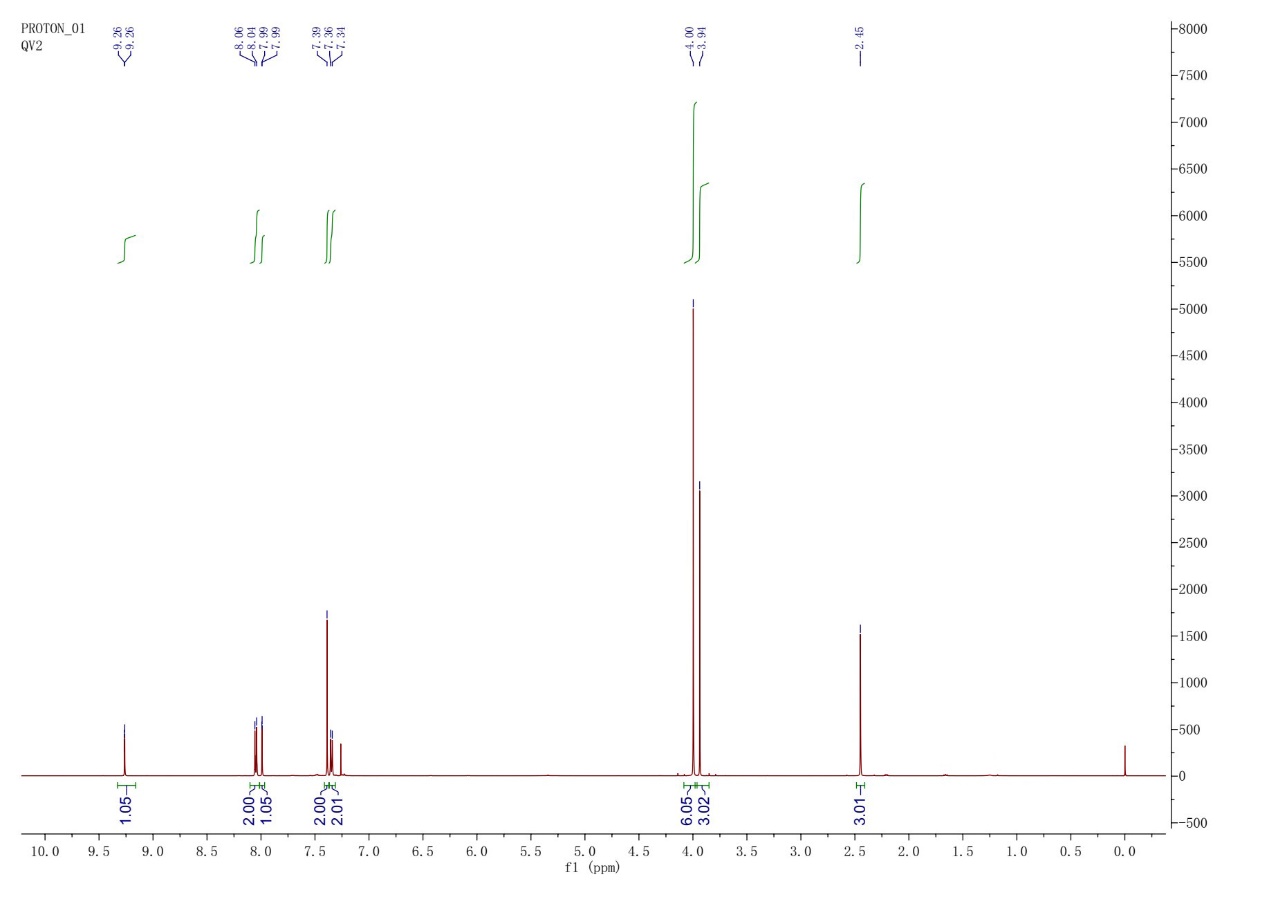
*

*
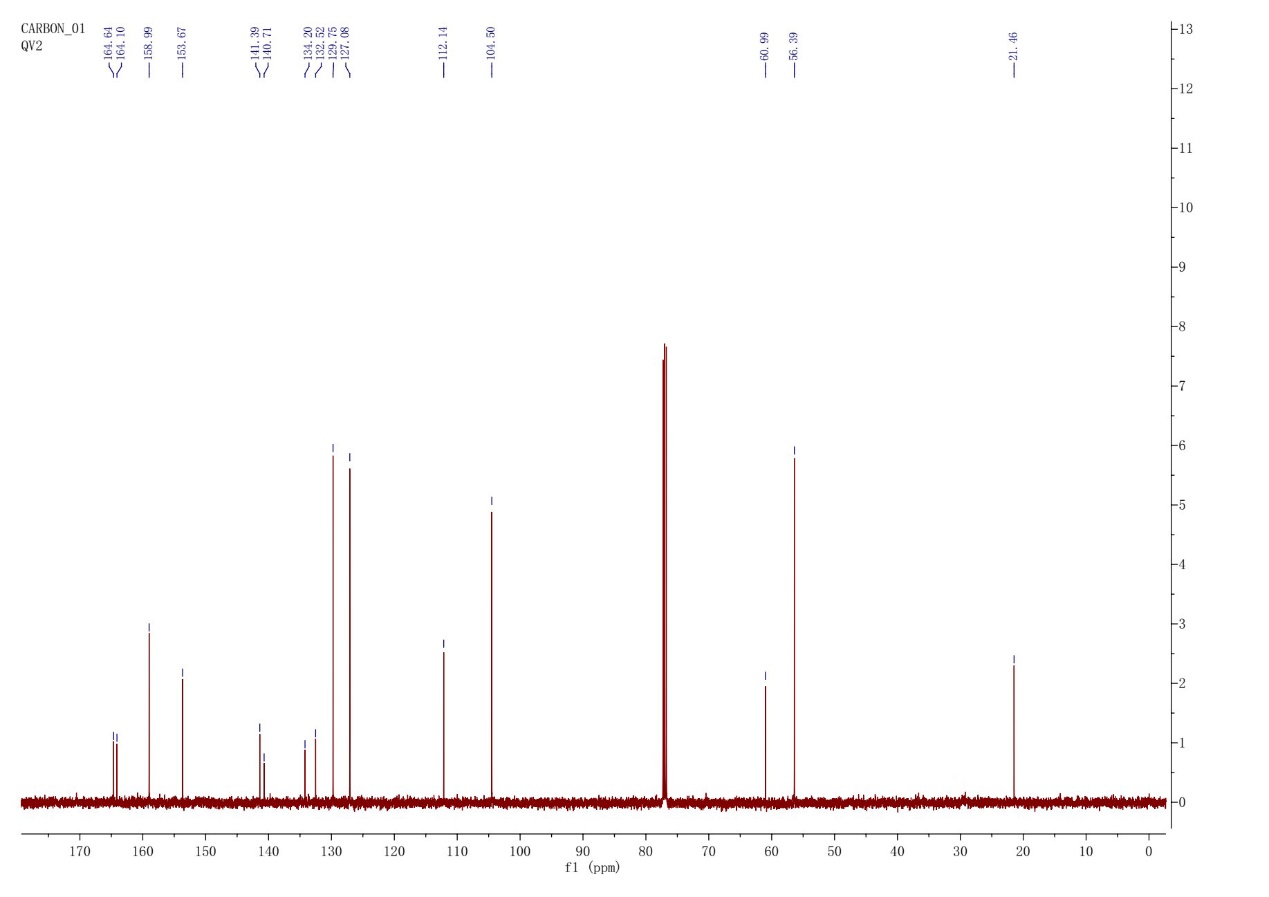
*

*4-(3,4-dimethylphenyl)-6-(3,4,5-trimethoxyphenyl)pyrimidine (****11e****)*

*
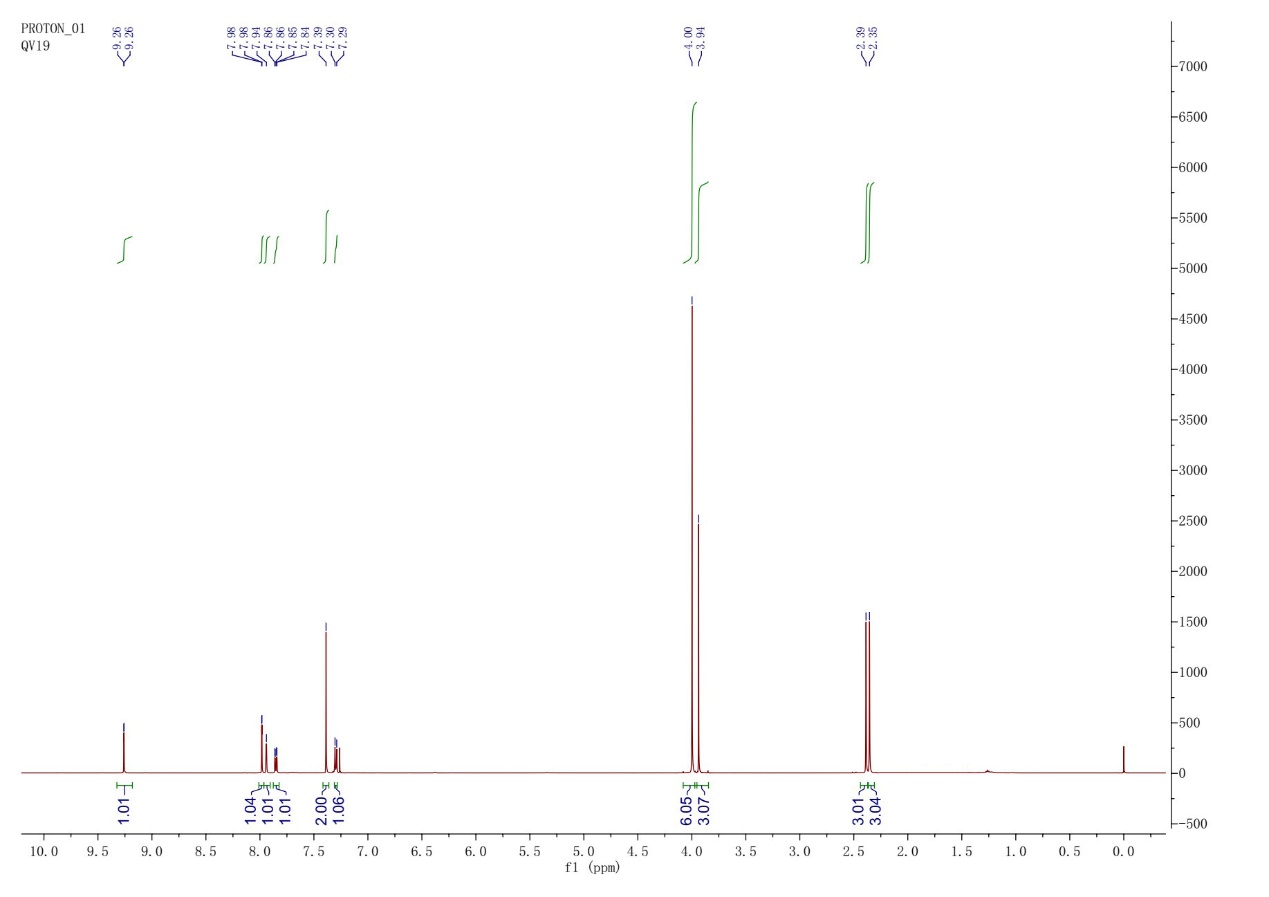
*

*
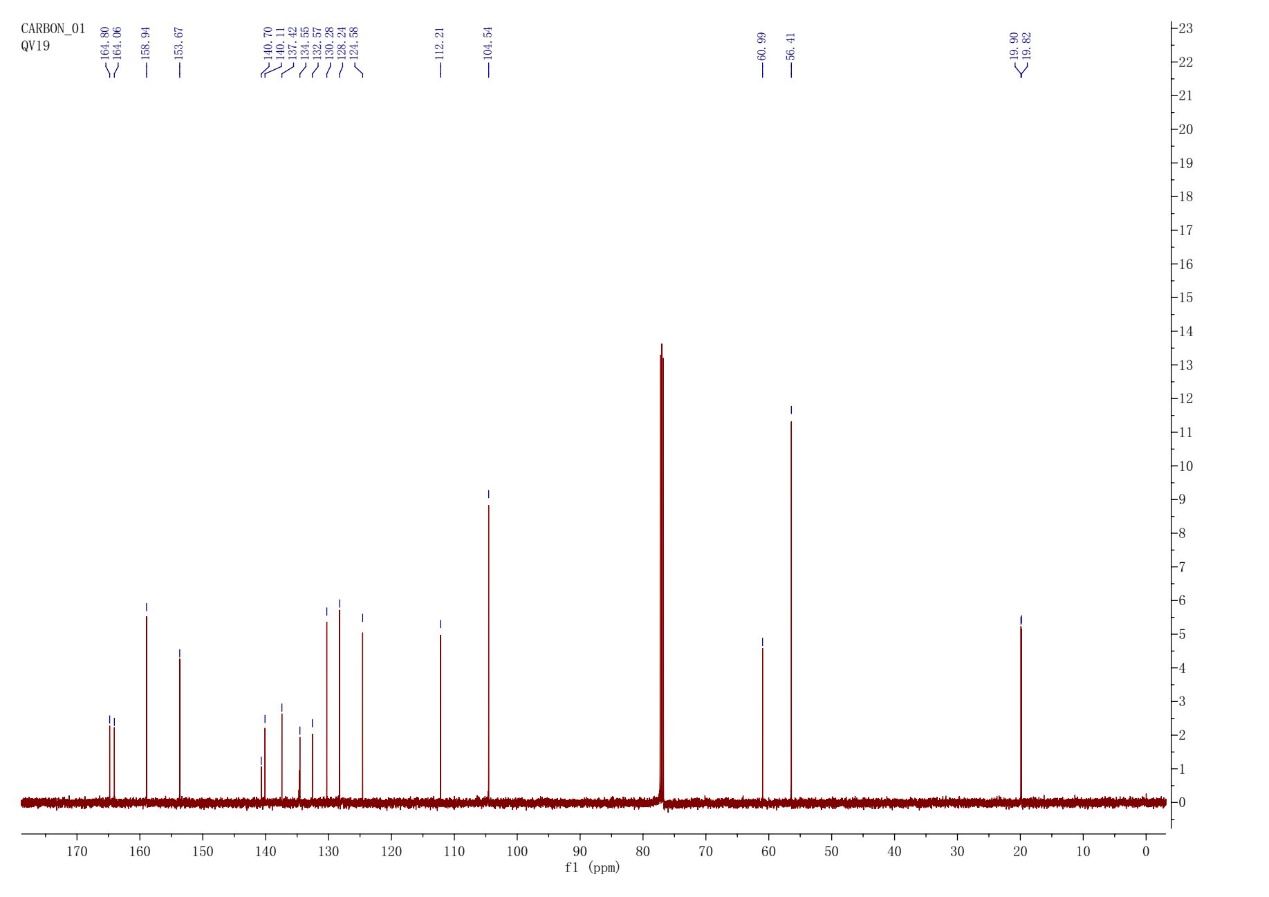
*

*4-(2-methoxyphenyl)-6-(3,4,5-trimethoxyphenyl)pyrimidine (****11f****)*

*
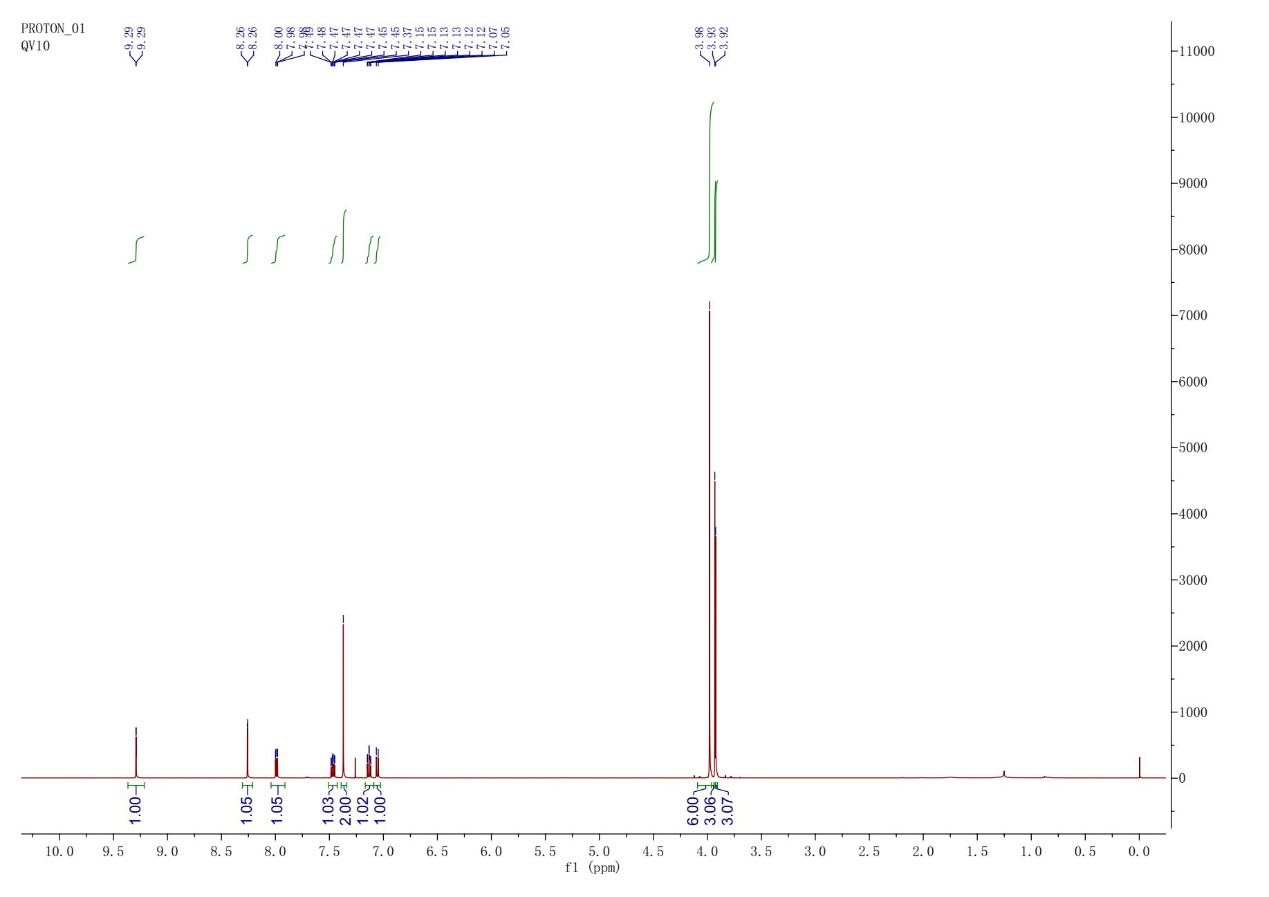
*

*
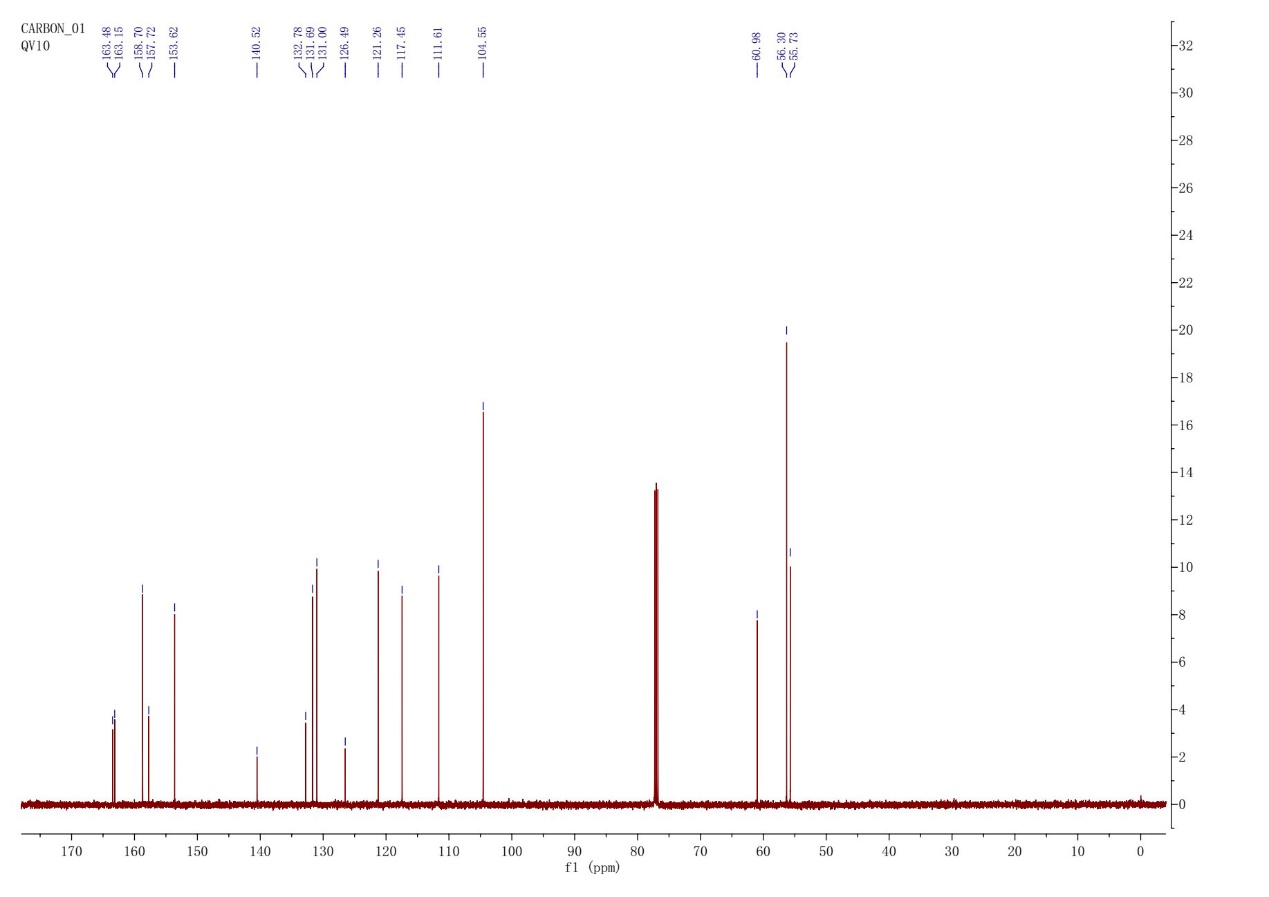
*

*4-(3-methoxyphenyl)-6-(3,4,5-trimethoxyphenyl)pyrimidine (****11g****)*

*
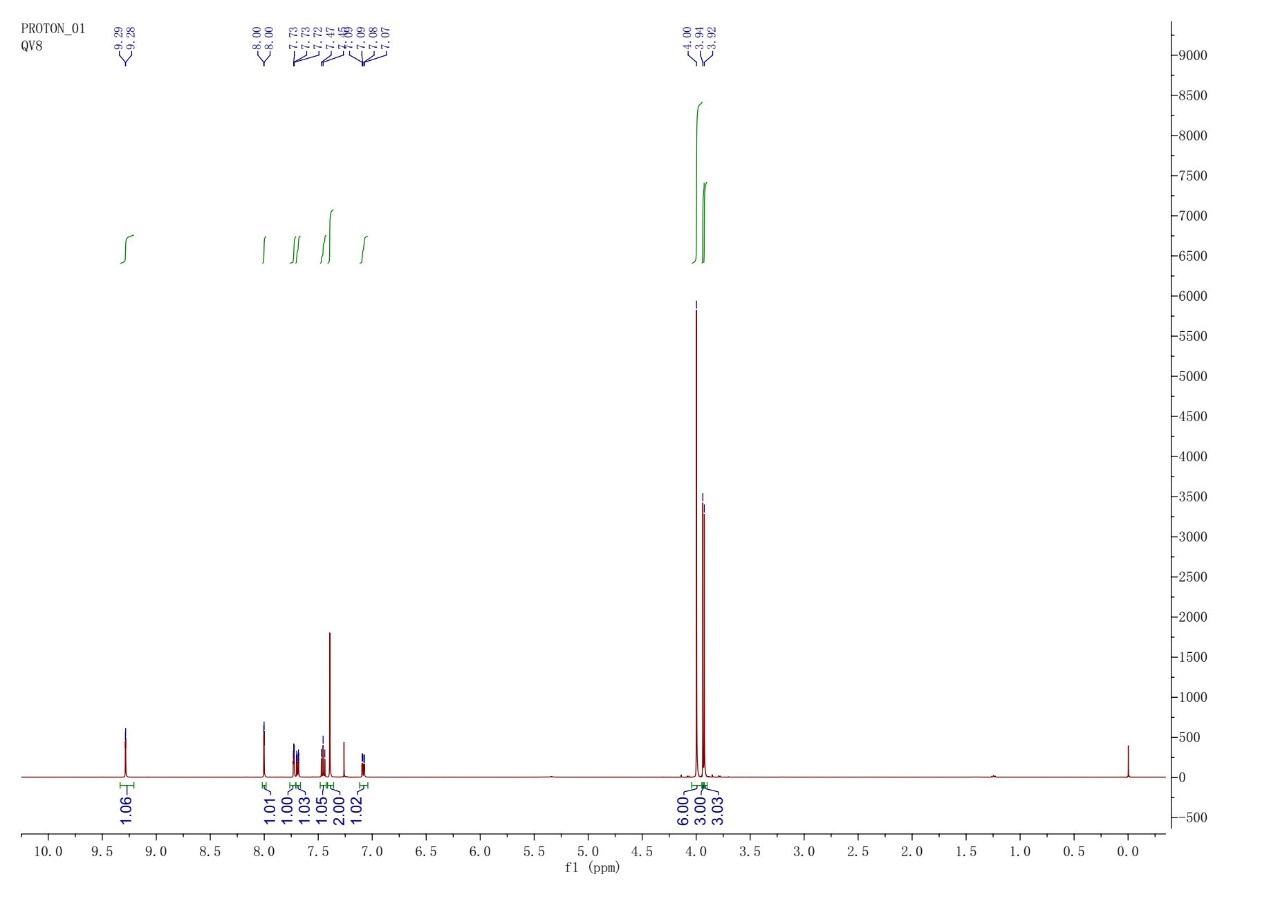
*

*
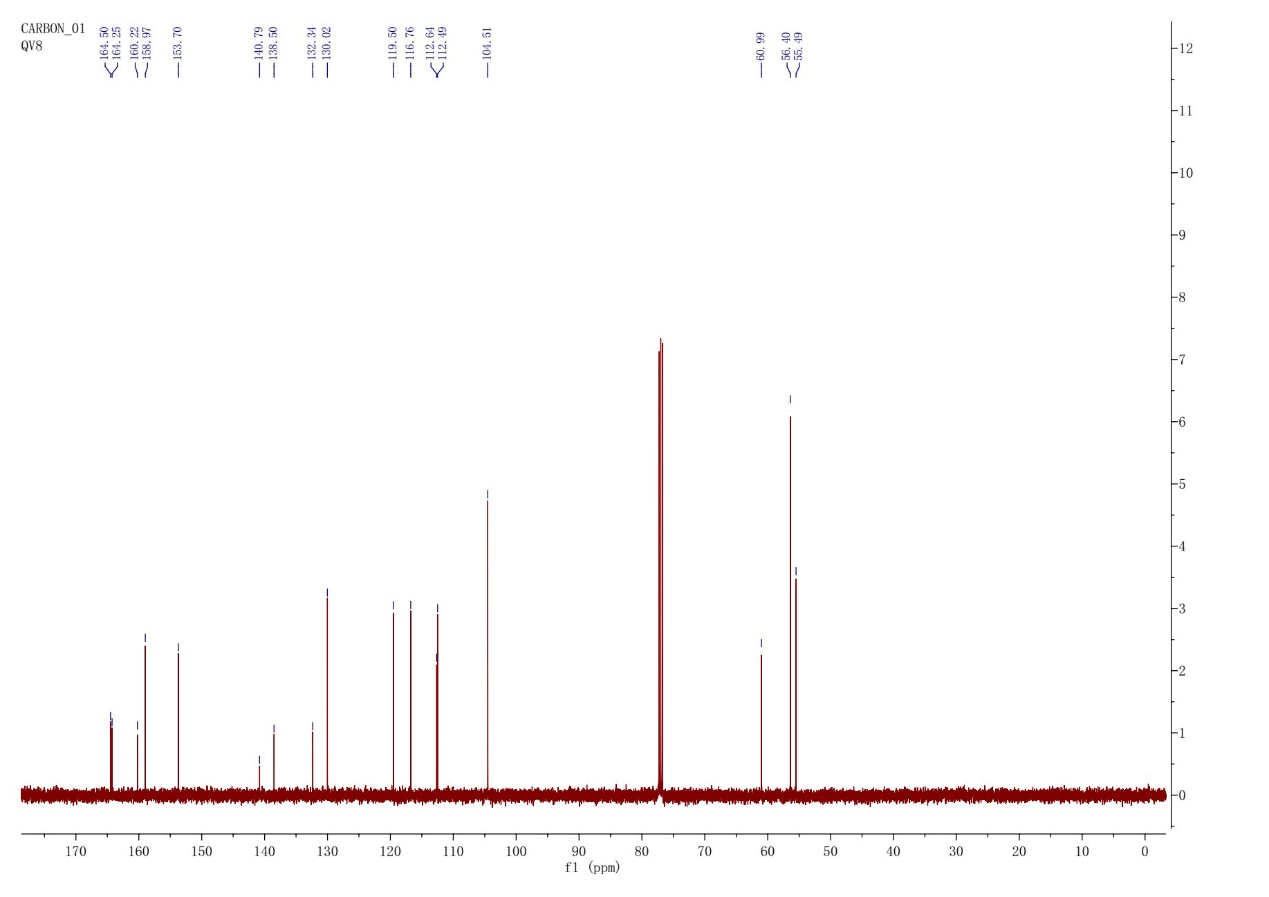
*

*4-(4-methoxyphenyl)-6-(3,4,5-trimethoxyphenyl)pyrimidine (****11h****)*

*
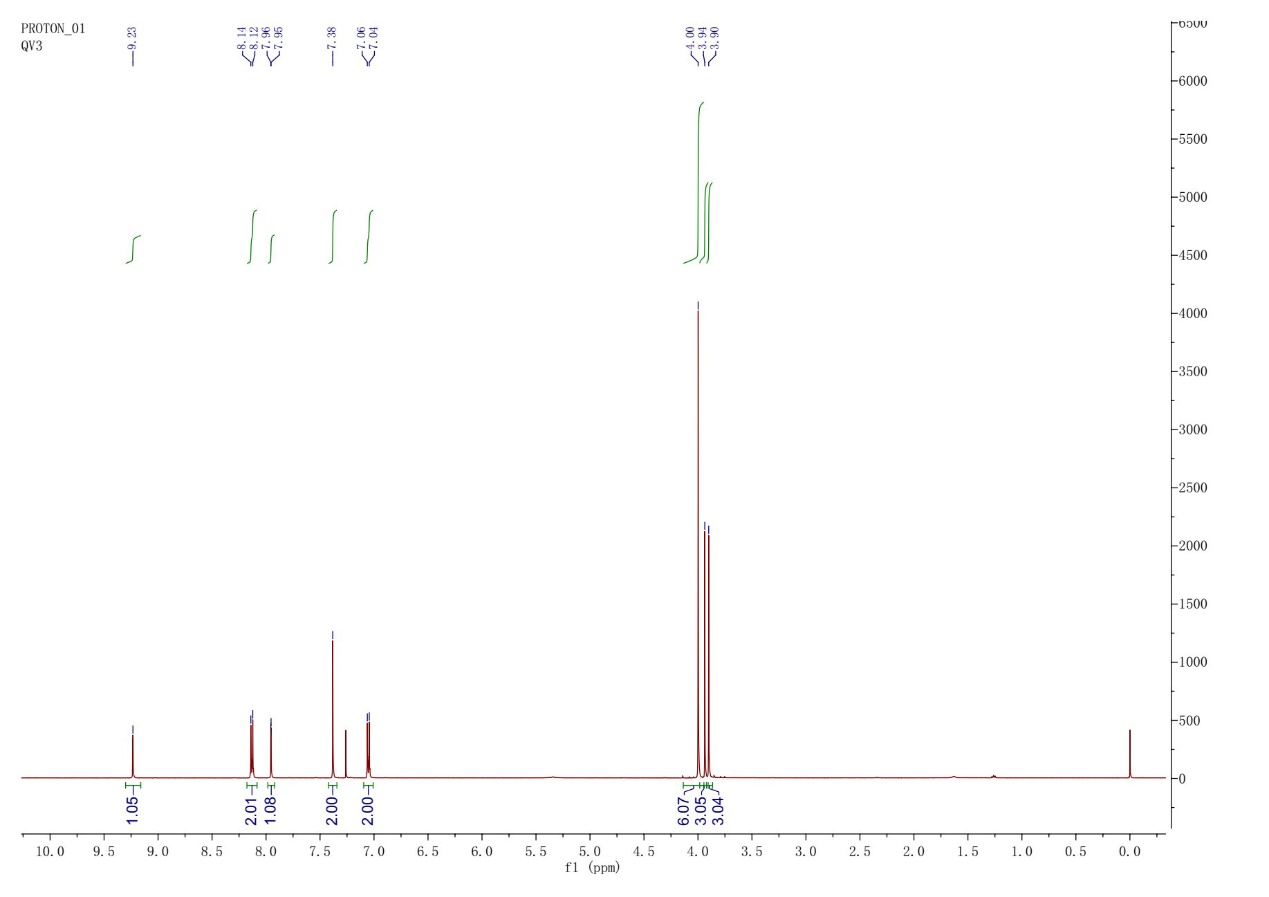
*

*
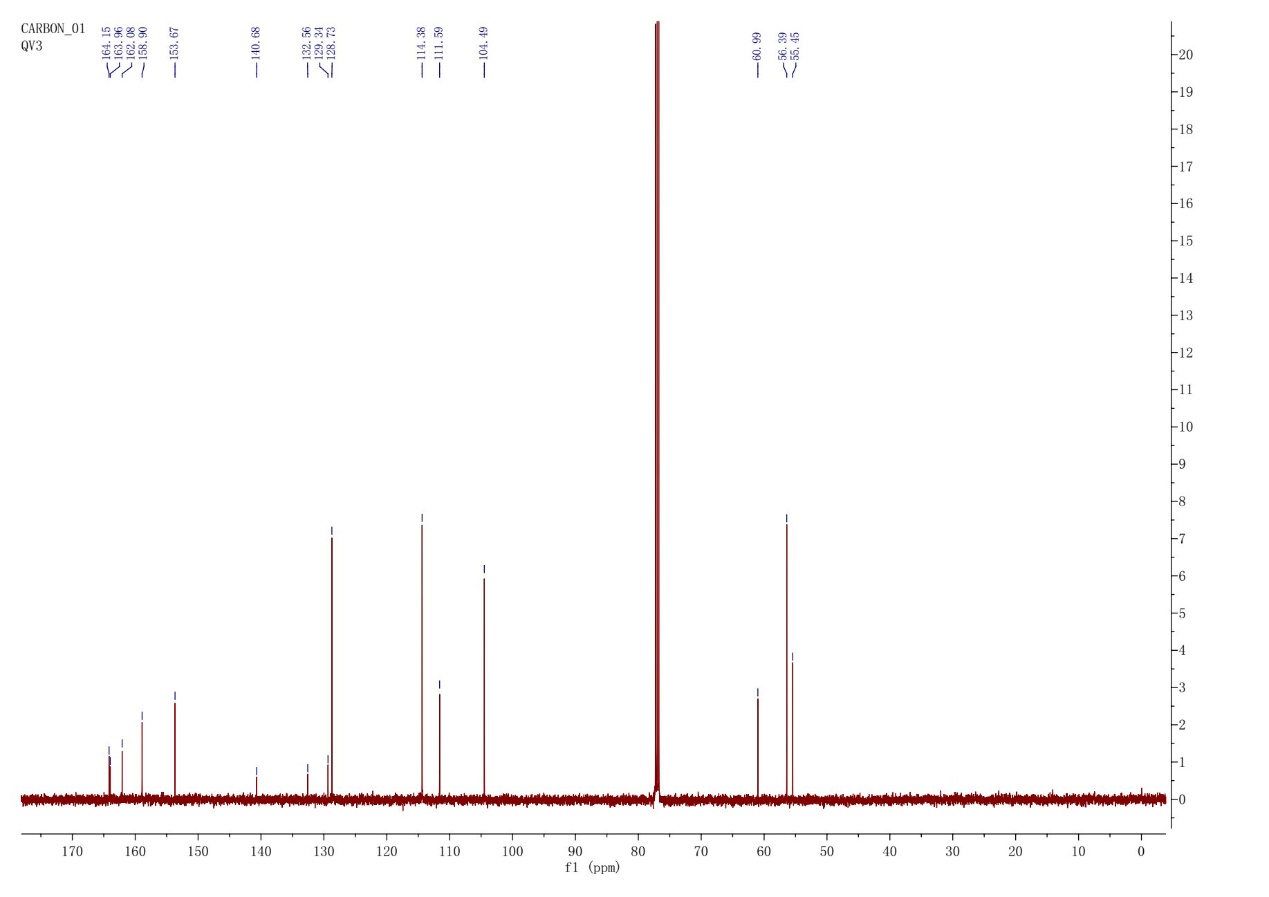
*

*4-(4-ethoxyphenyl)-6-(3,4,5-trimethoxyphenyl)pyrimidine (****11i****)*

*
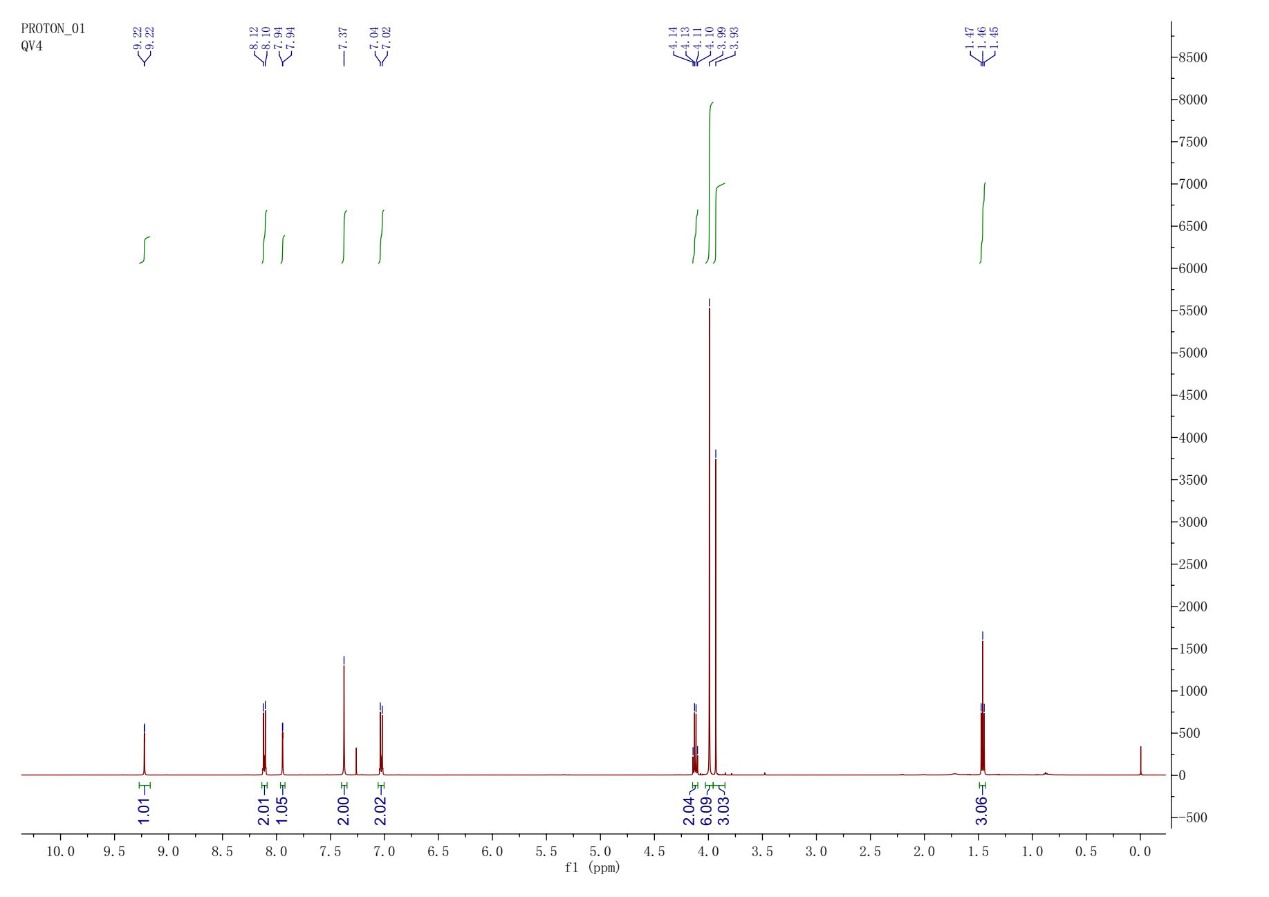
*

*
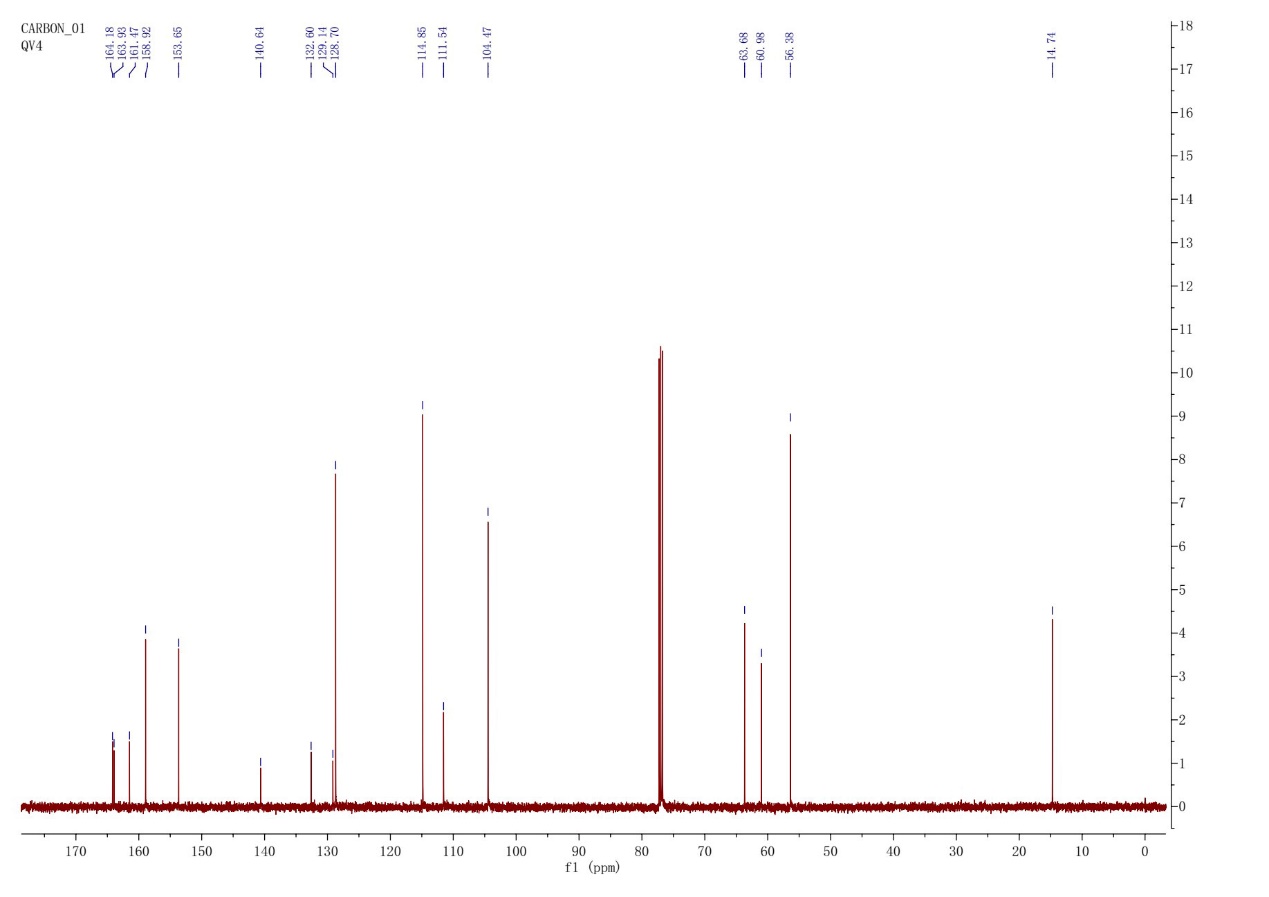
*

*2-methoxy-5-(6-(3,4,5-trimethoxyphenyl)pyrimidin-4-yl)phenol (****11j****)*

*
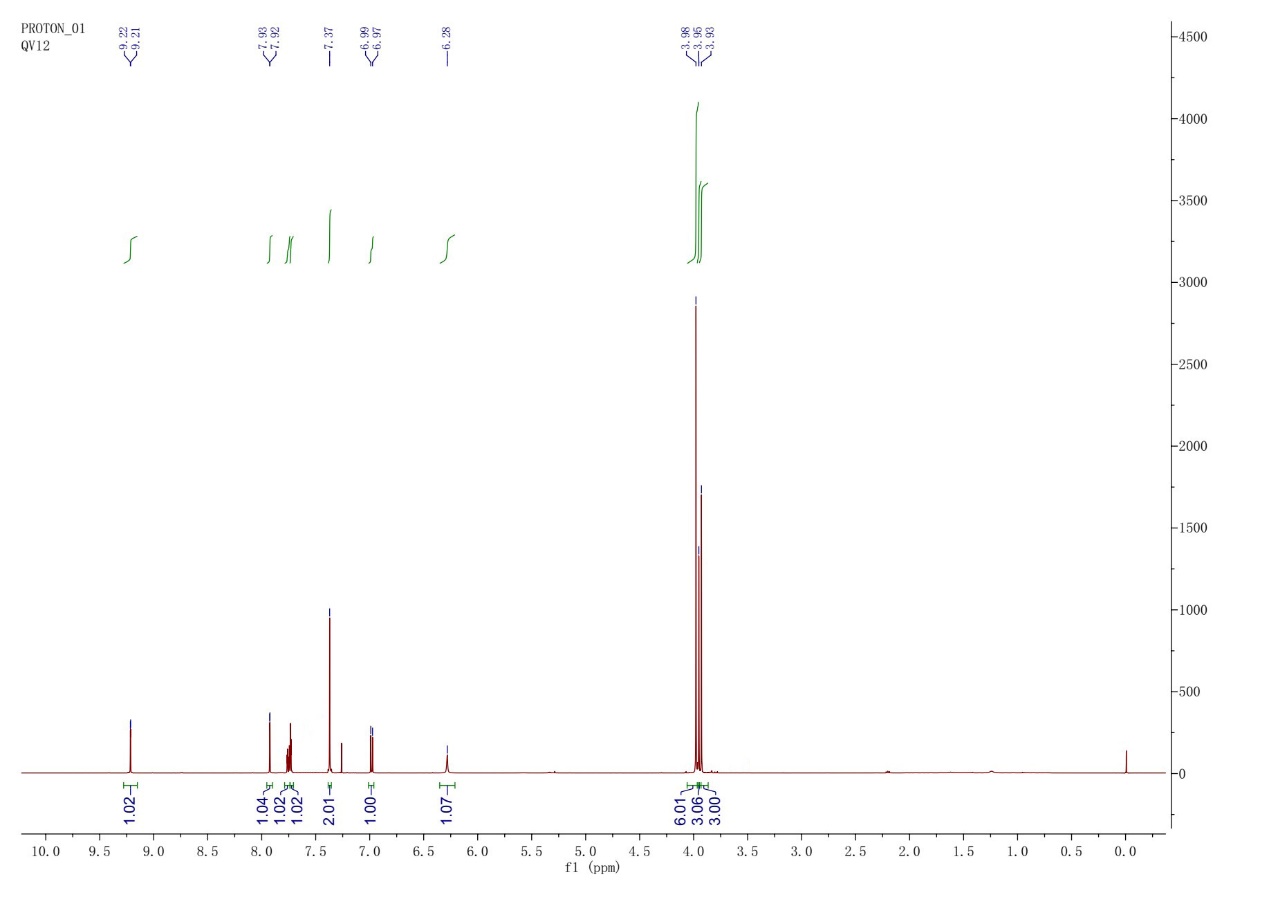
*

*
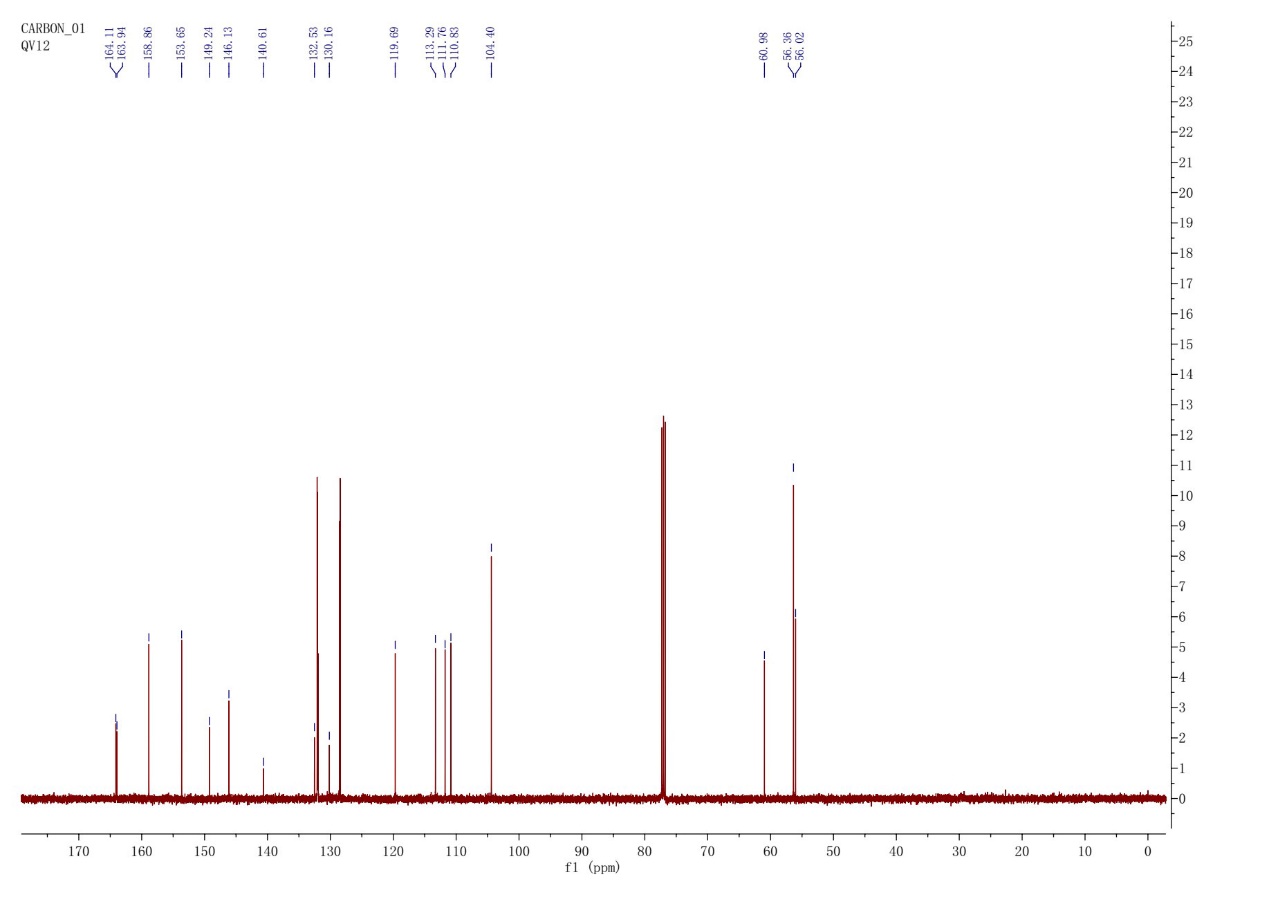
*

*4-(3,4-dimethoxyphenyl)-6-(3,4,5-trimethoxyphenyl)pyrimidine (****11k****)*

*
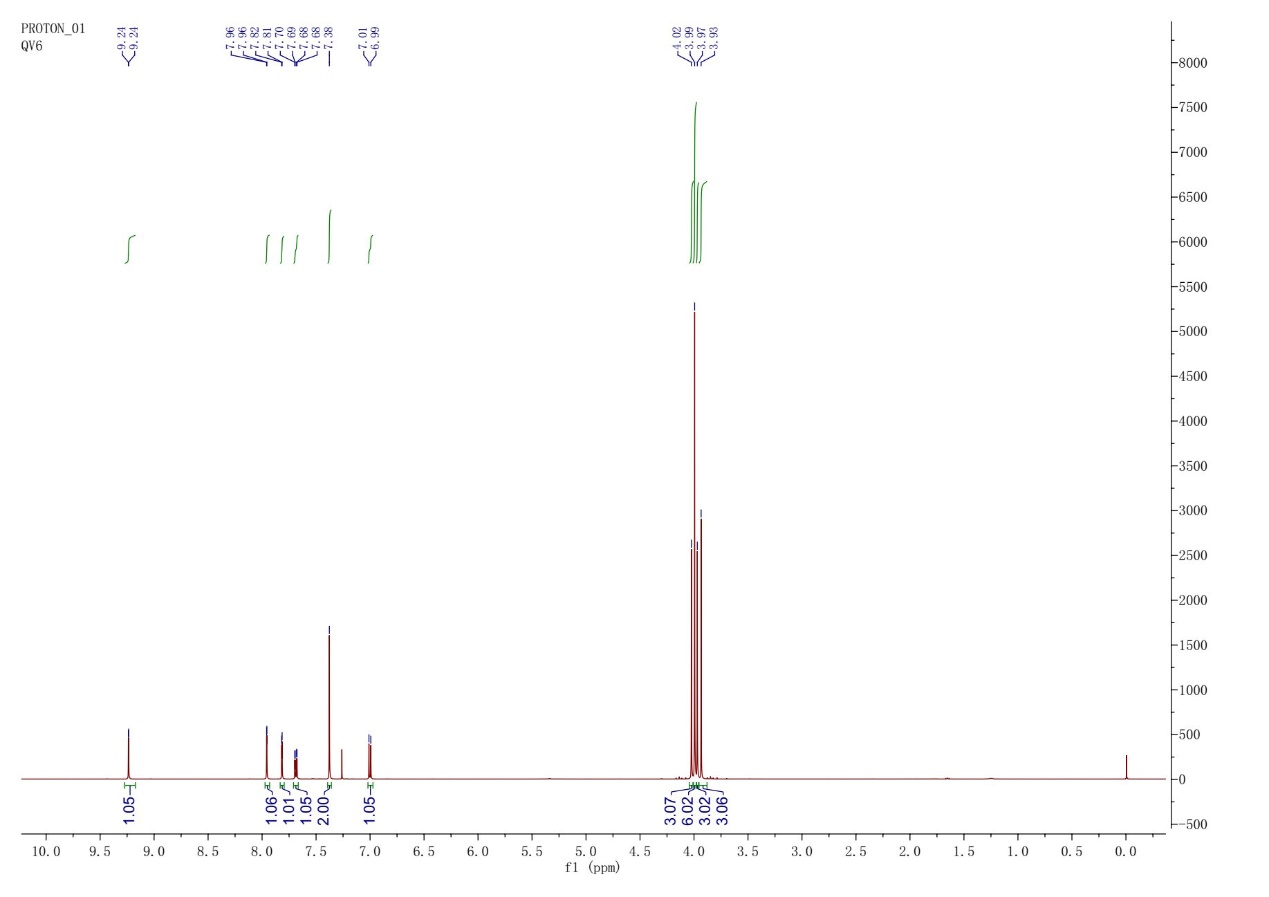
*

*
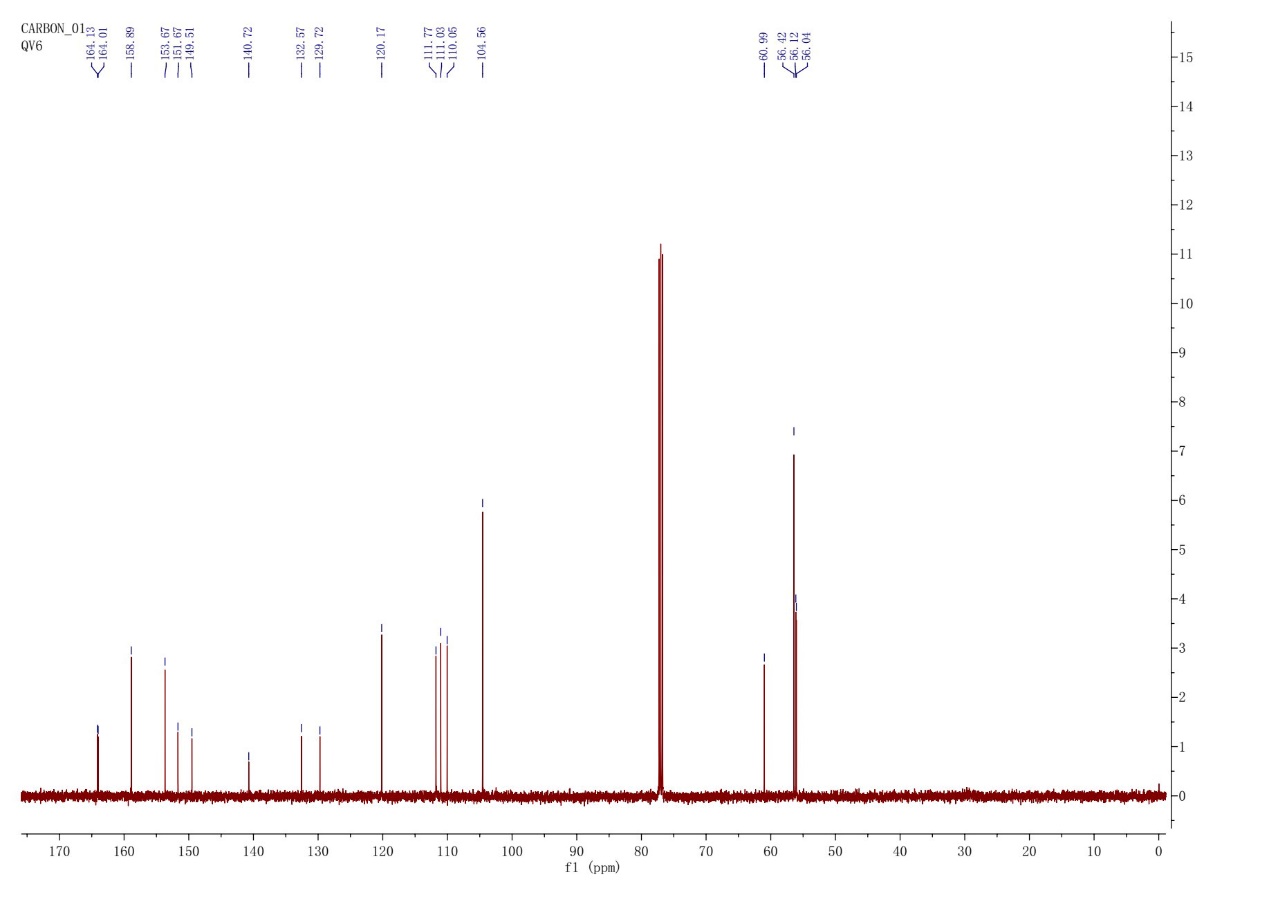
*

*4-(4-nitrophenyl)-6-(3,4,5-trimethoxyphenyl)pyrimidine (****11l****)*

*
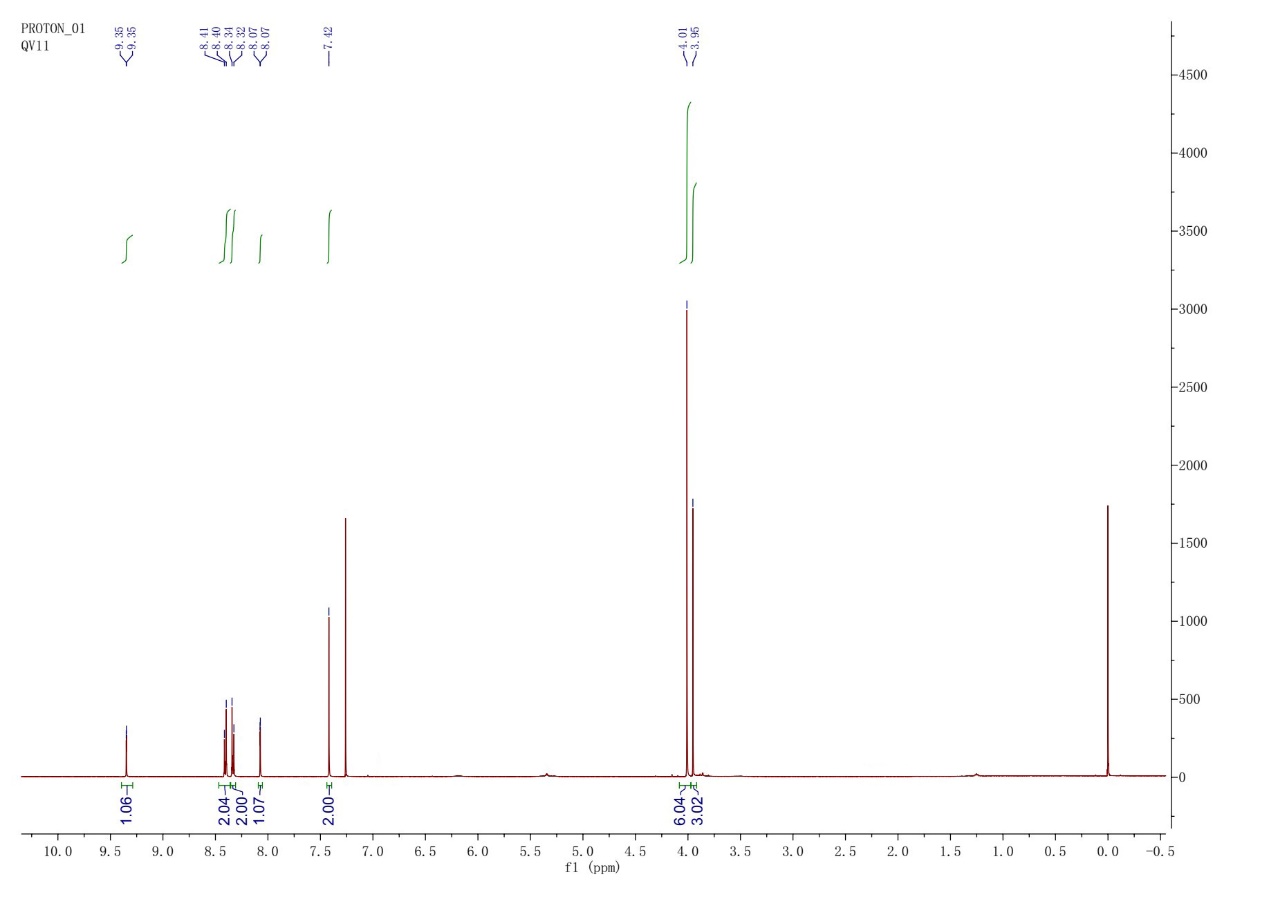
*

*
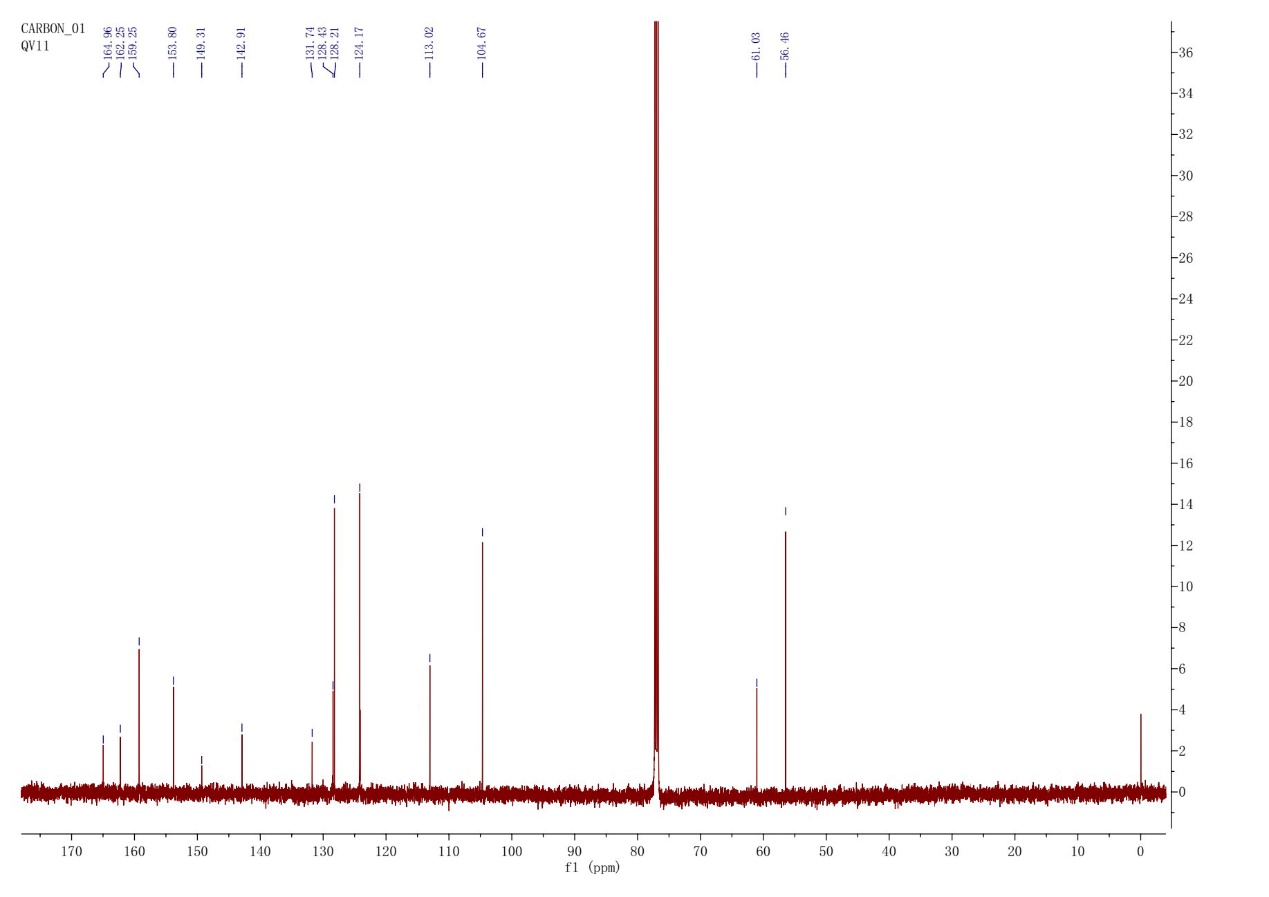
*

*4-(6-(3,4,5-trimethoxyphenyl)pyrimidin-4-yl)phenol (****11m****)*

*
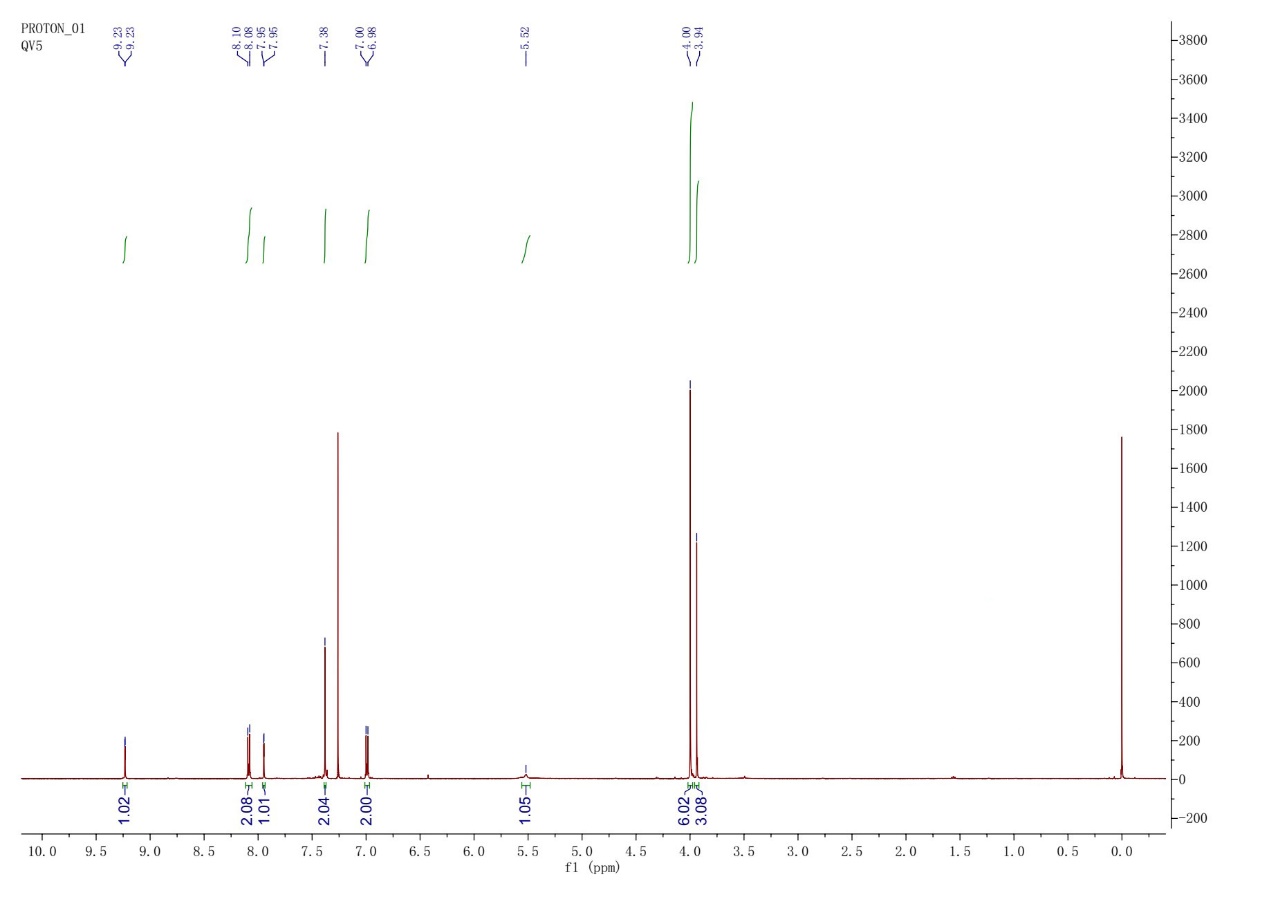
*

*
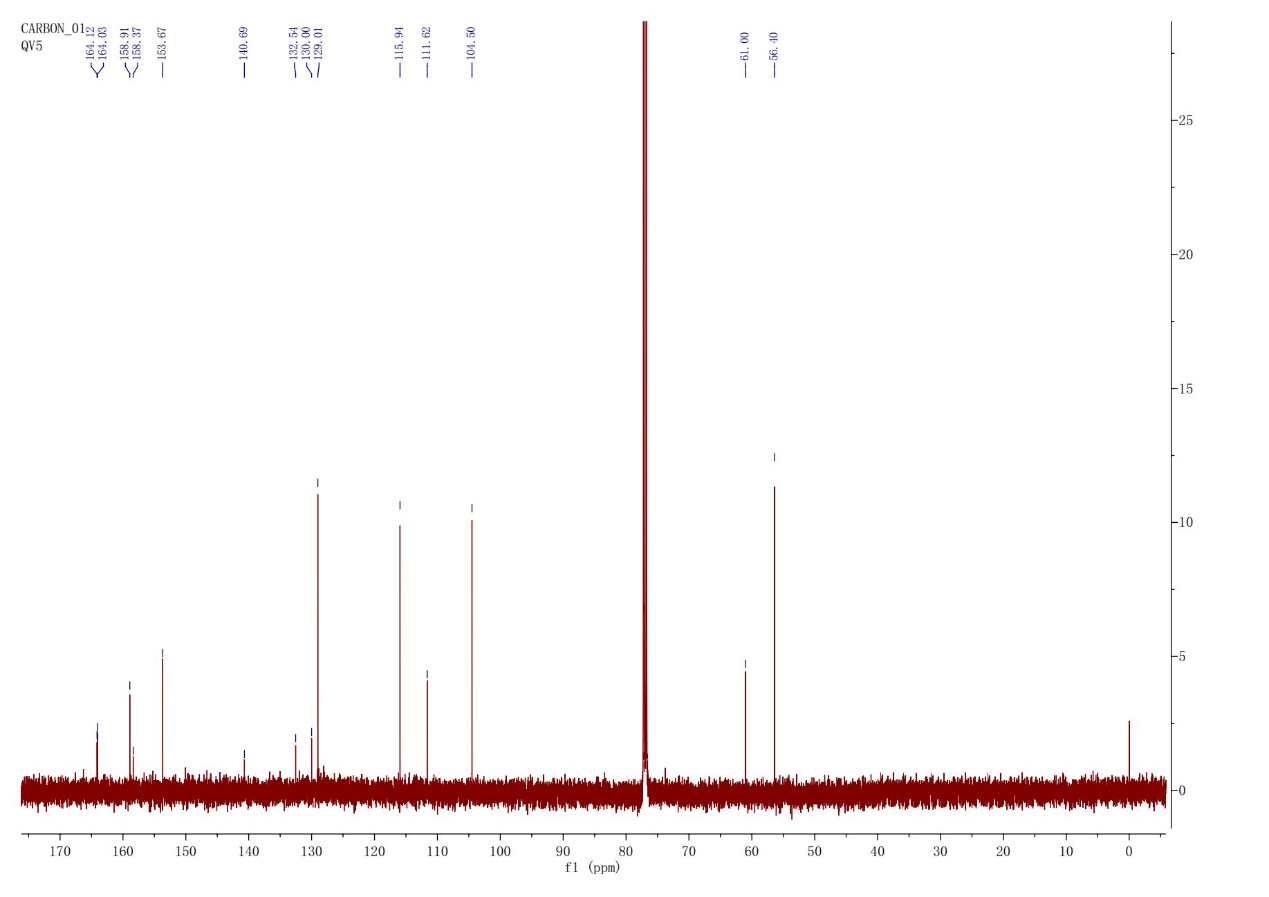
*

*4-(4-fluorophenyl)-6-(3,4,5-trimethoxyphenyl)pyrimidine (****11n****)*

*
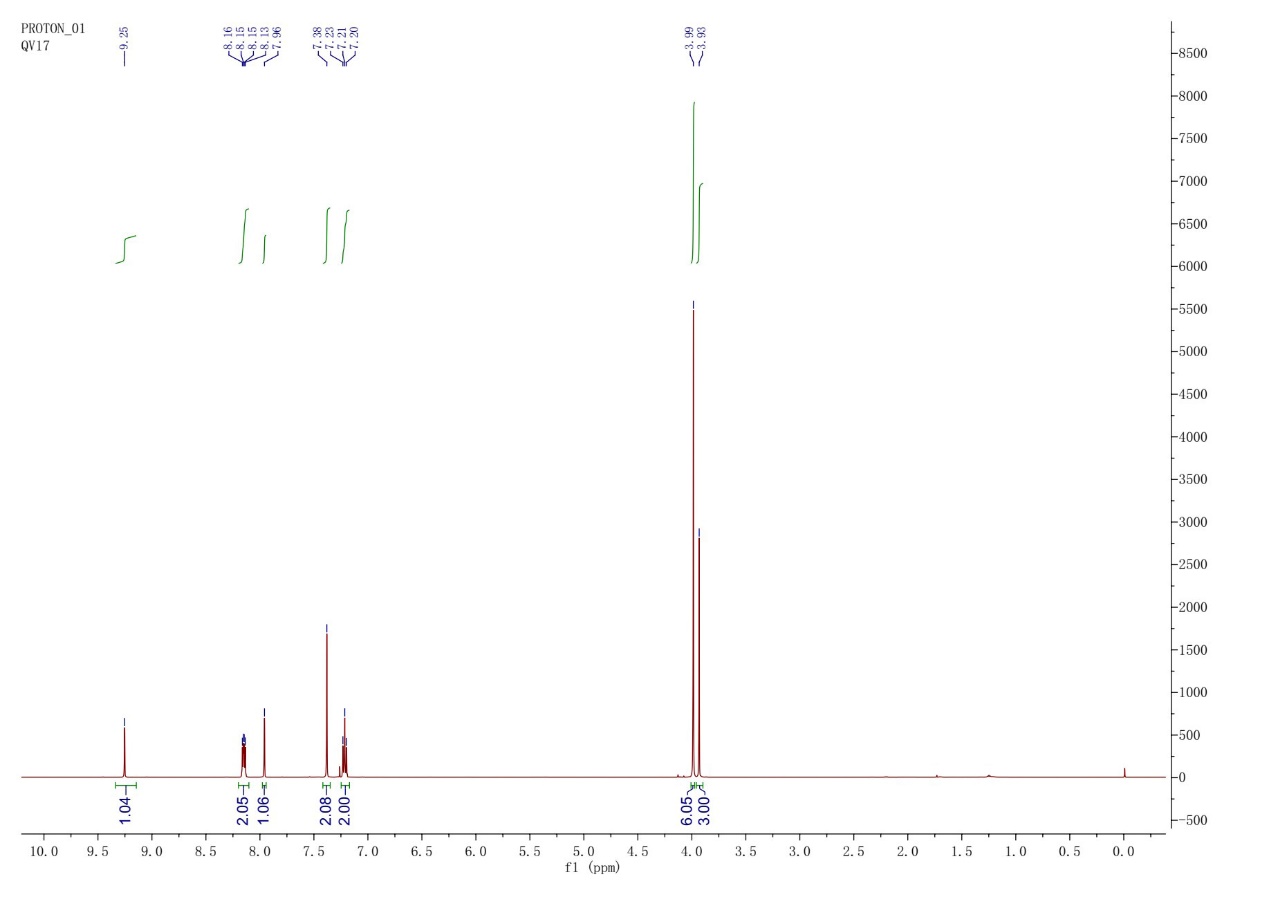
*

*
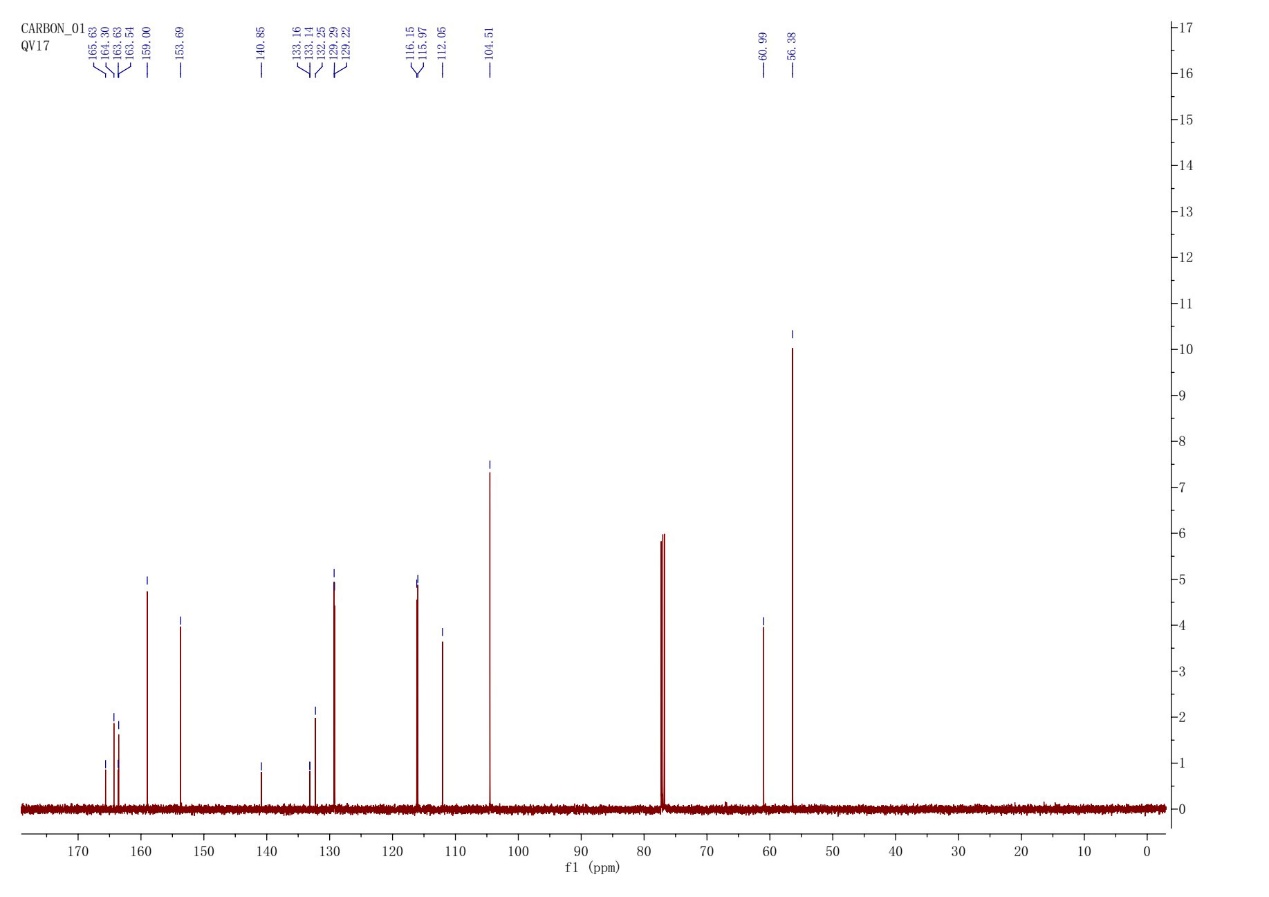
*

*4-(4-chlorophenyl)-6-(3,4,5-trimethoxyphenyl)pyrimidine* *(****11o****)*

*
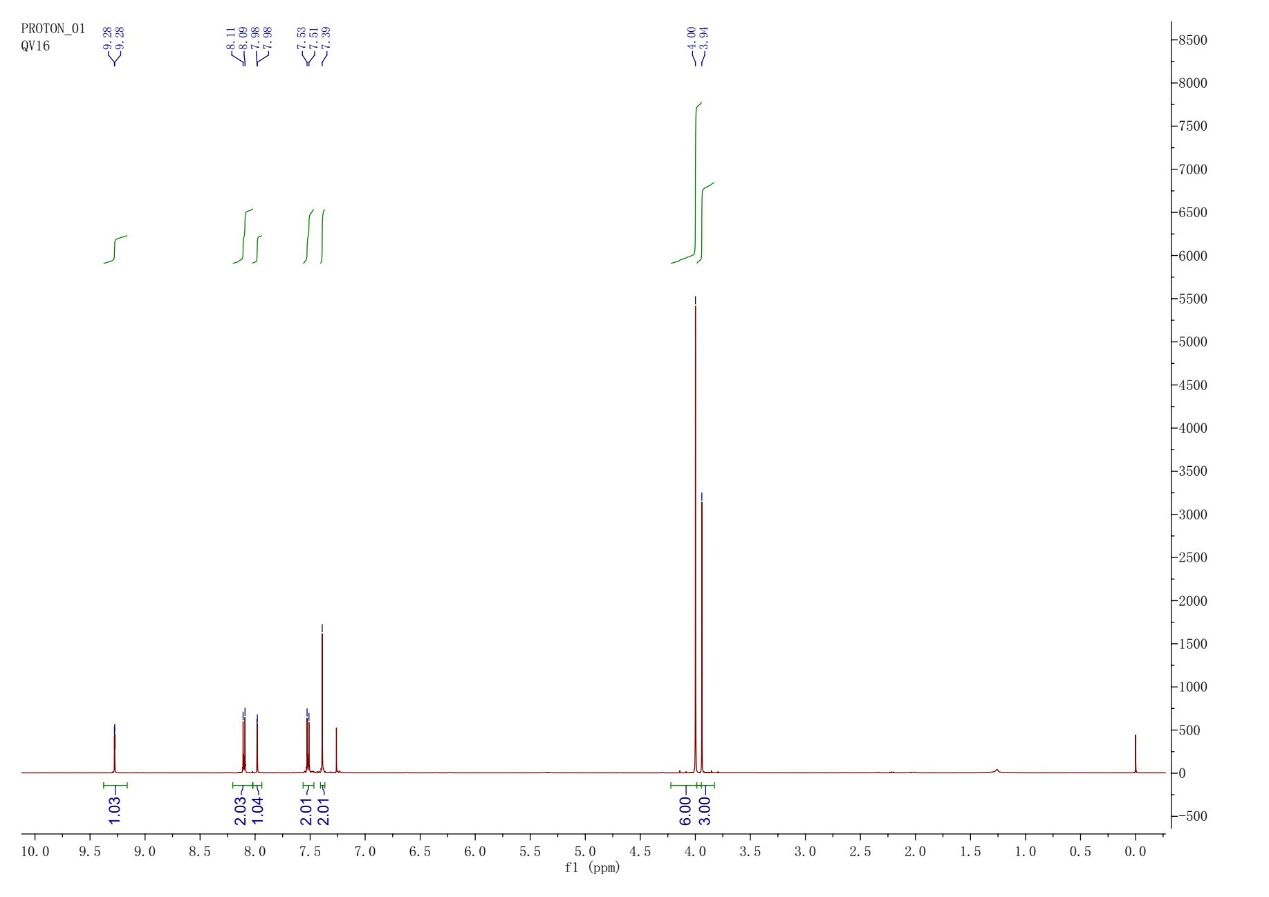
*

*
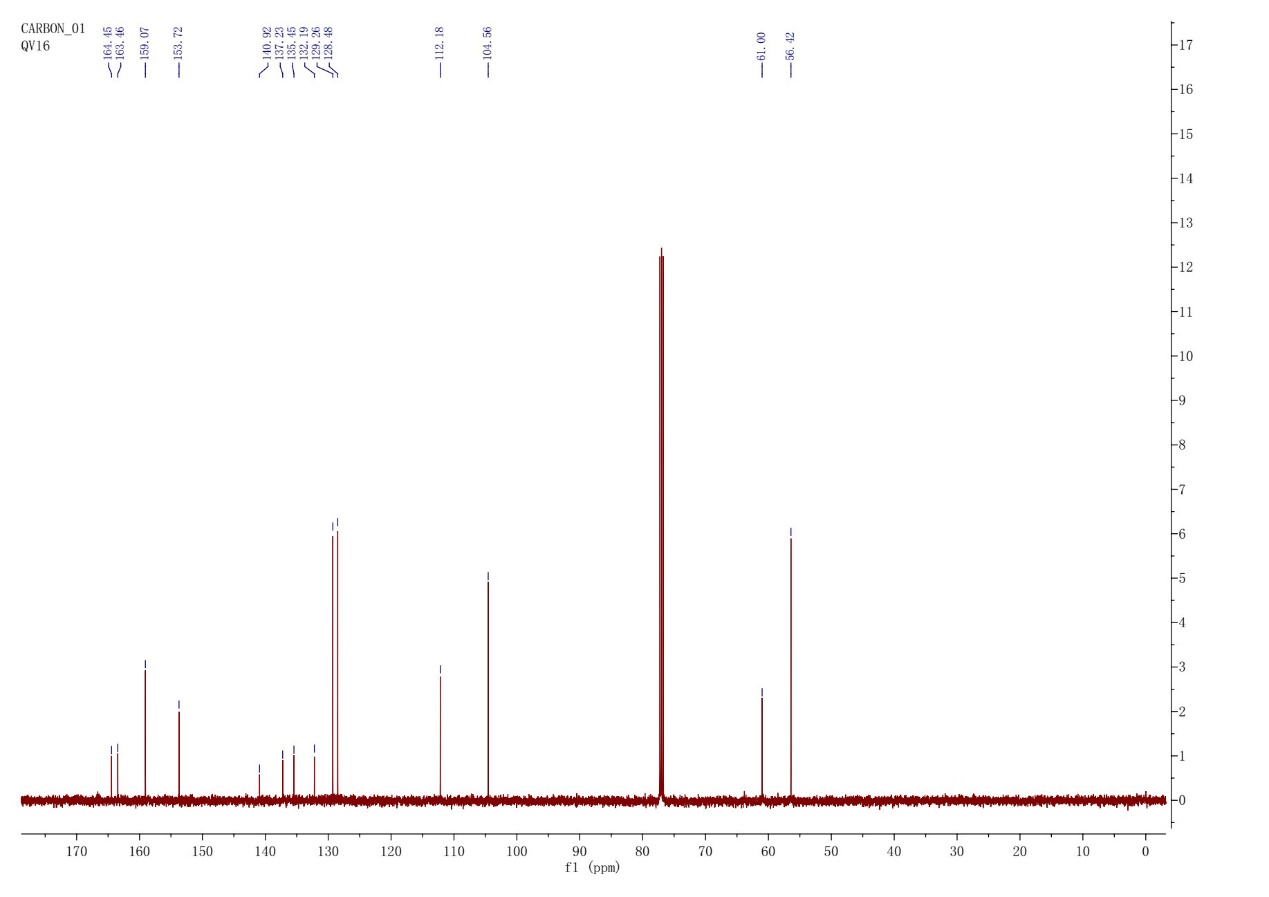
*

*4-(thiophen-3-yl)-6-(3,4,5-trimethoxyphenyl)pyrimidine (****11p****)*

*
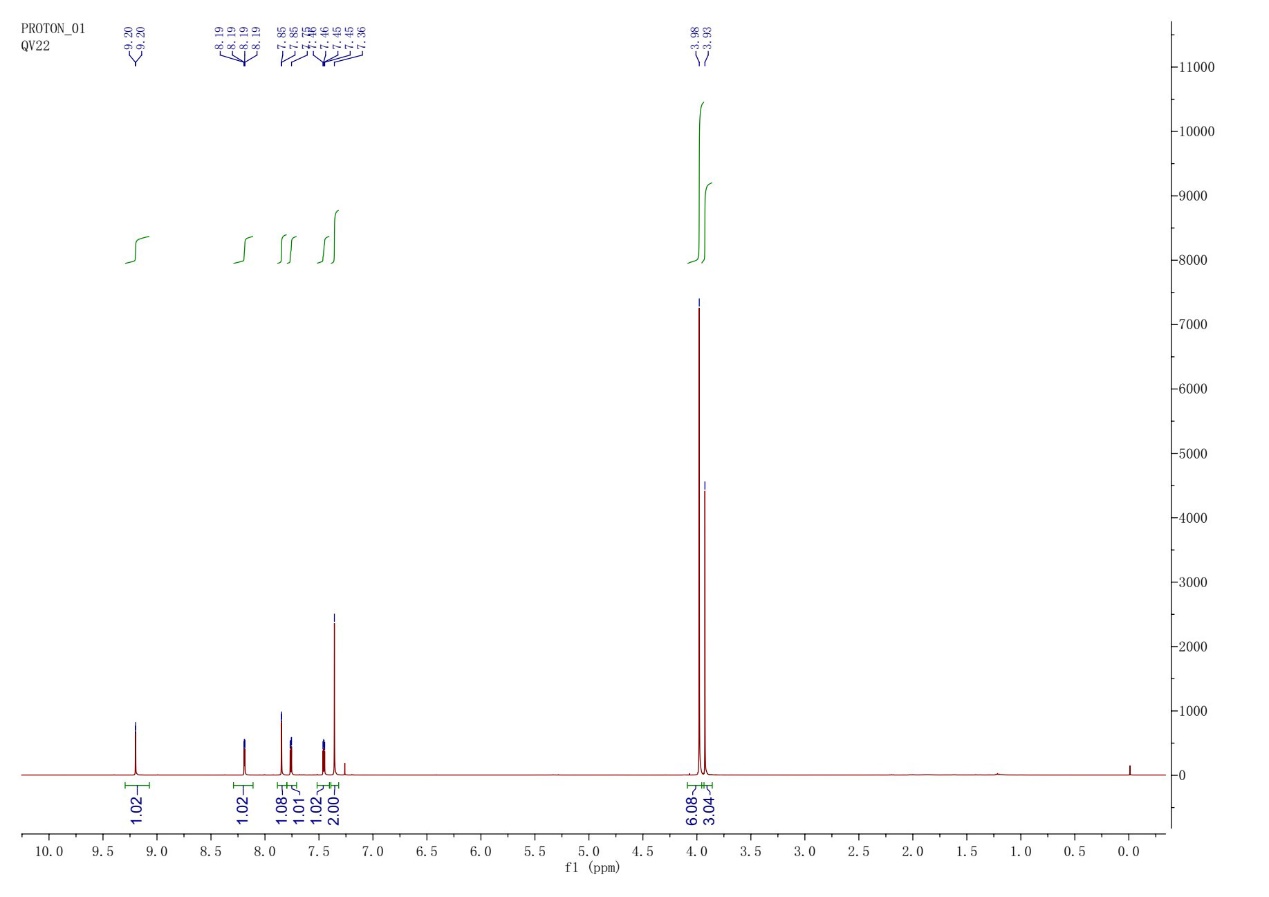
*

*
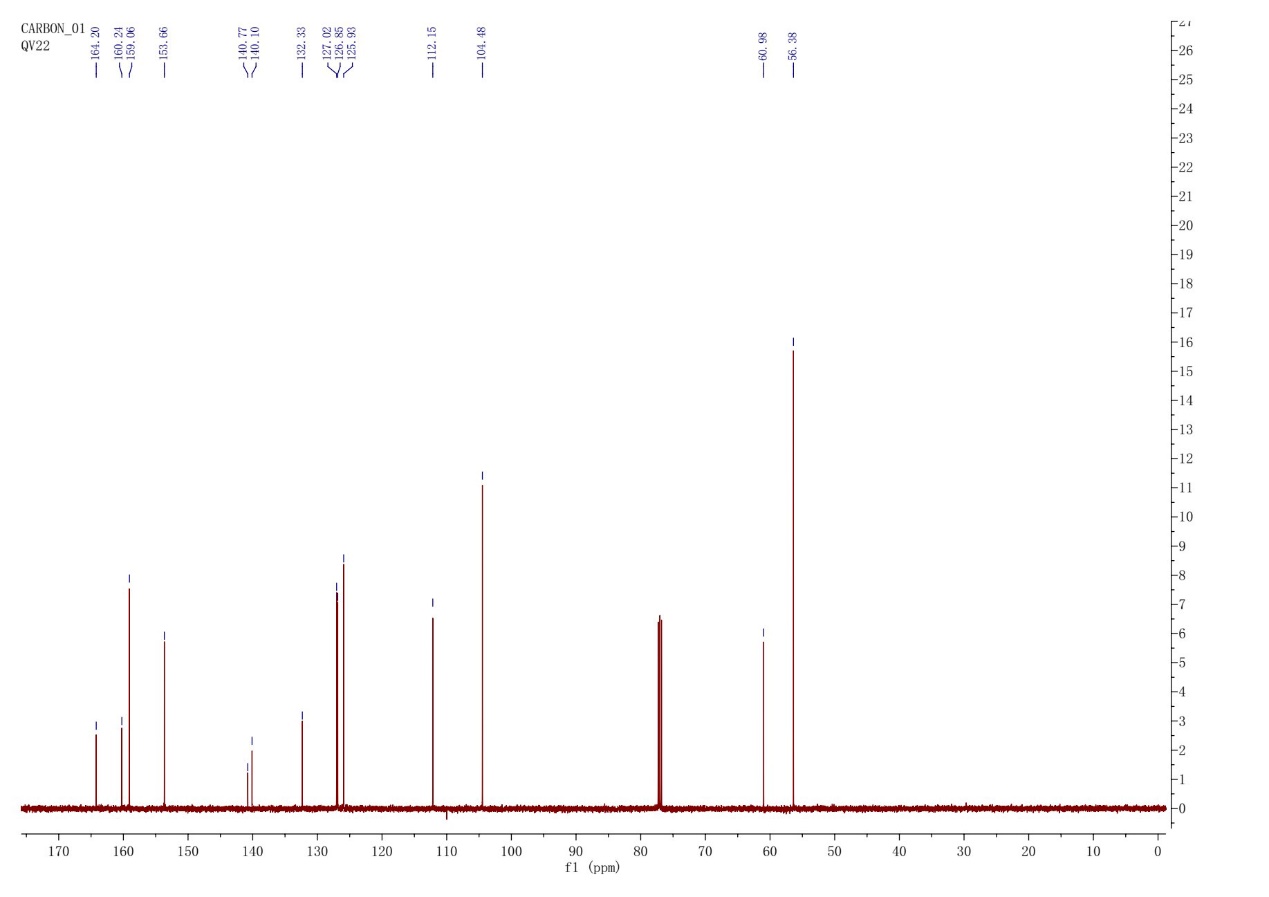
*

*4-(pyridin-3-yl)-6-(3,4,5-trimethoxyphenyl)pyrimidine (****11q****)*

*
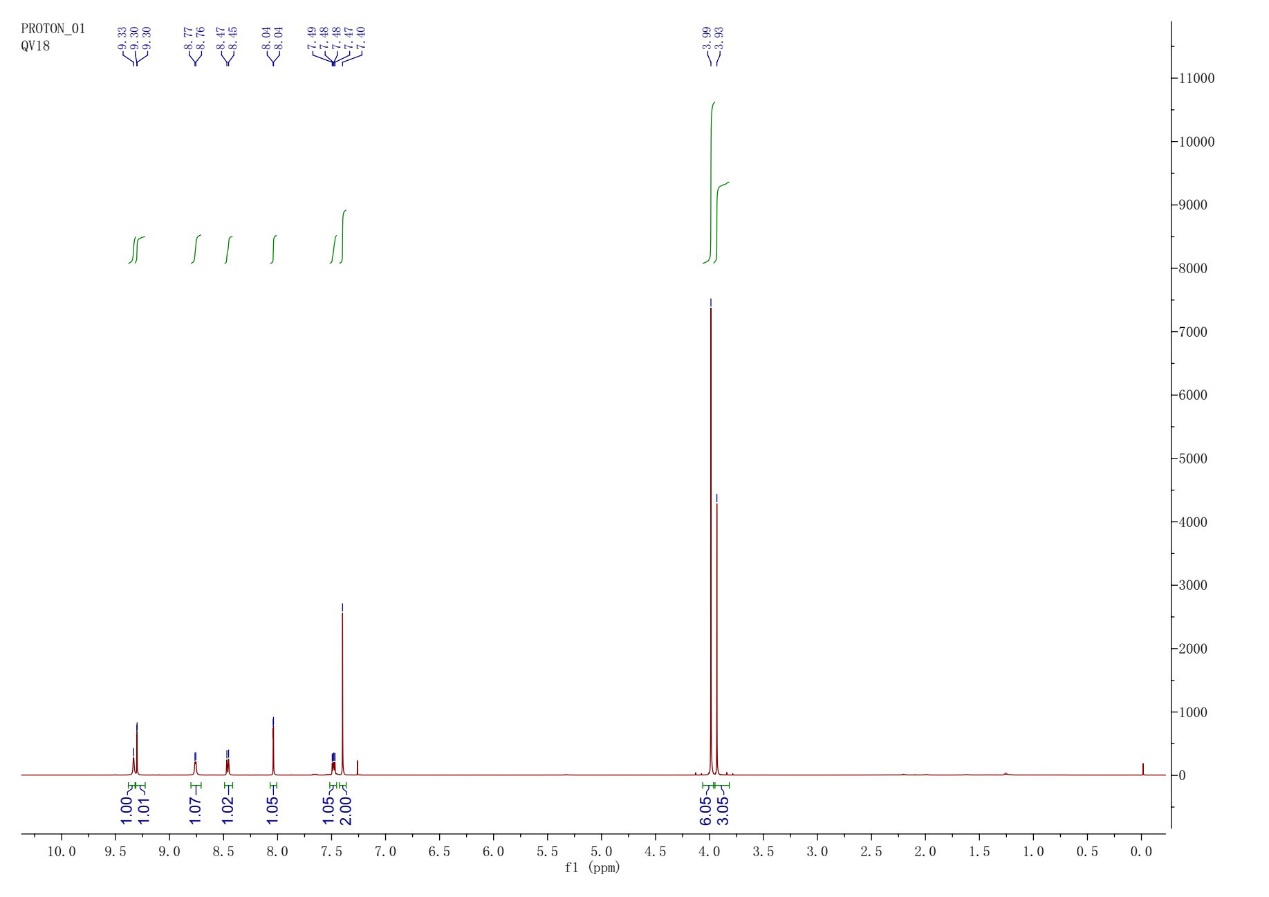
*

*
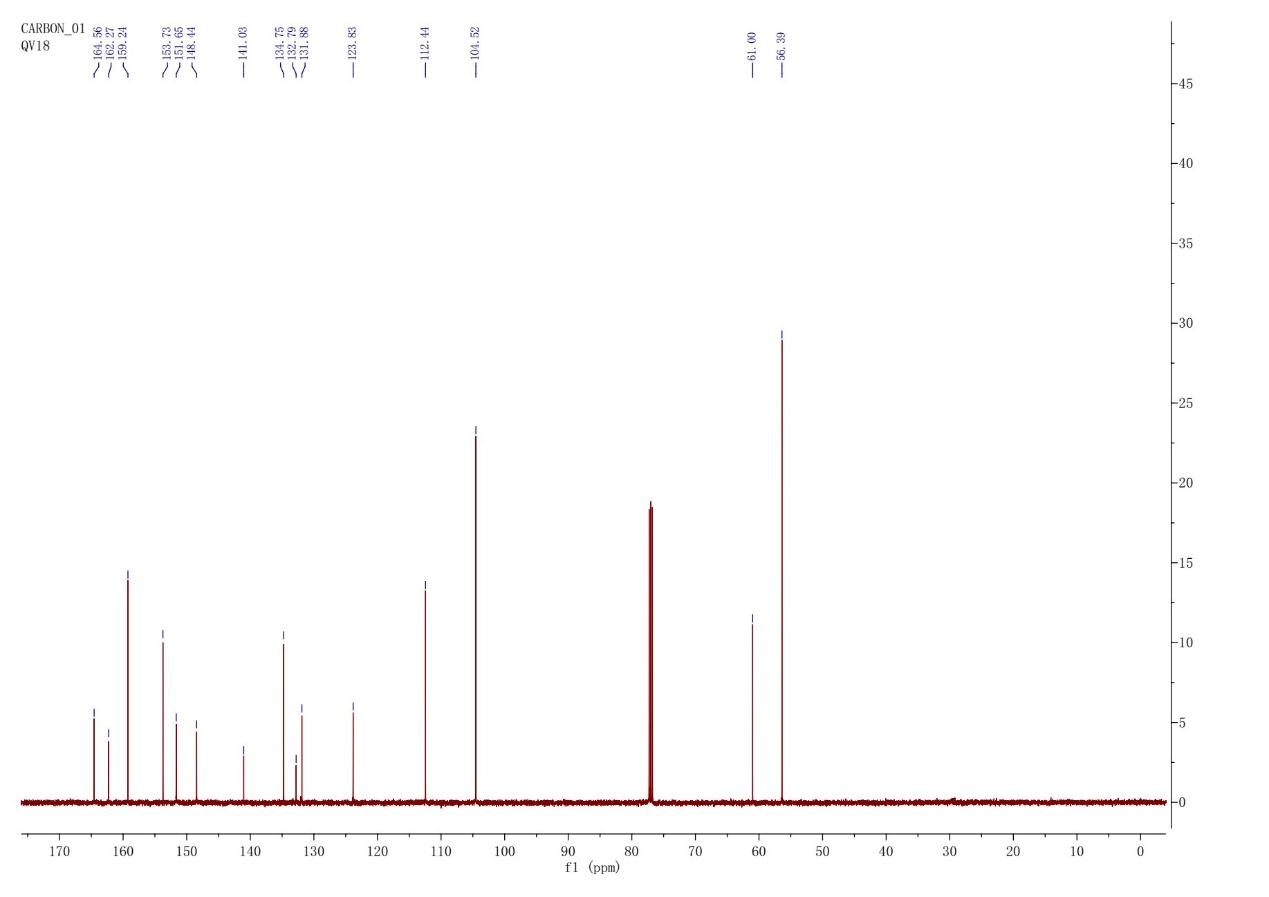
*

*4-(pyridin-4-yl)-6-(3,4,5-trimethoxyphenyl)pyrimidine (****11r****)*

*
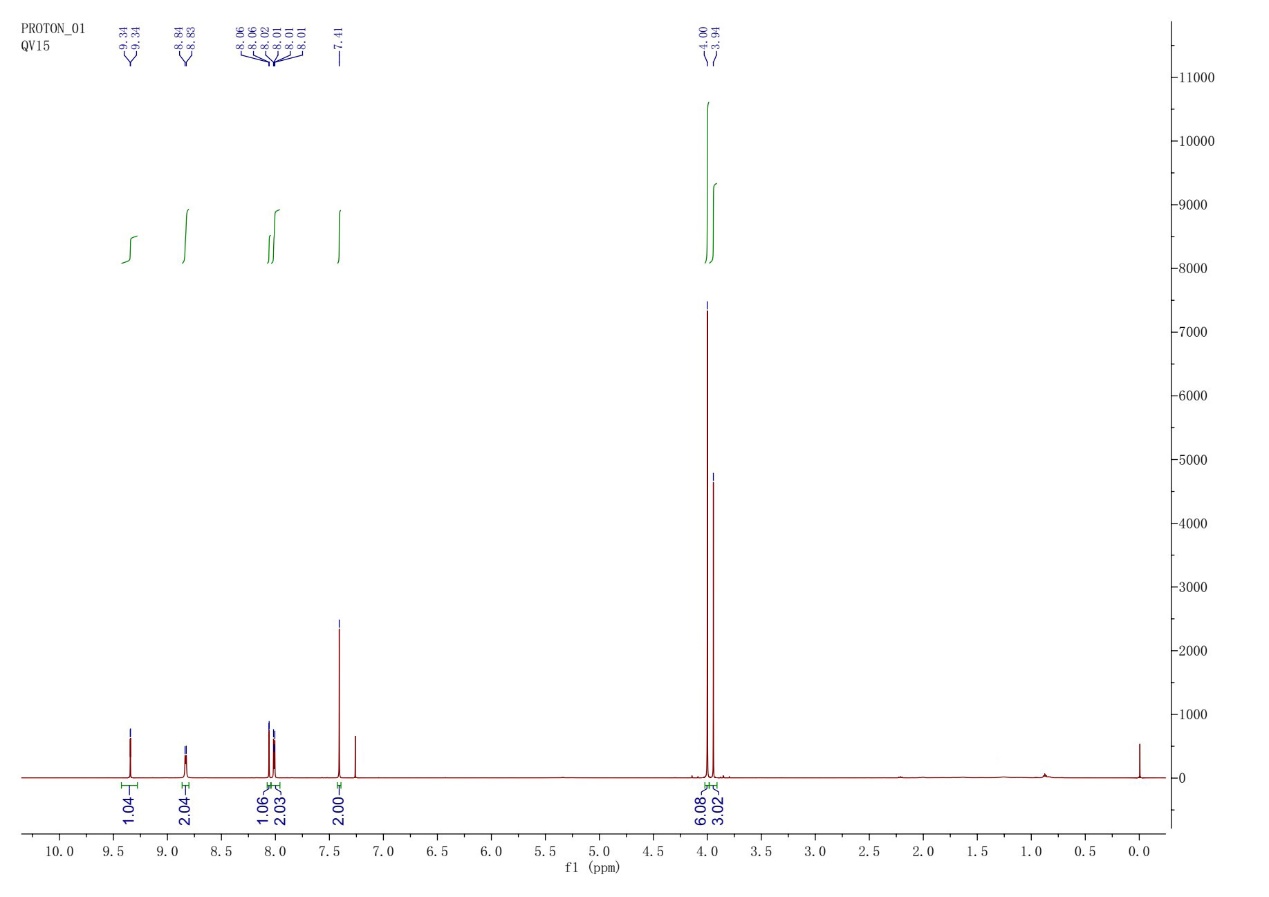
*

*
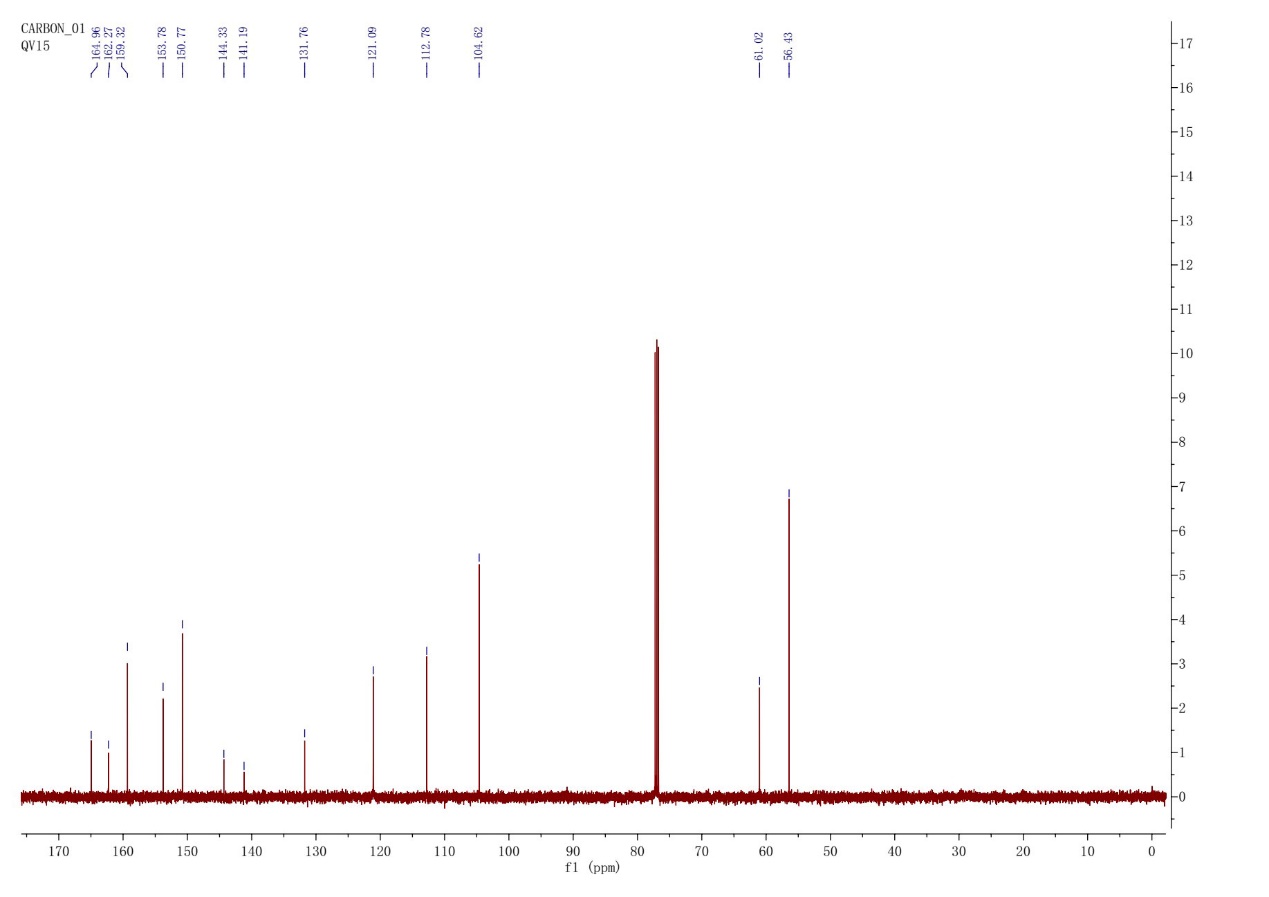
*

*4-(6-(3,4,5-trimethoxyphenyl)pyrimidin-4-yl)-1H-indole (****11s****)*

*
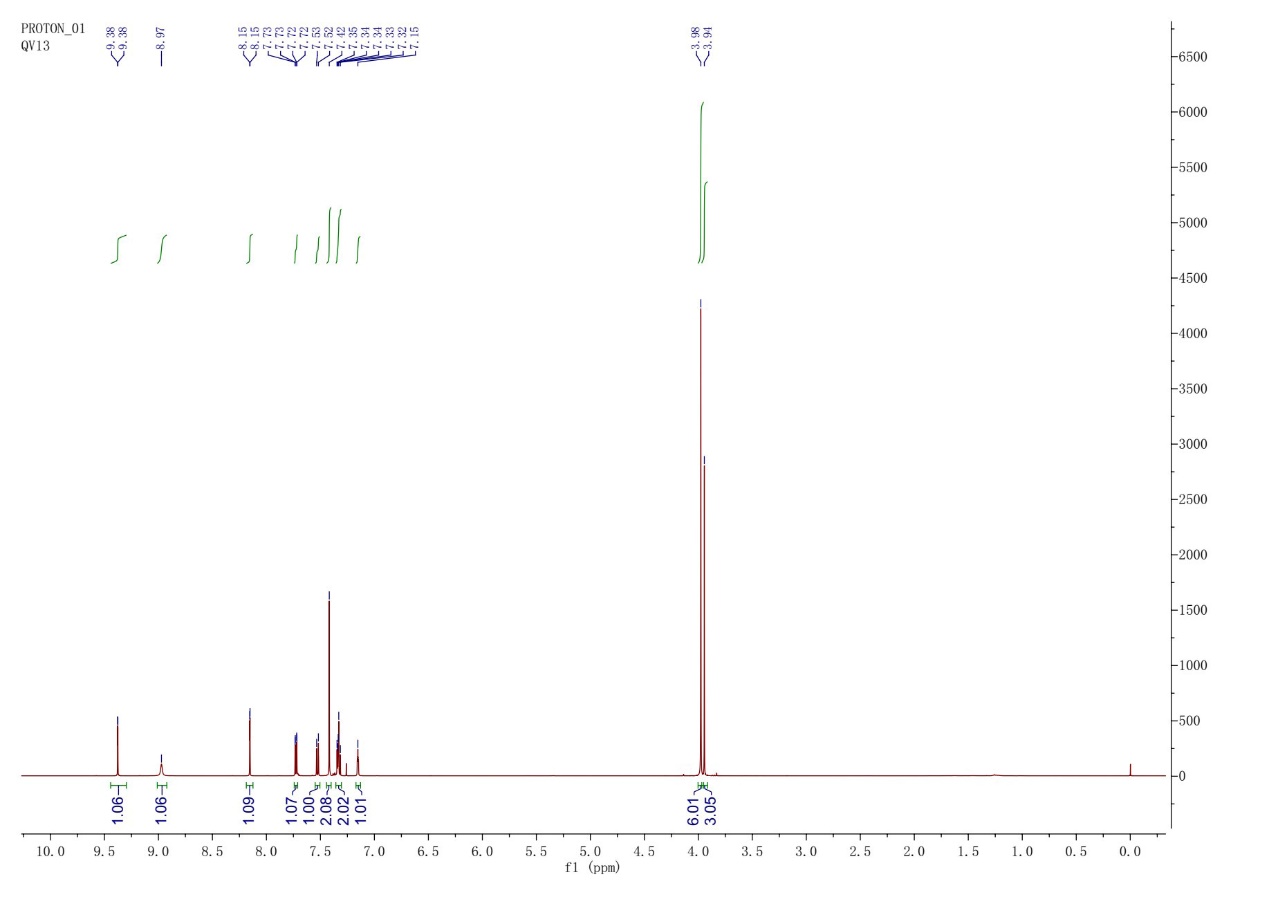
*

*
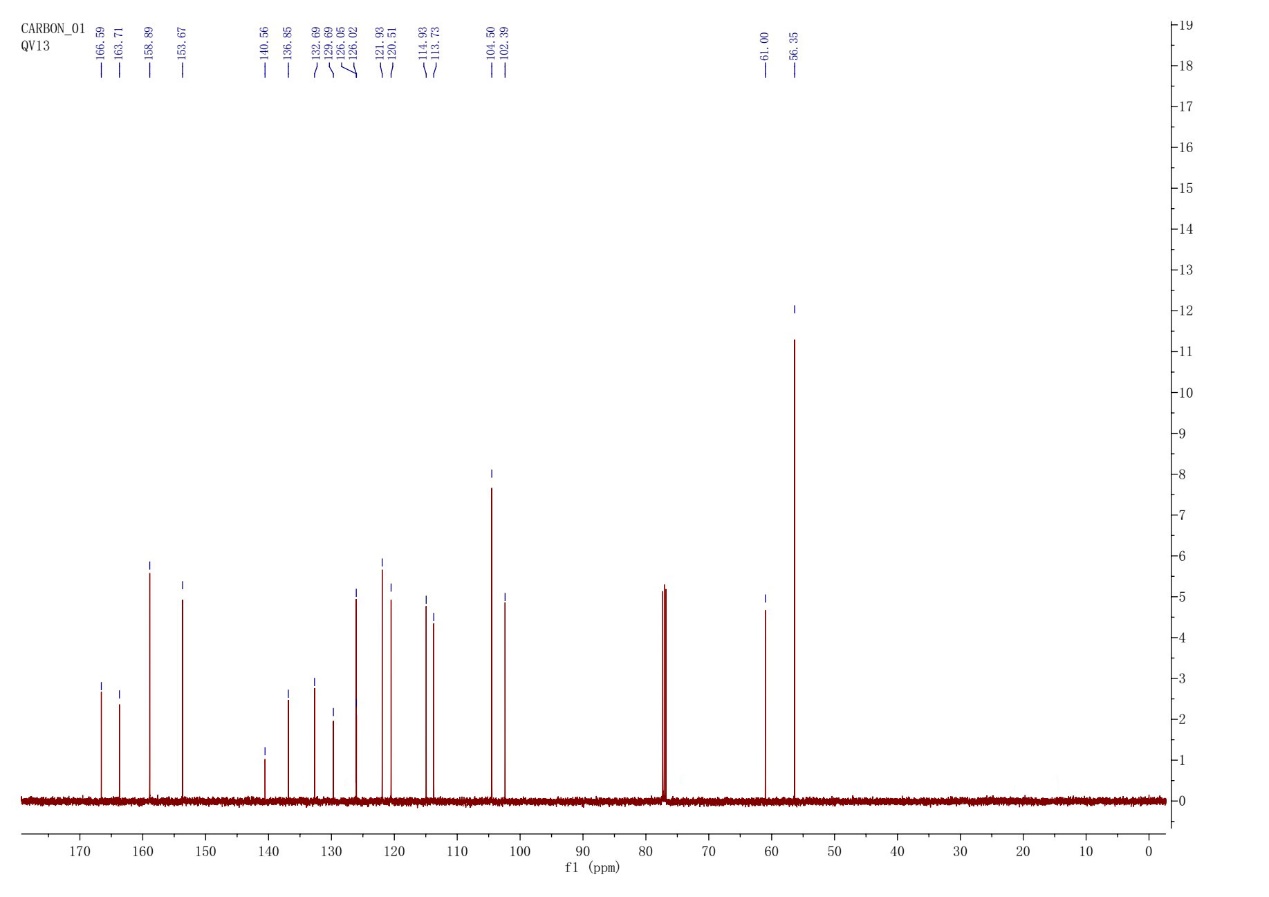
*

*4-(naphthalen-2-yl)-6-(3,4,5-trimethoxyphenyl)pyrimidine (****11t****)*

*
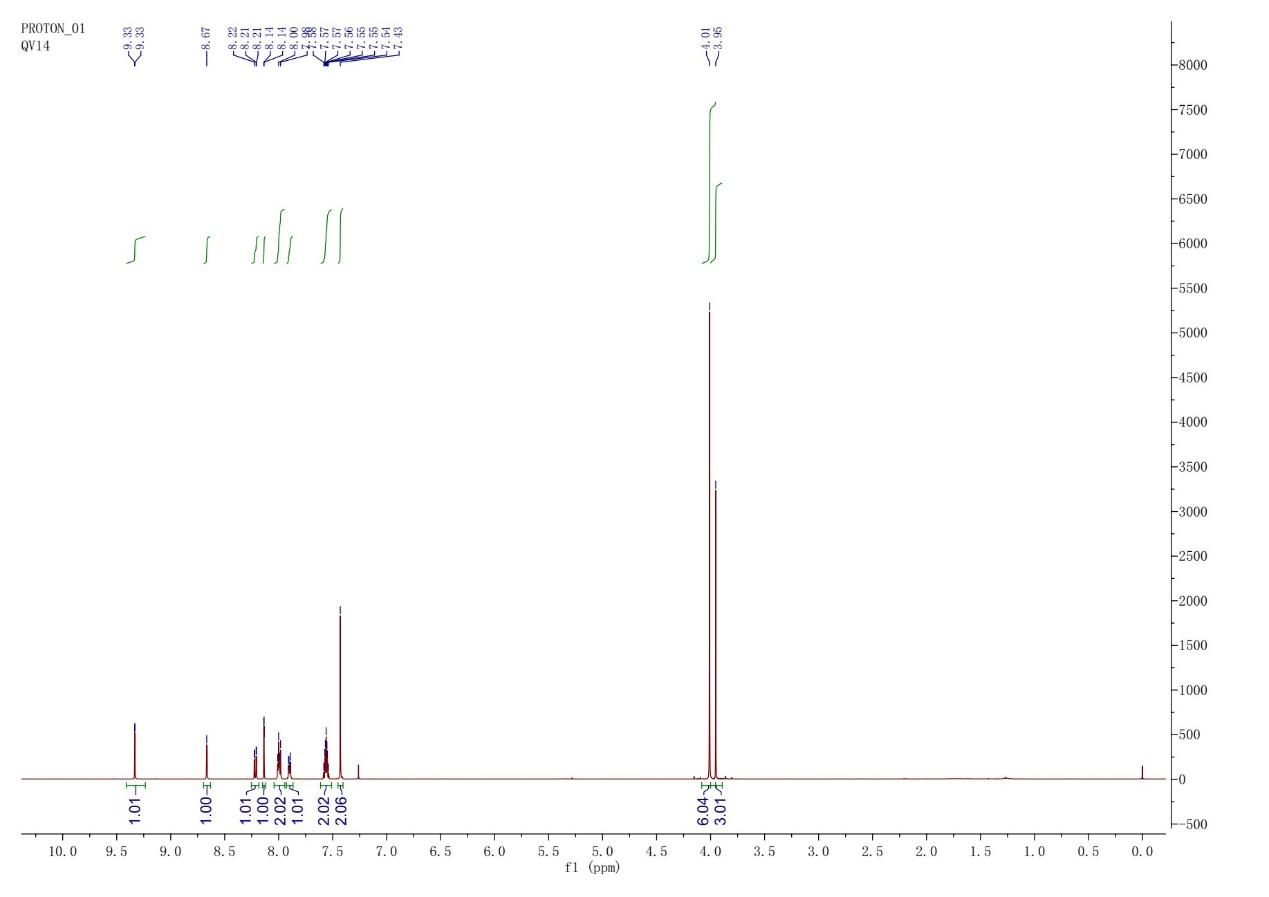
*

*
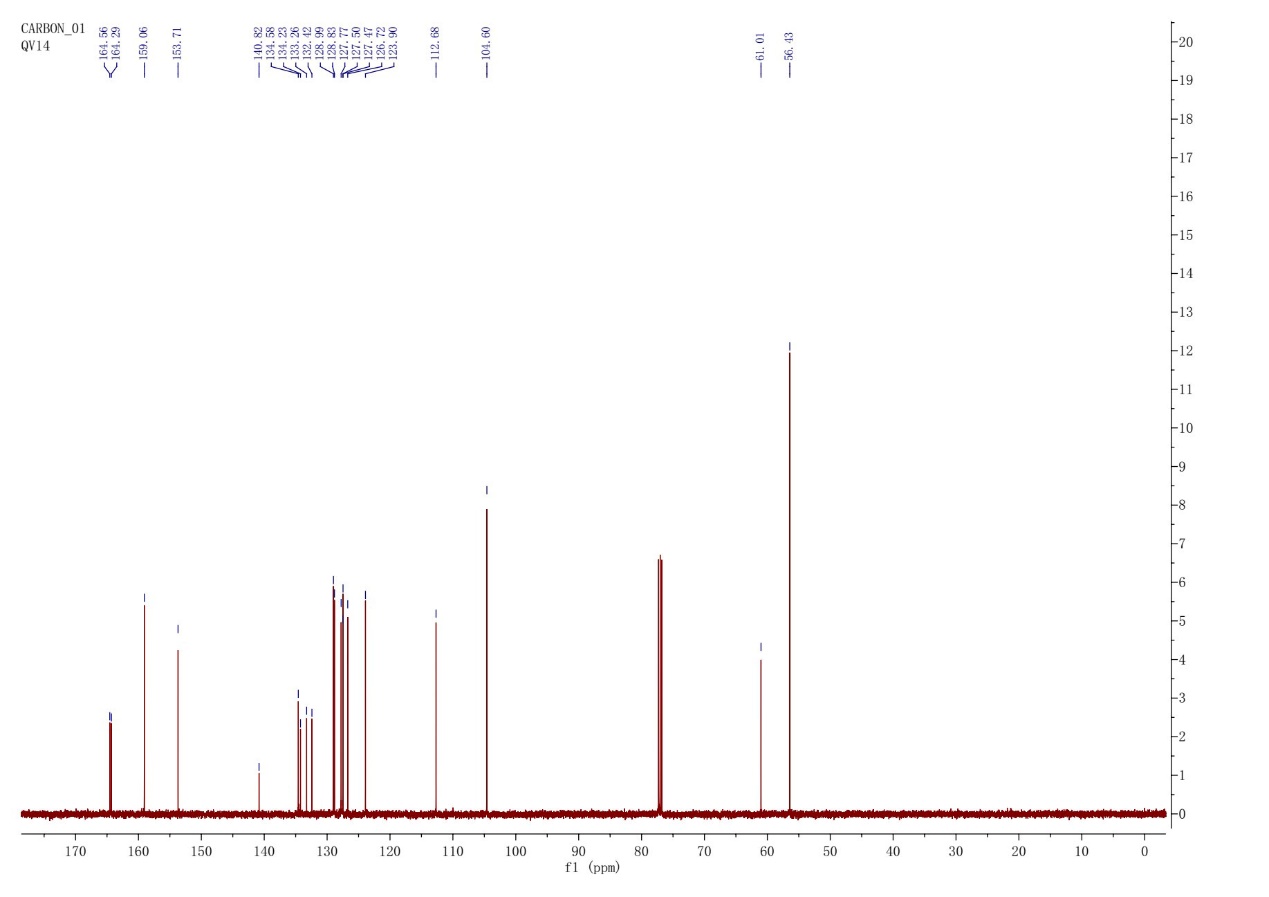
*
